# Copper-Mediated Nitrosation: 2-Nitrosophenolato Complexes and Their Use in the Synthesis of Heterocycles

**DOI:** 10.3390/molecules24224154

**Published:** 2019-11-16

**Authors:** Alexander J. Nicholls, Andrei S. Batsanov, Ian R. Baxendale

**Affiliations:** Department of Chemistry, University of Durham, South Road, Durham DH1 3LE, UK; alexander.j.nicholls@durham.ac.uk (A.J.N.); a.s.batsanov@durham.ac.uk (A.S.B.)

**Keywords:** copper-nitrosophenolato complex, phenols, *C*-nitrosation, sodium nitrite, copper, cycloaddition, heterocyclic, ambient conditions

## Abstract

A simple protocol yielding *ortho*-substituted nitrosophenols from phenols is demonstrated, in the form of copper(II) *bis*(nitrosophenolato) complexes. The developed methodology was applied to a range of substrates, confirming the role of the copper in both the formation and protection of the challenging 1, 2-substitution pattern. Using polymer supported thiourea, the Cu could be stripped from the complexes and thus enabled the isolation or identification of the uncoordinated ligands and their decomposition products, in yields generally low in line with the intrinsic high reactivity of 2-nitrosophenols. The product complexes are useful intermediates as demonstrated in revisiting a formal [4 + 2] cycloaddition with dimethylacetylene dicarboxylate to synthesise bicyclic products in variable yields, revealing the product has a novel structure different from those previously reported in the literature.

## 1. Introduction

Due to challenges in the synthesis and issues with stability including oxidation, dimerisation and condensation, *ortho*-substituted-nitrosophenol compounds are seldom reported in the literature [1]. There are limited reports of other regioisomers having found application, often due to their ease of oxidation to the corresponding nitro moiety [2]. Interestingly, *C*-aromatic nitrosation procedures have superior regioselectivity to their nitration counterparts, with nitrosation occurring almost exclusively at the *para* position of phenols, where possible [3]. This strength however becomes a weakness if the *ortho-*isomer is the desired product. While substrates with particular directing or blocking substituents can be used to force *ortho*- nitrosation to dominate, additional issues pertaining to the fact that 2-nitrosophenol compounds are generally unstable, and that highly functionalised substrates are often not reactive enough to undergo nitrosation [4], inhibit further investigation into this class of compounds. Alternatively, metals such as copper can be used to not only lower the activation barrier of the nitrosation process via generation of a more powerful nitrosating agent, but also act to protect the 2-nitrosophenol products from unwanted oxidation or decomposition pathways through complexation. Having recently reviewed the literature surrounding the preparation of metal-nitrosophenolato complexes [1] and having noted the high potential synthetic value of such chemical approaches, we herein address some literature inconsistencies and clarify the identity of both complexes and further derivatisation products.

Our specific contribution to the field starts with the synthesis and characterisation of a selection of copper complexes including isolating the free ligands from them, we also address some confusion regarding substrate reactivity and finally, verify the products of a particularly intriguing [4 + 2] addition process of the copper complexed ligands with dimethylacetylene dicarboxylate [1,5,6,7].

The atom-efficient, one-pot nitrosophenolato complex formations are performed by reacting substituted phenols in acidic-aqueous conditions, at ambient temperatures. While numerous metals can form 2-nitrosophenolato complexes [1,8], copper salts were the focus of this initial study because they are cheap, readily available and have relatively low toxicity. Many of the 2-nitrosophenolato ligands observed in the complexes would not survive as free compounds under standard atmospheric conditions, however they can be preserved as the complexes long-term (three years of stability demonstrated in this study). Consequently, while the free 2-nitrosophenols are readily released from their parent copper complexes by the action of a suitable copper scavenger, such as thiourea, only some were sufficiently stable to be fully characterised in their free non-complexed form.

One of the first examples of metal-nitrosophenolato complexes is associated with the historic process known as the Baudisch reaction [9,10,11]. Although limited to only electron-rich phenolic substrates, the simplified copper-mediated nitrosation described presently has advantages over this historic method, as it is higher-yielding and requires fewer reagents. As apparent from the literature [1], there is a disagreement as to whether the use of metals in the nitrosation of phenols significantly affects the regioselectivity of the process [1,10,12,13,14]. Our results address this disagreement, but the answer appears very complicated.

## 2. Results and Discussion

After a series of basic scoping reactions, with the specific aim of creating simple and direct conditions for the formation of the copper-nitrosophenolato complexes and their isolation, we settled on the following protocol. Our approach was to dissolve the phenol in a mixture of water and acetic acid, buffered with sodium acetate (Table 1). The copper salt (CuSO_4_) was dissolved in water containing the sodium nitrite, before addition to the phenolic solution dropwise, performing a *C*-nitrosation (Table 1). It was determined under these conditions that the product precipitates and is readily collected by direct filtration. These copper(II) *bis*(2-nitrosophenolato) products are characterised, confirming proposals in the older literature, where proof of structure was not possible at the time [8,9,14]. The crude, filtered product was washed with water, followed by toluene, which was successful in purifying the complexes to a high level in most cases. Upon drying at reduced pressure, the solid compounds were mostly anhydrous (indicated in the experimental); however the complexes can support a variety of coordinating ligands, including water and alcohols, by simply mixing with these solvents or reagents [1]. These complexes with an additional solvate were crystalline, hence allowed single-crystal XRD analysis in several cases, but this was not necessary to purify the compounds.

Most of the substrates tested formed the target complex in yields ranging from acceptable (41%) to good (86%), as supported by mass spectrometry, C, H, N, elemental analysis, IR spectroscopy and where possible, XRD characterisation (**2a, 2c, 2d, 2k**); these were generated from the substrates with either a *para*-substituent or a *meta*-, π-donating substituent on the phenol. Other phenols (**1f, 1l**, **1m**, **1n**, **1o**) also formed a copper adduct (**2f, 2l, 2m, 2o)** that upon first inspection appeared similar to the target complexes, again in generally good yields, initially leading to the belief that the target structure had formed. However, upon detailed characterisation, the data set obtained for these compounds was different to that of the confirmed copper *bis*(2-nitrosophenolato) complexes (e.g., copper *bis*(2-nitroso-4-methylphenolato) **2a,** which was confirmed by XRD analysis). While the IR data and melting (decomposition) temperatures were comparable, the molecular ion could not be identified by mass spectrometry (instead an intense peak relating to the free ligand was observed) and the elemental analysis result suggested the presence of one (or more) additional NO moieties in the molecular formula. Furthermore, the organic compounds isolated following copper scavenging with thiourea had not the 2-nitroso/nitrophenol regiochemistry, but were 4-nitrosophenols. We considered the possibility that the target complex had indeed formed with 2-nitrosophenol ligands, but that the ligands had, upon decomplexation, spontaneously rearranged to the 4-nitrosophenol isomer which was the isolation product, but this would not explain the absence of a molecular peak in the mass spectra or the elemental analysis results. As these compounds failed to form any crystals of X-ray quality, it was not possible to confirm the connectivity of these complexes, but the components present are determined with the data available. There are various donor groups on the nitrosophenol product that could coordinate with the metal centre; one possibility is that the N and O atoms of the nitroso moiety both bond to the metal, as in uranium-nitrosophenolato complexes [15], allowing complexes to form without the need for the nitroso- and phenolic OH groups to be *ortho* to each other, though again the data are inconclusive at this stage. A possibility was that these products are simply a mixture of 4-nitrosophenol alongside inorganic species, but in this case, it would have been possible to separate the organic component by washing the residue with toluene. This was not the case, with the toluene wash removing just small amounts of other organic impurities, suggesting there is some form of interaction between the nitrosophenol and copper component.

Some of the 4-substituted substrates, specifically 4-bromophenol and 4-chlorophenol, proceeded in lower isolated yields to the target product because a side product, the 4-halogeno-2-nitrophenol was produced in significant amounts (e.g., 28% for 4-chlorophenol). An excess of sodium nitrite in the scheme increased the conversion of the starting material and the final yields, however it also increased the chance of over-oxidation of the substrate to the corresponding nitro compound. During the reaction some nitrite is wasted via de-gassing of nitric oxide from the reaction mixture, as can be evidenced by its oxidation in air to brown nitrogen dioxide gas. This can be accounted for by the known propensity of copper to reduce nitrite to nitric oxide [16], as well as the action of phenol on the nitrosonium ion formed, followed by hydrolysis [17]. Poly-nitrosation was prevented in every case without the need for further reaction design, owing to the substantial reduction in aromatic nucleophilicity that accompanies the addition of a nitroso group [2], this is much greater than for the corresponding nitro group addition.

A further scientific endeavour of this work was to investigate the role of copper in terms of catalysing the nitrosation, protecting the products and improving the regioselectivity for *ortho*-nitrosation instead of *para*. This was tested by attempting to perform the *C*-nitrosation of phenols such as 3-chlorophenol (**1i**) in the presence and absence of copper under the same matched conditions. As demonstrated, phenol **1i** was largely inert to direct nitrosation without copper, with only small quantities of a polymeric-type product observed alongside unreacted starting materials. In contrast, under copper-mediated conditions, 3-chlorophenol formed the target complex in high yield (Table 1, 73%), suggesting the copper is indeed a catalyst, this is consistent with indicated results from previous sources [2]. However, both principal product regioisomers: 3-chloro-6-nitrosophenol and 3-chloro-4-nitrosophenol, have never been isolated and thus no supporting proof of structure exists. Our attempts to isolate the free ligand product(s) of this reaction were also unsuccessful. Hence although it has been confirmed that the use of copper does catalyse the reaction and protects the *ortho*-product (with formation of **2i**), it still remains uncertain whether the regioselectivity (*ortho*:*para* selectivity) of the catalysed process is improved. This result for **1i** contrasts with that of the similar 3-methylphenol (**1j**) which formed 4-nitroso-3-methylphenol (**3j(i)** 76%, accompanied by a side product of 4-nitro-3-methylphenol, 12%) in the absence of copper, but in the presence of copper again free 4-nitroso-3-methylphenol **3j(i)** was the major product (~60%), with only a small amount (~9%) of the target copper *bis*(2-nitroso-5-methylphenolato), **2j** Although the chloro atom is weakly π-donating and the methyl is a *σ*-donor, there are additionally steric factors. This is still somewhat confusing because although the chloro substituent could be more demanding than the methyl and thus reducing the likelihood of *para*-nitrosophenol formation by hindrance, the opposite could be argued given the effective radius (2.0 Å) [18] of the methyl substituent is actually a little more than the chloro (van der Waal’s radius of 1.8 Å) [18].

In conclusion, it appears that the presence of copper has little or no effect on the *ortho*-regioselectivity, but nevertheless is valuable because it allows access to the challenging 2-nitrosophenols by protecting them as they form and allows more efficient formation of nitrosophenols from substrates that are not very reactive, provided the substrate’s substituents ensure *ortho*-nitrosation is more favourable than *para*.

### 2.1. Substrate Scope

The substrate scope includes a range of phenols. Alkyl, ether and halo substituents are tolerated, with the highest yields achieved for phenols with activating, electron-donating substituents. The scheme was attempted with catechol and a black powder was isolated, but the product could not be identified. Typically electron-rich aromatics are reactive to *C*-nitrosation processes [2], hence the formation of a copper-nitrosophenolato compound (of either type) is successful for these substrates. The reaction was attempted with 4-cyanophenol and 4-nitrophenol, but no reaction occurred (starting materials were recovered unchanged). 3-hydroxybenzoic acid did form a red solution, as if to suggest some form of *C*-nitrosation was taking place, but after two weeks of reaction time the only solid recovered was unreacted starting material.

**Figure 1:**
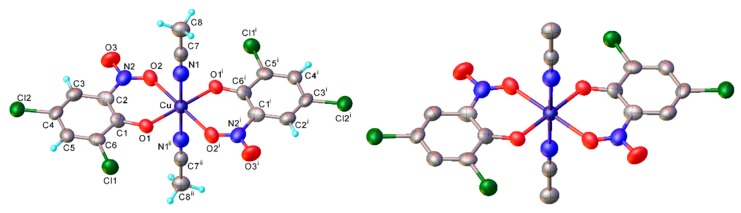
The molecular structure of copper *bis*(2,4-dichloro-6-nitrophenolato) *di*(acetonitrile) (**2k**), shown both with (**left**) the hydrogen atoms simulated using riding models and without (**right**). Symmetry transformations: (i) 1–*x*, –*y*, 1–*z*; (ii) *x*, –*y*, 1–*z*. Bond distances (Å): Cu-O(1) 1.91(1), Cu-O(2) 2.16(1), Cu-N(1) 2.199(6). The alternative positions of nitrosophenol ligands, generated by a 2-fold axis (transformation ii) are omitted for clarity (Figure S3). Thermal ellipsoids are drawn at the 50% probability level.

As discussed, the natural position for nitrosation of phenol remains the *para* position, so copper *bis*(2-nitrosophenolato) complexes will form if the substrate has substituents that improve the favourability of *ortho*-nitrosation, by hindering the *para* site or by overriding electronic selectivity. This would include all 4-substituted phenols and many 4-unsubstituted-3-substituted, provided the 3-substituent is either sufficiently large (as to cause hindrance of the *para*-position) or a strong π-donor (as to activate the *ortho*-position to the phenol). For other phenols, the reaction will produce not a copper (2-nitrosophenolato) complex, but instead a compound derived from a 4-nitrosophenolato ligand. An anomalous result is that from 4-hydroxybenzoic acid; this substrate could be expected to form copper *bis*(2-nitroso-4-carboxylphenolato), but instead yielded a compound that seemingly resembled the other non 2-nitroso compounds. There is one further outlying result, this is associated with the product from 2,4-dichlorophenol, which upon crystallisation had oxidised to the nitro compound (**2k**, Figure 1). This was the only case where the copper failed to protect the nitrosophenol ligand from oxidation to the corresponding nitro derivative; this is a remarkable compound and appears to be the first of its type to be characterised.

### 2.2. Properties of Copper-2-Nitrosophenolato Complexes

**Figure 2:**
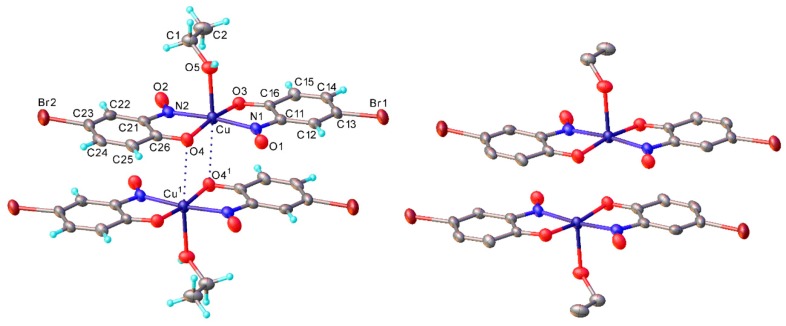
The intermolecular interactions of copper *bis*(4-bromo-2-nitrosophenolato), **2c** crystallised from ethanol-chloroform, shown both with (**left**) the hydrogen atoms simulated using riding models and without (**right**). Mean bond distances Cu-O(phenol) 1.939(5), Cu-N 1.981(6), Cu-O(Et) 2.239(6) Å (cf. in **2a**, 1.954(2), 1.988(2) and 2.236(2) Å, respectively). There is a weak intermolecular Cu-O interaction opposite the ethanol ligand (3.196(6) Å in **2c**, 3.243(2) in **2a**). Primed atoms are generated by an inversion centre.

All of the isolated copper-nitrosophenolato compounds are coloured amorphous powders when obtained directly from the reaction mixture by filtration, and after washing. The complexes decompose (usually turning colourless), rather than providing a melting point, at fairly high temperatures and are all somewhat soluble in polar, organic, coordinating solvents. Most of these complexes could be crystallised, although the addition of a coordinating solvent (e.g., ethanol) was usually required to improve the solubility and grow suitably large crystals. Of the three copper-nitrosophenolato complexes where structures were obtained (**2a, c, d**) all were monoclinic and were of space group P2_1_/c (**2a** and **2d**) or P2_1_/n (Table S1). The solved structures of **2a** and **2c** (Figure 2) show that ethanol coordinates the copper atom at the apical position, complementing the *trans*-square-planar coordination by two nitrosophenolato ligands to square-pyramidal, thus forming a new complex, very intensely coloured in solution. In the solid, the coordination sites *trans* to the ethanol, are occupied by the phenolic O atoms of adjacent molecules, but the Cu…O distances (3.243(2) Å in **2a**, 3.196(6) Å in **2c**) are only slightly shorter than the sum of van der Waals radii [18], while the Cu atom deviates from the N_2_O_2_ basal plane by 0.13 Å towards the ethanol ligand. Only the complex **2d** synthesised from vanillin crystallised without the inclusion of a solvent; here the Cu coordination is completed to a centrosymmetric tetragonal-bipyramidal (a typical tetragonal distortion) by the aldehyde groups of two adjacent molecules (Cu-O 2.578(3) Å) The N-O bond lengths (1.248(2) Å in **2a**, 1.239(8) in **2c**, 1.236(2) in **2d**) are between what would be expected for a N-O single and double bond, suggesting the structure is intermediate between a nitrosophenol and a benzoquinone-monoxime in terms of molecular π-orbitals [19]. Attempts were made to characterise the complexes by NMR spectroscopy. The complexes were soluble in *d*5-pyridine (due to the ability of the pyridine to solubilise the complexes by coordinating to form an extended complex [20]). Unfortunately, no peaks are observed that could be assigned to the product; it’s likely the broadening due to Cu(II) reduces the resolution to baseline levels. However, for some of the **2** series, (for example, **2a**–**d**) peaks that were within the ppm ranges expected were instead visible in the NMR experiments with *d*6-DMSO, but these resonances correlated with the compounds **3a**–**3d**, the oxidised free ligands. Further investigation of the behaviour of the complexes in DMSO revealed that over time, some oxidised ligands were extracted from the complex, with inorganic copper species as a side-product, confirming that the peaks observed by NMR spectroscopy were due to the oxidised ligands and not the product complexes.

### 2.3. Isolation of Organic Ligands from Copper Complexes

For most complexes, the free ligand (or a species to which the free ligand is the reasoned precursor) has been isolated. 2-nitrosophenol compounds are also known to exist in the tautomeric forms; namely, as either the nitrosophenol or the benzoquinone-monoxime (favouring the benzoquinone). The displacement of the nitrosophenols from the complexes presented a range of difficulties because of the strength of the chelated complexes and poor stability of the individual free ligands. In older literature, it is stated that the free nitrosophenols are ‘easily recovered’ by reaction with hydrochloric acid [8], followed by extraction into petroleum ether; this was not found to be a viable method, with only very low yields of mixed aromatic products obtained in most cases. Our preferred approach was to therefore avoid the harsh acidic conditions, by instead displacing the nitrosophenol ligands with a more effective copper-chelator (a polymer-supported thiourea PS-TU, Table 2). In order to confirm the regiochemistry of the organic nitrosophenol product obtained from mixture **2j**, the single crystal XRD structure **3j(i)** was obtained in two different forms, one monoclinic, the other triclinic (Table S1) and no trace of the expected regioisomer (**3j**) was detected by any methods.

The identity and properties of the aromatic compounds isolated varied substantially. For 4-substituted-2-nitrosophenolato complexes, free nitrosophenols were not detected. It is predicted that the free 4-substituted-2-nitrosophenols are unstable and decompose according to a previously proposed literature pathway [3,4], once they are no longer supported by the copper. A publication claimed that these compounds are stable, albeit at very low temperatures [21], although this work has since been retracted. A potential work-around involves deliberately oxidising the nitrosophenol to its related nitrophenol as soon as it is disassociated, by performing the ligand isolation in THF that had been saturated with oxygen gas. Applying this method, low yields of 4-substituted-2-nitrophenols, alongside polymeric tars (containing complex mixtures), were obtained from the 4-substituted-2-nitrosophenolato complexes, suggesting that 4-substituted-2-nitrosophenols were indeed the precursor species (i.e., the original ligands). Indeed, 4-nitrosophenol (or more correctly, 1, 4-benzoquinone monoxime) was also found as the equivalent nitro product, although was inert in a nitrogen atmosphere. The nitrosation agent itself is known to oxidise aromatic nitroso to nitro derivatives in some cases [2], but these results show that this process also occurs for these 4-substituted-2-nitrosophenols with O_2_ as the oxidant. While the 4-substitued-2-nitrosophenols could not be isolated as stable free compounds, their existence as ligands is confirmed via the characterisation of their parent complexes. The crystal structures of three different Cu(II) *bis*(2-nitrosophenolato) compounds (**2a**, **2c** and **2d**) confirm the identity of the ligands, where the ligands themselves could not be identified. The IR bands observed in all of the complexes (N-O stretch: 1550–1625 cm^−1^ and 1400–1500 cm^−1^), also indicate the presence of nitroso bonding.

**Figure 3:**
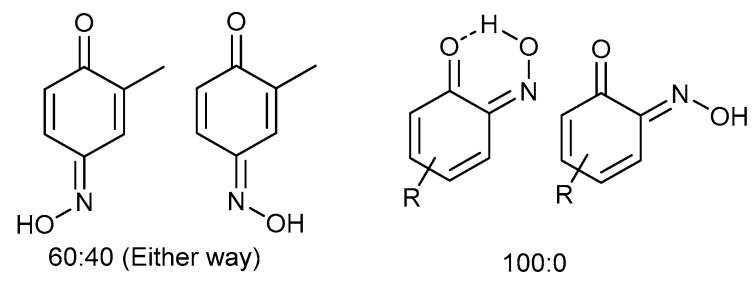
The E/Z isomerism in benzoquinones, with approximate product ratios.

**Figure 4:**
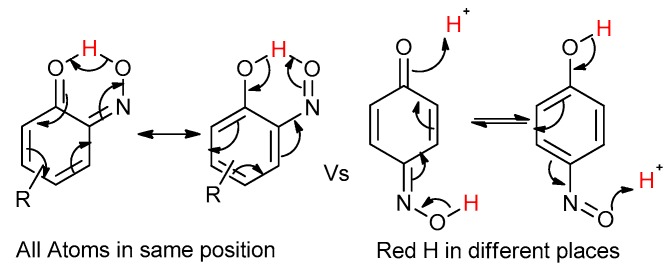
The consideration that the quinone and nitroso structures may be related by resonance if the nitroso is *ortho* to the phenol. Otherwise, a hydrogen atom is in a different position depending on the tautomer.

Even for the stable nitrosophenol compounds, the isolated yields were often relatively low. It might be that only one of the two organic ligands can be successfully displaced by the polymer-supported thiourea for some complexes, giving a maximum yield of 50%. For the *ortho-*benzoquinone-monoxime structures, only one set of resonances is observed by NMR spectroscopy despite the possibility of *E*/*Z* isomerism about the C=N double bond. This is because the formation of an intramolecular H-bond ensures that only one diastereomer is found (Figure 3). This was not the case for the *para*-benzoquinone-monoximes such as 2-methyl-1, 4-benzoquione-monoxime (**3l**), for which both stereoisomers were observed; there was even a third structure observed for 2-chloro-1, 4-benzoquinone-monoxime (**3m**), believed to be the nitrosophenol tautomer albeit in small quantities. The reason that no second (nitrosophenol) tautomer is observed for the *ortho*-ligands could be that the H atom is in the same place whichever tautomer and hence the structures are related by resonance (Figure 4).

The nitrosophenols can be divided into three categories (Table 3) based upon their structure and stability. Aromatics without a *para*-activating substituent at the 4-position relative to the nitroso, are unstable. This is logical, because the nitroso group is electron withdrawing and the carbon it is bonded to becomes electron deficient. By having an electron-donating group *para* to the nitroso, the electron deficient carbon is stabilised and the free compounds are thus much easier to isolate. Others oxidise in aerobic conditions (second category) or additionally decompose to a complex, polymeric mixture (third category) at room temperature.

### 2.4. Cycloadditions of Dimethylacetylene Dicarboxylate with the 2-Nitrosophenols (Starting with the Cu bis(nitrosophenolato) Complexes)

An intriguing use of the copper nitrosophenolato complexes is as diene substrates in cycloadditions with powerful dienophiles, i.e., dimethylacetylene dicarboxylate and was first reported by McKillop and Sayer [22]. In this current study, full characterisation of six such cycloaddition products has been provided for the first time, including new compounds synthesised from copper(II) *bis*(5-methyl-2-nitrosophenol) and copper(II) *bis*(2-methoxy-6-nitroso-4-formylphenol). These bicyclic products have unique functionality that may have interesting biological activity. The successful synthesis of such heterocycles both helps confirm the identity of the parent complexes as well as providing useful cycloaddition products. The products were crystalline although preferred to form clusters of very small shapes, making single-crystal XRD analysis challenging. Nevertheless, structures were solved for **4a**, **4b** and **4d**, each having a different space group (Table S1).

**Figure 5:**
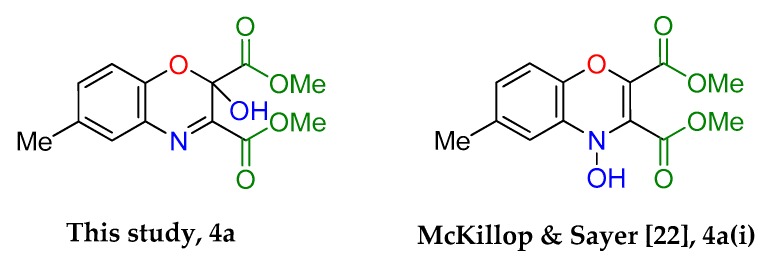
The assigned structure of **4a** obtained in this study compared to the literature assigned structure for the same reaction.

The yields (Table 4) were variable; copper(II) *bis*(4-methyl-2-nitrosophenolato) was synthesised from greater dilution, discouraging decomposition of the copper starting material. The Scheme of Table 4 was attempted using the compounds synthesised from 2-substituted phenols and that from phenol itself (**2l**, **2m**, **2n, 2o**), but the result was a complex mixture, with no individual compounds identified, further supporting the assignment that these compounds (**2l**, **2m**, **2n, 2o**) are not copper-2-nitrosophenolato complexes. The previously reported literature yields are much higher than measured in this study [22], however, as the quoted melting points are lower than found in this study and because no further characterisation data was provided, it is uncertain that these literature yields refer to compounds of high purity, as in this publication. As well as the yields, the structure reported is different (Figure 5). We note the logic in the original assignment and conclude that without the use of XRD analysis, distinguishing the structures would be challenging by other means. Having isolated the product **4** we were also careful to re-evaluate the reaction mixture and to identify that at no stage was the alternative hydroxyl regioisomer detectable in solution. We also note that the material isolated upon completion of the reaction matches that following crystallisation structure meaning no rearrangement takes place during preparation of the crystals. Therefore, herein, we wish to correctly identify the sole products of the cycloaddition process as the compound based upon the connectivity depicted in structure **4**.

## 3. Materials and Methods 

### 3.1. Synthesis of Copper-Nitrosophenolato Compounds

The phenolic starting material (**1a**–**1o**, 50 mmol) was dissolved in 25 mL of water and 15 mL acetic acid. To this mixture was added solid sodium acetate trihydrate, gradually, until the pH was 4. Copper sulfate pentahydrate (25 mmol, 6.25 g) and sodium nitrite (125 mmol, 8.65 g) were dissolved in water (250 mL) and this solution was introduced dropwise to the solution of phenolic starting material. The mixture was stirred at ambient temperature for at least 3 days and left to stand without mixing for a further 24 h. The product was collected by filtration and washed with water (3 × 50 mL), toluene (2 × 50 mL) and finally a portion of ethyl acetate (20 mL), then dried under reduced pressure. The copper complexes (**2a**–**2o**) were of acceptable purity at this stage and characterised by ASAP MS, IR spectroscopy and elemental analysis. For the purpose of growing crystals suitable for XRD analysis, the powder obtained was dissolved in a 1:1 mixture of boiling alcoholic and polar-organic solvents (specified), then left to cool to room temperature. The resulting crystals were collected by filtration and washed with hexane (10 mL), giving crystalline copper complexes (**2a**–**2o**). Yields refer to pure, washed, amorphous products, not recrystallised adducts. The spectral data is shown below and the graphical spectra and data (NMR, IR, MS and XRD) can be found in the supplementary information.

**Copper(II) *bis*(4-methyl-2-nitrosophenolato) (2a)** [23]**:** Recrystallised from chloroform-ethanol to yield a deep purple solid. Yield: 82%, 7.2 g. C_14_H_12_O_4_N_2_Cu. ASAP-MS: *m*/*z* 336.0 ([M + H]^+^, 100%), 138.1 ([C_7_H_7_O_2_N + H]^+^, 2) HR-MS calculated for C_14_H_13_N_2_O_4_^63^Cu 336.0171, found: 336.0167. (−1.2 ppm, −0.4 mDa). IR (neat) υ_max_/cm^−1^ 2971 (m, C-H), 1739 (s, C=O), 1365 (s, N=O), 1218 (m). Elemental (C, H, N) analysis found: C 49.75% (50.07), H 3.60% (3.66), N 8.42% (8.34). *Crystal data:* 1957008; C_14_H_12_O_4_N_2_Cu·C_2_H_6_O, f.w. 381.86, T = 120 K, monoclinic, *a* = 10.1875(8), *b* = 6.9915(5), *c* = 22.2994(17) Å β = 91.781(3)°, *V* = 1587.5(2) Å^3^, space group *P*2_1_/*c* (no. 14), *Z* = 4, 22237 reflections (3840 unique, *R*_int_ = 0.050), *R*_1_ = 0.034, *wR*_2_ = 0.081 The structure is essentially identical with the earlier room-temperature determination [22]. M.p. 218.1–218.8 °C (decomposition).

**Copper(II) *bis*(4-Chloro-2-nitrosophenolato) (2b)** [24]**:** Recrystallised from chloroform-ethanol to yield a purple solid. Yield: 55%, 5.4 g. C_12_H_6_N_2_O_4_Cl_2_Cu. ASAP-MS: *m*/*z* 377.9 ([M + H]^+^, 95%), 158.1 ([C_6_H_3_O_2_NCl H]^+^, 25). HR-MS calculated for C_12_H_6_N_2_O_4_^35^Cl_2_^63^Cu 374.9012, found: 374.9001. (−2.9 ppm, −1.1 mDa). IR (neat) υ_max_/cm^−1^ 3017 (br, C-H). 1521 (s, N=O), 1489 (s, N=O), 1332 (m, N=O), 1181 (m, C-O). Elemental (C, H, N) analysis found: C 38.37% (38.25), H 1.65% (1.61), N 7.41% (7.44). M.p. 219.3–220.5 °C (decomposition).

**Copper(II) *bis*(4-Bromo-2-nitrosophenolato) (2c)** [8]**:** Recrystallised from chloroform-ethanol to yield a purple solid. Yield: 41%, 4.8 g. C_12_H_7_N_2_O_4_Br_2_Cu ASAP-MS: *m*/*z* 463.8 ([M + H (^79^Br)]^+^ 100%) 203.9 ([(C_6_H_4_O_2_N_1_^79^Br) + H]^+^, 8). HR-MS calculated for C_12_H_7_N_2_O_4_^79^Br_2_^63^Cu 463.8069, found: 463.8076. (+1.5 ppm, +0.7 mDa). IR (neat) υ_max_/cm^−1^ 3087 (m, C-H), 1601 (m, N=O), 1491 (s, N=O), 1315 (m, N=O), 1166 (m, C-O). Elemental (C, H, N) analysis found: 29.57% (30.06), 1.27% (1.30), 5.67% (6.02). *Crystal data:* CCDC 1957005; C_12_H_6_O_4_N_2_CuBr_2_·C_2_H_6_O, f.w. 511.62, T = 100 K, monoclinic, a = 7.0128(16), *b* = 10.020(2), *c* = 23.311(5) Å, β = 93.502(5)°, *V* = 1635.0(6) Å^3^, space group *P*2_1_/*n* (no. 14), *Z* = 4, 19075 reflections (4121 unique, *R*_int_ = 0.081), *R*_1_ = 0.084, *wR*_2_ = 0.240. M.p. 241–255 °C (decomposition).

**Copper(II) *bis*(2-methoxy-6-nitroso-4-formylphenolato) (2d):** Recrystallised from chloroform-methanol to yield a dark purple solid. Yield: 63%, 7.0 g. C_16_H_12_O_8_N_2_Cu ASAP-MS: *m*/*z* 424.0 ([M + H]^+^, 100%) 203.9 ([(C_8_H_7_O_4_N_1_) + H]^+^, 8). HR-MS calculated for C_16_H_12_N_2_O_8_^63^Cu 423.9968, found: 423.9965. (−0.7 ppm, −0.3 mDa). IR (neat) υ_max_/cm^−1^ 3463 (w, br), 3062 (w, C-H), 1687 (s, C=O), 1607 (w, N=O), 1519 (s, N=O), 1388 (m, N=O), 1273 (s, N=O), 1141 (s), 1091 (m), 654 (m). Elemental (C, H, N) Analysis found: 42.86% (43.30), 3.34% (3.63), 6.31% (6.45) (compound observed was the monohydrate). *Crystal data*: CCDC 1957006; C_16_H_12_O_8_N_2_Cu, f.w. 423.82, T = 120 K, monoclinic, *a* = 6.4741(5), *b* = 12.0560(9), *c* = 10.1090(7) Å, β = 103.595(3)°, *V* = 766.9(1) Å^3^, space group *P*2_1_/*c* (no. 14), *Z* = 2, 16527 reflections (2239 unique, *R*_int_ = 0.047), *R*_1_ = 0.037, *wR*_2_ = 0.101. M.p. = 269.2–271.7 °C (decomposition).

**Copper(II) *bis*(2-methoxy-6-nitroso-4-ethenylphenolato) (2e):** Dark-green solid that failed to crystallise from various solvent mixtures. Yield: 72%, 7.7 g. CuC_18_H_16_N_2_O_8_. IR (neat) υ_max_/cm^−1^ 3397 (w, br), 2754 (w, C-H), 1596 (m, N=O), 1535 (m, N=O), 1380 (s, N=O), 1227 (s, N=O), 1112 (m, C-O), 800 (s), 693 (m), 504 (m). Elemental (C, H, N) Analysis found: 53.17% (53.39), 4.64% (4.93), 6.46% (6.23). M.p. 161–172 °C (decomposition).

**Copper(II) *bis*(2-nitroso-4-carboxylphenolato)(NOH)_2_ (2f):** Pale-purple solid that failed to crystallise from various solvent mixtures. Yield: 71%, 8.4 g. CuC_14_H_8_N_4_O_10_. IR (neat) υ_max_/cm^−1^ 3247 (m, br, O-H), 1598 (s, N=O), 1474 (m, N=O), 1294 (m, N=O), 1148 (s, C-O), 993 (s), 836 (s), 648 (m), 416 (s). Elemental (C, H, N) Analysis found: 37.28% (36.73), 2.69% (2.20), 12.67% (12.24). m.p. 161–172 °C (decomposition). 

**Copper(II) *bis*(2-nitroso-5-(diethylamino)phenolato) (2g)** [25]**:** Purple solid that failed to crystallise in a range of solvent mixtures. Yield: 40%, 4.1 g. CuC_20_H_26_N_4_O_4_. ASAP-MS: *m*/*z* 195.1 ([C_10_H_14_O_2_N_2_]^+^ 100%). IR (neat) υ_max_/cm^−1^ 3057 (w, C-H), 1588 (m, N=O), 1442 (m, N=O), 1372 (s, N=O), 1227 (s, N=O), 1217 (s, N=O), 1100 (m, C-O), 1014 (m), 925 (m), 653 (m), 500 (m). M.p. > 300 °C.

**Copper(II) *bis*(3-methoxy-6-nitrosophenolato) (2h)** [26]**:** Orange solid that failed to crystallise in various solvent mixtures. Yield: 86%, 8.3 g. CuC_14_H_12_N_2_O_6_ IR (neat) υ_max_/cm^−1^ 3405 (m, br), 3072 (w, C-H), 1595 (m, N=O), 1421 (m, N=O), 1379 (s, N=O), 1227 (s, N=O), 1206 (s, N=O), 1112 (m, C-O), 1029 (m), 856 (m), 693 (m), 503 (m). ASAP-MS: *m*/*z* 368.0 ([M + H]^+^, 100%), 154.0 ([C_7_H_7_O_3_N + H]^+^, 8). HR-MS calculated for C_14_H_13_N_2_O_6_^63^Cu 367.0009, found: 366.9991. (−4.9 ppm, mDa −1.8). Elemental Analysis found: C 43.46% (43.57), H 3.75% (3.66), 7.34% (7.26). M.p. 137–141 °C (decomposition).

**Copper(II) *bis*(3-chloro-6-nitrosophenolato) (2i):** Red solid that failed to crystallise in various solvent mixtures. Yield: 73%, 7.2 g. C_12_H_7_N_2_O_4_Cl_2_Cu. ASAP-MS: *m*/*z* 377.9 ([M + H]^+^, 100), 158.0 ([C_6_H_3_O_2_NCl + H]^+^, 91). HR-MS calculated for C_12_H_7_N_2_O_4_^35^Cl_2_^63^Cu 375.9076, found: 375.9081 (+1.3 ppm, mDa +0.5). IR (neat) υ_max_/cm^−1^ 3351 (br, C-H), 1624 (m, N=O), 1394 (s, N=O), 1248 (m, N=O), 1112 (m, C-O). Elemental (C, H, N) Analysis found: C 39.91% (38.25), H 2.36% (1.61), N 7.48% (7.44). M.p. 153.2–154.0 °C (decomposition).

**Copper(II) *bis*(5-methyl-2-nitrosophenolato) (2j): Isolated mixture with 3-methyl-1,4- benzoquinone monoxime (3j).** Brown solid that failed to crystallise. Conversion >% estimated by derivatisation to the heterocyclic product (**4j**). C_14_H_12_O_4_N_2_Cu. ASAP-MS: *m*/*z* 334.99 ([M]^+^, 100%). IR (neat) υ_max_/cm^−1^ 2773 (m, C-H), 1632 (m, N=O), 1401 (s, N=O), 1116 (m, C-O).

**Copper(II) *bis*(2,4-dichloro-6-nitrophenolato) (2k):** Recrystallised from chloroform-acetonitrile to yield a black solid. Yield: 55%, 6.56 g. IR (neat) υ_max_/cm^−1^ 3082 (w, C-H), 1607 (m, N=O), 1536 (s, N=O), 1447 (m, N=O), 1336 (m, N=O), 1243 (s, N=O), 1151 (s, O-H), 890 (m), 773 (m), 682 (s). *Crystal data:* CCDC 1957007; C_12_H_4_O_4_N_2_Cl_4_Cu·2C_2_H_3_N, f.w. 559.62, T = 120 K, orthorhombic, *a* = 20.491(2), *b* = 7.5407(7), *c* = 13.2872(14) Å, *V* = 2053.1(4) Å^3^, space group *Cmce* (no. 64), *Z* = 4, 8029 reflections (922 unique, *R*_int_ = 0.073), *R*_1_ = 0.045, *wR*_2_ = 0.118. M.p. = 223.3–225.0 °C (decomposition).

**Copper(II) *bis*(2-nitroso-6-methylphenolato)(nitroso) (2l):** Brown solid that failed to crystallise from various solvent mixtures. Yield: 57%, 5.3 g. CuC_14_H_12_O_5_N_3_. ASAP-MS: *m*/*z* 124.0 ([C_6_H_5_O_2_N + H]^+^, 100%). IR υ_max_/cm^−1^ 3264 (m, br, C-H), 1595 (m, N=O), 1429 (s, N=O), 1282 (s), 1111 (m, C-O). Elemental (C, H, N) analysis found: C 45.16% (45.96), H 3.67% (3.31), N 12.28% (11.49). M.p. = 161.5–163.8 °C (decomposition).

**Copper(II) *bis*(2-chloro-4-nitrosophenolato)*di*(nitroso) (2m):** Brown solid that failed to crystallise from various solvent mixtures. Yield: 72%, 7.3 g. CuC_12_Cl_2_H_6_O_5_N_3_. ASAP-MS: *m*/*z* 158.0 ([C_6_H_3_O_2_NCl + H]^+^, 94). IR (neat) υ_max_/cm^−1^ 2817 (m, C-H). 1623 (m, N=O), 1448 (s, N=O), 1302 (m, N=O), 1116 (m, C-O). d.p. 154–158 °C. Elemental (C, H, N) analysis found: C 33.16 (32.86), H 1.84 (1.83), N 11.29 (12.77). M.p. = 154.7–156.2 °C (decomposition).

**Copper(II) *bis*(4-nitroso-2-carboxylphenolato)(nitroso) isolated mixture (2n):** Beige solid that failed to crystallise. Yield: 9.6 g. Formula not assigned. ASAP-MS: *m*/*z* 168.0 ([C_7_H_5_O_3_N + H]^+^, 29). IR υ_max_/cm^−1^ 3382 (w, br, O-H), 1601 (m, N=O), 1455 (s, N=O), 1151 (s, C-O), 828 (m), 644 (s) 575 (s). Elemental (C, H, N) analysis found: C 37.78%, H 2.93%, N 15.59%. M.p. = 243–251 °C (decomposition).

**Copper(II) *bis*(4-nitrosophenolato)*di*(nitroso) (2o):** Red solid that failed to crystallise in various solvent mixtures. Yield: 72%, 6.1g. CuC_12_H_8_O_6_N_4_. ASAP-MS: *m*/*z* 124.0 ([C_6_H_5_O_2_N + H]^+^, 100%). IR (neat) υmax/cm^−1^ 3248 (s, br, O-H). 1598 (m, N=O), 1506 (m, N=O), 1293 (m, N=O), 1147 (m, br, C-O). Elemental (C, H, N) analysis found: C 37.94% (39.19), H 2.72% (2.19), N 13.52% (15.23). M.p. = 163–170 °C (decomposition).

### 3.2. Isolation of Organic Ligands from Complexes

The copper-nitrosophenolato starting material (**2a–2i**) (1.0 g) was dissolved in acetone (40 mL) and the resulting mixture was stirred with polymer-supported thiourea (10 g) and 4 Å molecular sieve (0.5 g) under a nitrogen atmosphere at ambient temperature for 24 h. The solid polymer-supports were removed by filtration and the filtrate passed-through a bed of silica (1 cm depth), to remove any dissolved copper-containing species. The resultant filtrate solution was evaporated to dryness. Pure nitroso products were obtained in the case of **3h**, **3i**, **3j** and **3o** (with a 10% 4-nitrophenol impurity), for others, the organic residue was purified by column chromatography (hexane-ethyl acetate mixtures, various ratios), giving low yields of stated aromatic products. For nitroso products that were too unstable for characterisation after attempting the procedure, the isolation was instead performed in THF (40 mL) saturated with O_2_ gas, rather than acetone, to give low yields of oxidised nitro products (**3a**, **3b**, **3c**, **3d**) and decomposition products.

**4-Methyl-2-nitrophenol (3a)** [27]**:** Eluent: hexane-ethyl acetate (4:1) to yield a brown solid. Yield: 14%, 6.1 g. ^1^H NMR (400 MHz; CDCl_3_) 10.46 (1H, s, O*H*), 7.92 (1H, m, Aryl-*H*), 7.42 (1H, dd, J 8.0, 2.0, Aryl-*H*), 7.08 (1H, d, J 8.8, Aryl-*H*), 2.37 (3H, s, C*H*_3_). δ_C_(400 MHz; DMSO) 150.6 (*C*1), 136.7 (*C*2), 136.5 (*C*4), 129.1 (*C*5), 125.0 (*C*3), 119.5 (*C*6), 19.7 (*C*H_3_). ASAP-MS: *m*/*z* 153.1 (M^●+^, 100%). HR-MS calculated for C_7_H_7_NO_3_ 153.0405, found: 153.0400. (−3.3 ppm, −0.5 mDa).

**4-Chloro-2-nitrophenol (3b)** [27]**:** Eluent: hexane-ethyl acetate (4:1) to yield a beige solid. Yield: 5%, g. ^1^H NMR (400 MHz; CDCl_3_) 10.49 (1H, s, O*H*) 8.11 (1H, d, J 2.8, Aryl-*H*), 7.54 (1H, dd, J 8.8, 2.8, Aryl-*H*), 7.15 (1H, d, J 8.8, Aryl-*H*). ^13^C NMR (100 MHz; DMSO) 153.7 (*C*1), 137.7 (*C*2), 135.8 (*C*4), 125.5 (*C*5), 124.4 (*C*3), 121.5 (*C*6). LC-MS: *m*/*z* 172.0 ([M − H]^−^, 100%).

**4-Bromo-2-nitrophenol (3c)** [27]**:** Eluent: hexane-ethyl acetate (4:1) to yield an orange solid. Yield: 9%, g. ^1^H NMR (400 MHz; DMSO) 8.27 (1H, d, J 2.4, Aryl-*H*), 7.67 (1H, dd, J 8.8 2.4, Aryl-*H*) 7.10 (1H, d, J 8.8, Aryl-*H*). ^13^C NMR (100 MHz; CDCl_3_) 154.1 (*C*1), 140.4 (*C*2), 134.1 (*C*4), 127.3 (*C*5), 121.8 (*C*3), 111.7 (*C*6). LC-MS *m*/*z* 216.3 ([M − H]^−^, 100%).

**2-Nitro-6-methoxy-4-formylphenol (3d)** [28]**:** Recrystallised from ethanol to yield an orange solid. Yield: 21%, g. ^1^H NMR (400 MHz, DMSO-*d*_6_) δ 9.87 (s, 1H, *H*CO), 8.10 (d, *J* = 1.8 Hz, 1H, Aryl-*H*), 7.62 (d, *J* = 1.8 Hz, 1H, Aryl-*H*), 3.96 (s, 3H, OC*H*_3_). ^13^C NMR (101 MHz, DMSO-d_6_) δ 190.91, 150.54, 148.23, 137.54, 127.28, 121.33, 113.03, 57.26.

**3-(Diethylamino)-6-nitrosophenol (3g)** [29]**:** Brown crystals that required no further purification. Yield: %, g. ^1^H NMR (400 MHz, DMSO-d_6_) δ 7.30 (d, J = 9.8 Hz, 2H, Aryl-*H*), 6.89 (d, J = 9.8 Hz, 1H, Aryl-*H*), 5.74 (s, 1H, O*H*), 3.74–3.50 (m, 4H, C*H*_2_), 1.27–1.02 (m, 6H, C*H*_3_). ^13^C NMR (101 MHz, DMSO) δ 169.25, 157.58, 149.61, 134.89, 115.81, 95.52, 46.08, 30.05. LC-MS *m*/*z* 195.1 ([M − H]^−^, 100%). HR-MS calculated for C_10_H_14_O_2_N_2_ 195.1134, found: 195.1152 (9.2 ppm, 1.8 mDa).

**3-Methoxy-6-nitrosophenol (3h)** [30]**:** Eluent: hexane-ethyl acetate (4:1) to yield a green solid. Yield: 49%, g. LC-MS *m*/*z* 154.7 ([M + H]^+^, 100%), 152.4 ([M − H]^−^, 100). ^1^H NMR (400 MHz; CDCl_3_) 7.66 (1H, d, J 10.0, Aryl-*H*), 6.59 (1H, dd, J 10.4, 2.0, Aryl-*H*), 6.09 (1H, s, Aryl-*H*) 3.94 (3H, s, O-C*H*_3_). ^13^C NMR (100 MHz; CDCl_3_) 171.3 (*C*6), 170.1 (*C*1), 150.7 (*C*3), 135.3 (*C*4), 117.93 (*C*2), 100.56 (*C*5), 56.66 (*C*H_3_). HR-MS calculated for C_7_H_8_N_1_O_3_ 154.0504, found: 154.0504. (0.0 ppm, 0.0 mDa).

**3-Nitroso-4-methylphenol (3j):** Eluent: hexane-ethyl acetate (4:1) to yield an orange solid. Yield: % not applicable as starting material was an isolated mixture, 715 mg. ^1^H NMR (400 MHz; DMSO) 8.27 (1H, d, J 2.4, Aryl-*H*), 7.67 (1H, dd, J 8.8 2.4, Aryl-*H*) 7.10 (1H, d, J 8.8, Aryl-*H*). ^13^C NMR (100 MHz; CDCl_3_) 154.1 (*C*1), 140.4 (*C*2), 134.1 (*C*4), 127.3 (*C*5), 121.8 (*C*3), 111.7 (*C*6). LC-MS *m*/*z* 138.1 ([M + H]^+^, 100%). HR-MS: calculated for C_7_H_8_NO_2_ 138.0555, found: 138.0555 (0.0 ppm, 0.0 mDa). *Crystal data:* C_7_H_7_O_2_N, f.w. 137.14, studied as two polymorphs, *viz.* α-**3j** (from hexane/ethylacetate), CCDC 1957003, T = 100 K, triclinic, *a* = 7.2648(8), *b* = 7.3201(8), *c* = 12.8572(14) Å, α = 88.333(2), β = 78.713(2), γ = 84.279(2)°, *V* = 667.1(1) Å^3^, space group *P*$\bar{1}$ (no. 2), *Z* = 4, 9388 reflections (3893 unique, *R*_int_ = 0.050), *R*_1_ = 0.056, *wR*_2_ = 0.159; β-**3j** (from chloroform/methanol), CCDC 1957009, T = 120 K, monoclinic, *a* = 12.8007(13), *b* = 7.7538(8), *c* = 13.8767(14) Å, β = 107.805(4)°, *V* = 1311.4(2) Å^3^, space group *P*2_1_/*c* (no. 14), *Z* = 8, 16949 reflections (3000 unique, *R*_int_ = 0.036), *R*_1_ = 0.037, *wR*_2_ = 0.102.

**3-Nitro-4-methylphenol (3j(i))** [27]**:** Eluent: hexane-ethyl acetate (9:1) to yield a brown solid. Yield: 9%, g. ^1^H NMR (400 MHz; CDCl_3_) 9.45 (1H, s, O*H*) 8.08 (1H, d, J 2.8, Aryl-*H*), 8.02 (1H, dd, J 8.8, 2.8, Aryl-*H*), 6.88 (1H, d, J 8.8, *H*_C_). LC-MS *m*/*z* 153.1 (M^●+^, 100%). HR-MS calculated for C_7_H_7_NO_3_ 153.0405, found: 153.0400. (−3.3 ppm, mDa −0.5).

**2,4-Dichloro-6-nitrophenol (3k)** [31]**:** Recrystallised from ethanol to yield an orange solid. Yield: 49%, g. ^1^H NMR (400 MHz; CDCl_3_) 10.96 (1H, s, O*H*), 7.92 (1H, s, Aryl-*H*), 7.62 (1H, s, Aryl-*H*), ^13^C NMR (100 MHz, CDCl_3_) 150.1, 137.7, 125.7, 124.5, 123.1. LC-MS *m*/*z* 207.9 ([M + H]^+^, 100%).

**4-Nitroso-2-methylphenol (2-methyl-1,4-benzoquinone, E and Z isomers (9:4)) (3l)** [32]**:** Eluent: hexane-ethyl acetate (9:1) to yield a brown solid. Yield: 48%, 147 mg. LC-MS *m*/*z* 154.7 ([M + H]^+^, 100%), 152.4 ([M − H]^−^, 100). ^1^H NMR (400 MHz; CDCl_3_) 7.66 (1H, d, J 10.0, Aryl-*H*), 6.59 (1H, dd, J 10.0, 2.0, Aryl-*H*), 6.09 (1H, s, Aryl-*H*) 3.94 (3H, s, O-C*H*_3_). ^13^C NMR (100 MHz; CDCl_3_) 171.3 (*C*6), 170.1 (*C*1), 150.7 (*C*3), 135.3 (*C*4), 117.93 (*C*2), 100.56 (*C*5), 56.66 (*C*H_3_). HR-MS calculated for C_7_H_8_NO_3_ 154.0504, found: 154.0504. (0.0 ppm, 0.0 mDa).

**2-Chloro-4-nitrosophenol in equilibrium with 2-chloro-1,4-benzoquinone monoxime, E and Z isomers (4:3) (3m):** Eluent: hexane-ethyl acetate (4:1) to yield an orange solid. Yield: 34%, 289 mg. ^1^H NMR (400 MHz, Chloroform-*d*) δ 9.77 (s, 1H, O-*H*), 8.33 (d, *J* = 2.7 Hz, 1H, Aryl-*H*), 8.15 (dd, *J* = 9.0, 2.7 Hz, 1H), 8.01 (d, *J* = 2.4 Hz, 1H), 7.83 (dd, *J* = 10.2, 2.4 Hz, 1H), 7.48 (d, *J* = 2.5 Hz, 1H), 7.36–7.23 (m, 1H), 7.16 (d, *J* = 9.0 Hz, 1H), 6.67 (dd, *J* = 10.1, 3.8 Hz, 1H), 6.49 (s, 1H). ^13^C NMR (101 MHz, CDCl_3_) δ 180.46, 180.10, 156.96, 149.95, 149.25, 138.84, 137.69, 134.97, 134.65, 131.43, 129.78, 125.39, 124.63, 124.17, 122.05, 120.37, 116.30, 77.36, 77.04, 76.73. LC-MS: *m*/*z* = 158.0 ([M + H]^+^, 100%). HR-MS calculated for C_6_H_8_NO_2_^35^Cl 158.0009, found: 158.0024 (+9.5 ppm, +1.5 mDa).

**1,4-Benzoquinone monoxime (3o)** [33]**:** Isolated mixture, green solid. Yield: up to 55%, 135 mg. ^1^H NMR (400 MHz; CDCl_3_) 7.81 (1H, m, Aryl-*H*), 7.28 (1H, m, Aryl-*H*), 6.55 (2H, d, J 10.0, Aryl-*H*). ^13^C NMR (100 MHz; CDCl_3_) 160.5 (*C*1), 143.1 (*C*2), 136.0 (*C*5), 134.0 (*C*6), 129.2 (*C*4), 120.5 (*C*5). LC-MS: *m*/*z* 124.1 ([M + H]^+^, 100%). HR-MS calculated for C_6_H_6_NO_2_ 124.0401, found: 124.0399 (+1.6 ppm, +0.2 mDa). IR (neat) υ_max_/cm^−1^ 3316 (br, OH), 1553 (s, N=O), 1442 (s, N=O), 1330 (s, N=O), 1284 (s, N=O), 1127 (s, C-O).

**4-Nitrophenol (3o(i))** [34]**:** Brown solid. Yield: 55%, 154 mg. ^1^H NMR (400 MHz; d^6^-DMSO) 8.12 (2H, m, Aryl-*H*), 6.92 (2H, m, Aryl-*H*). ^13^C NMR (100 MHz; d^6^-DMSO) 164.6 (*C*), 139.9 (*C*1), 128.3 (*C*4) 126.7 (*C*3 and *C*5), 116.3 (*C*2 and *C*6). LC-MS *m*/*z* 138.1 ([M − H]^−^, 100%). IR (neat) υ_max_/cm^−1^ 3316 (br, OH), 1558 (s, N=O), 1496 (s, N=O), 1329 (s, N=O), 1284 (s, N=O), 1110 (s, C-O).

**Cycloadditions of copper(II) *bis*(2-nitrosophenolato)complexes.** As adapted from McKillop and Sayer [22], the copper complex (**2a–2h**) is purified using a Soxhlett procedure with hot ethanol-water, then the purified, solid material (1.25 mmol) dissolved in dimethoxyethane (40 mL) and water (5 mL). To this solution, dimethylacetylene dicarboxylate (5 mmol) in dimethoxyethane (2.5 mL) is added dropwise and the mixture stirred and heated (90 °C) under reflux for 4 h. After ambient cooling to room temperature, the remaining copper substances are removed by filtration through a bed of silica (2 cm depth) and then the filtrate is evaporated to dryness. The resulting solid is recrystallised into hexane-ethyl acetate (1:1) to give a crystalline solid (**4b, 4c, 4e, 4h**). **4b** was instead given using an improved procedure that used identical conditions but 0.25 mmol starting complex (**2b**) instead of 1.25 mmol and 1 mmol of DMAD instead of 5 mmol.

**Dimethyl 2-hydroxy-6-methyl-2H-benzo[b][1,4]oxazine-2,3-dicarboxylate (4a):** Recrystallised from hexane-ethyl acetate (1:1) to yield an orange solid. Yield: 59%, 154 mg. ^1^H NMR (400 MHz; d^6^-DMSO) 8.66 (1H, s, O*H*) 7.43 (1H, m, Aryl-*H*), 7.27 (1H, dd, J 8.0, 2.0 Aryl-*H*), 7.00 (1H, d, J 8.34, Aryl-*H*), 3.80 (6H, d, J 11.2, O-C*H*_3_), 2.32 (3H, s, O-C*H*_3_). ^13^C NMR (100 MHz; d^6^-DMSO) 167.4 (*C=O*), 162.3 (*C=O*), 148.9 (q-*C*), 142.5 (q-*C*), 132.6 (Aryl-*C*H), 130.8 (Aryl-*C*), 128.8 (Aryl-*C*H), 127.0 (Aryl-*C*), 119.0 (Aryl-*C*H), 89.7 (Aryl-*C*), 53.7 (O-*C*H_3_), 53.5 (O-*C*H_3_). LC-MS *m*/*z* 278.8 ([M − H]^−^, 100%), 280.8 ([M + H]^+^, 78). HR-MS calculated for C_13_H_14_NO_6_ 280.0816, found 280.0820 (−0.4 ppm, mDa −0.4). IR (neat) υ_max_/cm^−1^ 3164 (br, w, O-H), 2963 (w, C-H) 1764 (s, C=O), 1723 (s, C=O), 1439 (m, N-O), 1229 (s, N-O) 1129 (s, N-O), 1056 (s, C-O). *Crystal data:* CCDC 1957010, C_13_H_13_O_6_N, f.w. 279.24, T = 100 K, monoclinic, *a* = 5.861(6), *b* = 14.109(15), *c* = 7.528(8) Å, β = 90.70(3)°, *V* = 622.5(11) Å^3^, space group *Pc* (no. 7), *Z* = 2, pseudo-merohedral twinning by a 180° rotation around *z* axis, 3986 reflections (2028 unique, *R*_int_ = 0.111), *R*_1_ = 0.147, *wR*_2_ = 0.390. M.p = 207.2–207.8 °C.

**Dimethyl 6-chloro-2-hydroxy-2H-benzo[b] [1,4]oxazine-2,3-dicarboxylate (4b):** Recrystallised from hexane-ethyl acetate (1:1) to yield a yellow solid. Yield 19%, 142 mg. ^1^H NMR (400 MHz; CDCl_3_) 7.51 (1H, m, Aryl-*H*), 7.22 (1H, dd, J 8.0, 2.0, Aryl-*H*), 6.94 (1H, d, J 8.34, Aryl-*H*), 5.13 (1H, s, O-*H*), 3.97 (6H, d, J 11.2, O-C*H*_3_). ^13^C NMR (100 MHz; DMSO) 167.0 (*C=O*), 162.3 (*C=O*), 148.9 (q-*C*), 142.5 (q-*C*), 132.6 (Aryl-*C*H), 130.8 (Aryl-*C*), 128.8 (Aryl-*C*H), 127.0 (Aryl-*C*), 119.0 (Aryl-*C*H), 89.7 (Aryl-*C*), 53.7 (O-*C*H_3_), 53.5 (O-*C*H_3_). LC-MS *m*/*z* (300.3 [M + H]^+^, 100%), 302.3 ([M + H]^+^ (^37^Cl), 25). HR-MS calculated for C_12_H_11_NO_6_^35^Cl 300.0273 found: 300.0274 (−0.3 ppm, mDa −0.1). IR (neat) υ_max_/cm^−1^ 3189 (w, br, O-H), 2963 (w, C-H) 1768 (s, C=O), 1725 (s, C=O), 1439 (m, N-O), 1152 (s, N-O), 1000 (s, C-O). *Crystal data:* CCDC 1957004, C_12_H_10_O_6_NCl, f.w. 299.66, T = 120 K, monoclinic, *a* = 5.8201(12), *b* = 7.5030(15), *c* = 14.348(3) Å, β = 90.784(8)°, *V* = 626.5(2) Å^3^, space group *P*2_1_ (no. 4), *Z* = 2, pseudo-merohedral twinning by a 180° rotation around *x* axis, 7346 reflections (2831 unique, *R*_int_ = 0.043), *R*_1_ = 0.085, *wR*_2_ = 0.224. M.p = 217.4–219.0 °C.

**Dimethyl 6-bromo-2-hydroxy-2H-benzo[b] [1,4]oxazine-2,3-dicarboxylate (4c):** Recrystallised from hexane-ethyl acetate (1:1) to yield a cream solid. Yield 10%, 86 mg. ^1^H NMR (400 MHz; d^6^-DMSO) 8.79 (1H, s, O-*H*), 7.86 (1H, d, J 2.4, Aryl-*H*), 7.64 (1H, dd, J 8.8, 2.4 Aryl-*H*), 7.13 (1H, d, J 8.8, Aryl-*H*), 3.86 (3H, s, J 11.2, O-C*H*_3_), 3.77 (3H, s, O-C*H*_3_). ^13^C NMR (100 MHz; d^6^-DMSO) 167.0 (*C=O*), 162.3 (*C=O*), 148.9 (q-*C*), 142.9 (q-*C*), 135.4 (Aryl-*C*H), 131.7 (Aryl-*C-*H), 131.2 (Aryl-*C*), 119.5 (Aryl-*C-*H), 114.5 (Aryl-*C*), 89.7 (Aryl-*C*), 53.8 (O-*C*H_3_), 53.6 (O-*C*H_3_). LC-MS: *m*/*z* 346.3 ([M + H]^+^ (^81^Br), 52%), 344.6 ([M − H]^−^ (^81^Br), 100). HR-MS calculated for C_12_H_11_NO_6_^79^Br 343.9770, found 343.9757. (−3.8 ppm, mDa −1.3). IR (neat) υ_max_/cm^−1^ 3189 (w, br, O-H), 2963 (w, C-H) 1768 (s, C=O), 1725 (s, C=O), 1439 (m, N-O), 1152 (s, N-O), 1000 (s, C-O). M.p, = 220.9–222.7 °C.

**Dimethyl 6-formyl-2-hydroxy-8-methoxy-2H-benzo[b] [1,4]oxazine-2,3-dicarboxylate (4d):** Recrystallised from hexane-ethyl acetate (7:3) to yield a cream solid. Yield 45%, 190 mg. ^1^H NMR (400 MHz, DMSO-d6) δ 9.96 (s, 1H, ROC-*H*), 9.19 (s, 1H, O-*H*), 7.86 (d, J = 1.8 Hz, 1H, Aryl-*H*), 7.61 (d, J = 1.8 Hz, 1H, Aryl-*H*), 3.93 (s, 3H, O-C*H*_3_), 3.87 (s, 3H, O-C*H*_3_), 3.79 (s, 3H, O-C*H*_3_). ^13^C NMR (101 MHz, DMSO-d_6_) δ 191.68 (*C*HO), 166.76 (C=O), 162.31 (C=O), 149.1, 148.5, 138.4, 131.2 (2C), 130.1, 125.2 (Aryl-*C*H), 112.8 (Aryl-*C*H), 56.6 (O-*C*H_3_), 53.9 (O-*C*H_3_), 53.7 (O-*C*H_3_). LC-MS: *m*/*z* 324.4 ([M + H]^+^, 100%), 322.2 ([M − H]^−^, 100). HR-MS calculated for C_14_H_11_NO_6_^79^Br 322.0563, found 322.0569. (+1.9 ppm, mDa +0.6). IR (neat) υ_max_/cm^−1^ 2964 (w, C-H), 1765 (s, C=O), 1724 (s, C=O), 1601 (w, N-H), 1485 (m), 1284 (s) 1132 (s, C-O), 975 (s), 799 (s) 584 (m). *Crystal data*: CCDC 1963155, C_14_H_13_O_8_N, f.w. 323.25, T = 100 K, monoclinic, *a* = 5.8339(14), *b* = 10.095(3), *c* = 12.653(3) Å, α = 103.773(5), β = 91.131(5), γ = 96.467(5)°, *V* = 718.4(3) Å^3^, space group *P*$\bar{1}$ (no. 2), *Z* = 2, 6906 reflections (2896 unique, *R*_int_ = 0.057), *R*_1_ = 0.064, *wR*_2_ = 0.195. M.p, = 212.0–213.8 °C.

**Dimethyl 7-methoxy-2-hydroxy-2H-benzo[b][1,4]oxazine-2,3-dicarboxylate (4i):** Recrystallised from hexane-ethyl acetate (3:2) to yield a white solid. Yield 30%, 109 mg. ^1^H NMR (400 MHz, DMSO) δ 8.68 (s, 1H, O-*H*), 7.53 (s, 1H, Ar-*H*), 6.76 (Aryl-*H*), 6.69 (Aryl-*H*), 3.81 (s, 3H, O-C*H*_3_), 3.76 (s, 3H, O-C*H*_3_), 3.74(s, 3H, O-C*H*_3_). ^13^C NMR (101 MHz, DMSO-d_6_) δ 167.45, 163.26, 162.84, 145.06, 144.12, 130.91, 123.95, 110.41, 101.87, 89.72, 56.38, 53.57, 53.15. IR (neat) υ_max_/cm^−1^ 2970 (w, C-H), 1761 (s, C=O), 1719 (s, C=O), 1596 (w, N-H), 1438 (m), 1273 (s) 1159 (s, C-O), 1110 (s), 822 (s) 457 (m). LC-MS: *m*/*z* 296.3 ([M + H]^+^, 100%), 294.1 ([M − H]^−^, 100). HR-MS calculated for C_13_H_14_NO_7_ 296.0770, found: 296.0790 (+6.8 ppm, +2.0 mDa). M.p, = 217.4–219.1 °C.

**Dimethyl 2-hydroxy-7-methyl-2H-benzo[b][1,4]oxazine-2,3-dicarboxylate (4k):** Recrystallised from hexane-ethyl acetate (1:1) to yield a yellow solid. Yield 9%, 62.8 mg. ^1^H NMR (400 MHz; d^6^-DMSO) 8.66 (1H, s, O-*H*) 7.43 (1H, m, Aryl-*H*), 7.27 (1H, dd, J 8.0, 2.0 Aryl-*H*), 7.00 (1H, d, J 8.34, Aryl-*H*), 3.80 (6H, d, J 11.2, O-C*H*_3_), 2.32 (3H, s, O-C*H*_3_). ^13^C NMR (100 MHz; d^6^-DMSO) 167.4 (*C=O*), 162.7 (*C=O*), 146.4 (q-*C*), 143.9 (q-*C*), 143.3 (Aryl-*C*), 129.4 (Aryl-*C*H), 127.8 (Aryl-*C*), 124.4 (Aryl-*C*H), 117.4 (Aryl-*C*H), 89.7 (Aryl-*C*), 53.7 (O-*C*H_3_), 53.5 (O-*C*H_3_), 21.7 (*C*H_3_). LC-MS: *m*/*z* 278.8 ([M − H]^−^, 100%) 280.8 ([M + ]^+^, 100). HR-MS calculated for C_13_H_14_NO_6_ 280.0824, found: 280.0820. (−0.4 ppm, −0.4 mDa). IR (neat) υ_max_/cm^−1^ 3164 (w, br, O-H), 2963 (w, C-H) 1764 (s, C=O), 1723 (s, C=O), 1439 (m, N-O), 1229 (s, N-O) 1129 (s, N-O), 1056 (s, C-O). M.p. = 211.8–213.4 °C.

### 3.3. X-ray Crystallography

X-ray diffraction experiments for **2a**, **2d**, **2k**, β-**3j** and **4b** and were carried out on a Bruker 3-circle D8 Venture diffractometer with a PHOTON 100 CMOS area detector, using Mo-*Kα* or (for **2k**) Cu-*Kα* radiation from Incoatec IμS microsources with focusing mirrors (λ = 0.71073 and 1.54184 Å, respectively). Crystals were cooled to 120 K using a Cryostream (Oxford Cryosystems) open-flow N_2_ gas cryostat. The data were processed using APEX3 v.2016.1-0 and reflection intensities integrated using SAINT v8.38A software (Bruker AXS, 2016). **2c**, α-**3j, 4a** and **4d** were studied at Beamline I19 of Diamond Light Source (RAL) on a dual air-bearing fixed-χ diffractometer with pixel-array photon-counting Dectris Pilatus 2M detector [35], using undulator radiation monochromated with double-crystal Si(111) (λ = 0.6889 Å, for **4d**, 0.9098 Å). The crystal was cryo-mounted using remote-controlled BART robot [35] and cooled to 100 K using a Cryostream cryostat. The diffraction images were converted to Bruker format using cbf_to_sfrm.py program [35] and further processed with APEX3 and SAINT software (vide supra). The data were corrected for absorption by semi-empirical method based on Laue equivalents and multiple scans using SADABS 2016/2 program (2014/4 for **2a**) [36]. The data from twinned crystals of **4a** and **4b** were deconvoluted using PLATON TwinRotMat program [37]. The structures **2a** and **2k** were solved by direct methods using SHELXS 2013/1 software [38], the rest by dual-space intrinsic phasing method using SHELXT 2018/2 program [39], and refined by full-matrix least squares using SHELXL 2018/3 software [40] on OLEX2 platform [41].

## 4. Conclusions

Copper *bis*(2-nitrosophenolato) complexes, including several new structures, have been generated in fair-to-high yields using a simple copper-mediated nitrosation procedure, starting from sufficiently reactive phenolic substrates. The target complex was generated when the substituents of the substrate encourage *ortho*-nitrosation or discourage *para*-nitrosation, with a different type of copper compound being formed from other substrates. This confirms that the latter substrates cannot form 2-nitrosophenolato complexes in this way and instead the more complicated ‘Baudisch reaction’ [9] conditions must be used; hence addressing some confusion across the existing literature. Where possible, the organic ligands, or their products of oxidation, have been isolated from the complexes and understanding of the stability of certain 2-nitrosophenol compounds has improved. The complexes have undergone cycloadditions in low-to-high yields to a unique bicyclic structure that is different to the literature proposition and the scope includes two new compounds.

## Figures and Tables

**Table 1:** Copper-mediated nitrosation of substituted phenols.

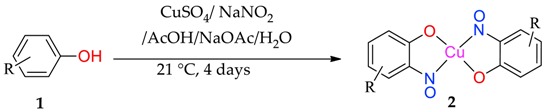
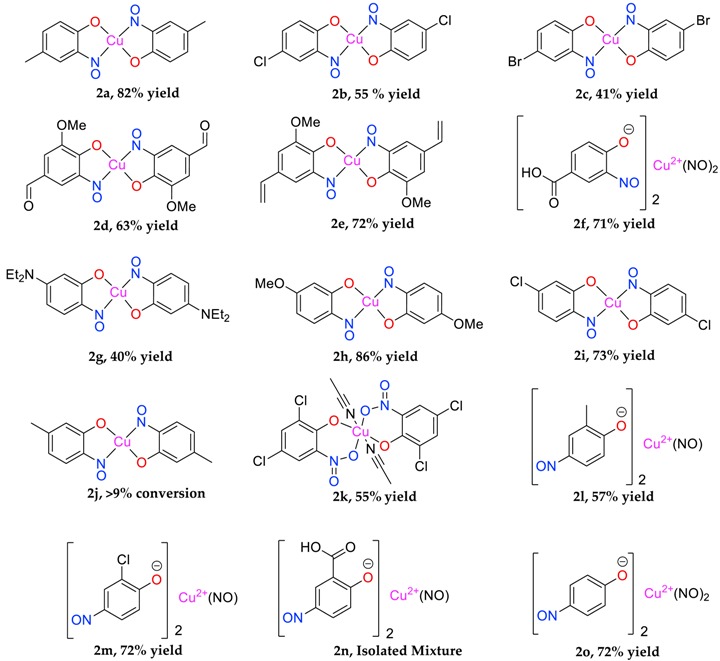

Yields refer to pure isolated, anhydrous compounds. **2n** could not be separated from some inorganic impurities, hence a pure isolated yield cannot be given. **2k** was obtained after recrystallisation from acetonitrile-chloroform. The structures shown for **2f**, **2l**, **2m**, **2n** and **2o** are a best estimate based on the data collected (see Section 3, Materials and Methods).

**Table 2:** Organic ligand isolation from copper(nitrosophenolato) compounds.

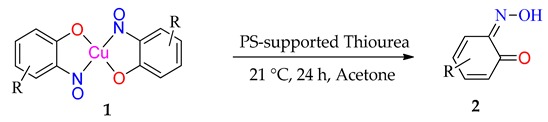
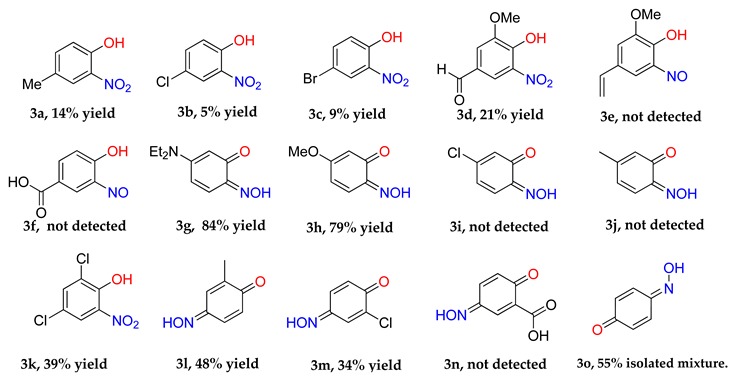

Yields refer to pure isolated compounds.

**Table 3:** The categorisation of nitrosophenol compounds identified in this study according to their stability.

Stable	Oxidises in Air	Decomposes or Oxidises
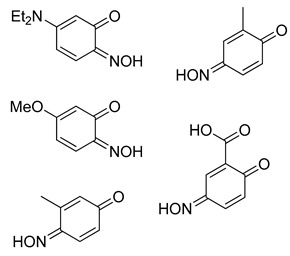	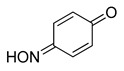	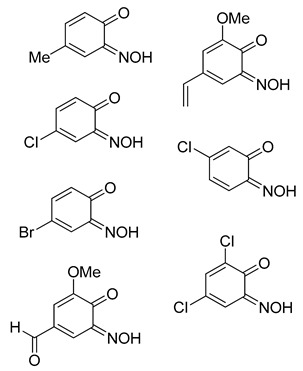

‘Stable’ compounds did not deteriorate noticeably under aerobic, room-temperature conditions. One compound alternatively ‘oxidises in air’ to a stable product. Finally, several compounds ‘decompose or oxidise’, forming a stable nitro-derivative alongside a complex mixture.

**Table 4:** 2,4-Cycloaddition reactions of copper-nitrosophenolato compounds to unique bicyclic structures.

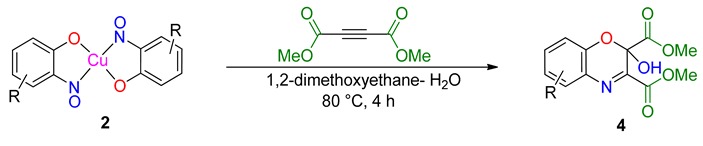
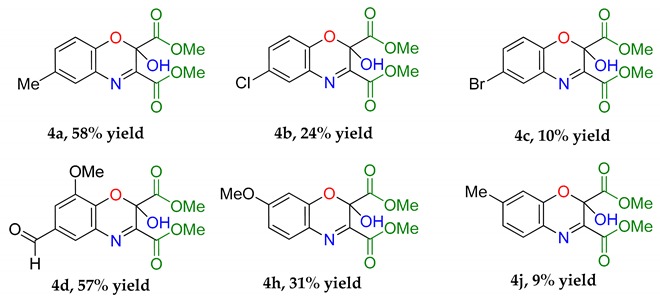

Yields refer to pure isolated compounds.

## Supplementary Materials

The following are available online, ^1^H and ^13^C-NMR spectra, mass spectra, infrared spectra and XRD crystallographic data of compounds. Crystallographic data in CIF format have been deposited with Cambridge Structural Database (CCDC-1957003 to 1957010, 1963155) and are available online.
