# Supplementary material for: Copper-Mediated Nitrosation: 2-Nitrosophenolato Complexes and Their Use in the Synthesis of Heterocycles
Source: Molecules. 2019 Nov 16;24(22):4154. doi: 10.3390/molecules24224154 (PMC6891769; doi:10.3390/molecules24224154)
Supplement: Supplementary file 1 [file molecules-24-04154-s001.pdf]

Supplementary Information

Towards Ortho-Nitrosation of Phenols via Formation of Copper-Nitrosophenolato Complexes- Supplementary Information

Alexander J. Nicholls[1], Andrei S. Batsanov[1] and Ian R. Baxendale[1*]

[1] Department of Chemistry, University of Durham, South Road, Durham, Durham, UK. DH1 3LE.

S2 – X-ray Crystallography for compounds **2a,c,d,k**, **3j(i)**, **4a,b, d**

S6 – NMR spectra of compounds **3a-3o** and **4a-4h**.

S27 – Accurate mass measurements for compounds **2a, b, d, h, i, 3a-c, g, h, j(i), l, m, o, 4a-4h**.

S38 – IR spectra of compounds **2a-2o**, **3j(i)**, **l**, **4a-4h**.

X-ray crystallography

Table S1. Crystal data and experimental details

**Compound**	**2a**	**2c**	**2d**	**2k**	**α-3j**	**β-3j**	**4a**	**4b**	**4d**
	15srv268	19srv011	19srv247	19srv134	19srv010	19srv133	19srv012	19srv228	19srv290
CCDC	1957008	1957005	1957006	1957007	1957003	1957009	1957010	1957004	1963155
Formula	$C_{16}H_{18}CuN_2O_5$	$C_{14}H_{12}Br_2CuN_2O_5$	$C_{16}H_{12}CuN_2O_8$	$C_{16}H_{10}Cl_4CuN_4O_6$	$C_7H_7NO_2$	$C_7H_7NO_2$	$C_{13}H_{13}NO_6$	$C_{12}H_{10}ClNO_6$	$C_{14}H_{13}NO_8$
$D_{calc.}$/ g cm^{-3}	1.598	2.070	1.835	1.810	1.365	1.389	1.490	1.589	1.494
μ/mm^{-1}	1.40	5.74	1.48	6.70	0.10	0.10	0.11	0.33	0.24
Formula Weight	381.86	511.62	423.82	559.62	137.14	137.14	279.24	299.66	323.25
T/K	120	100	120	120	100	120	100	120	100
Crystal System	monoclinic	monoclinic	monoclinic	orthorhombic	triclinic	monoclinic	monoclinic	monoclinic	triclinic
Space Group	$P2_1/c$ (no.14)	$P2_1/n$ (no.14)	$P2_1/c$ (no.14)	*Cmce* (no.64)	*P*-1 (no.2)	$P2_1/c$ (no.14)	*Pc* (no.7)	$P2_1$ (no.4)	*P*-1 (no.2)
a/Å	10.1875(8)	7.0128(16)	6.4741(5)	20.491(2)	7.2648(8)	12.8007(13)	5.861(6)	5.8201(12)	5.8339(14)
b/Å	6.9915(5)	10.020(2)	12.0560(9)	7.5407(7)	7.3201(8)	7.7538(8)	14.109(15)	7.5030(15)	10.095(3)
c/Å	22.2994(17)	23.311(5)	10.1090(7)	13.2872(14)	12.8572(14)	13.8767(14)	7.528(8)	14.348(3)	12.653(3)
α/°	90	90	90	90	88.333(2)	90	90	90	103.773(5)
β/°	91.781(3)	93.502(5)	103.595(3)	90	78.713(2)	107.805(4)	90.70(3)	90.784(8)	91.131(5)
γ/°	90	90	90	90	84.279(2)	90	90	90	96.467(5)
V/Å^3	1587.5(2)	1635.0(6)	766.92(10)	2053.1(4)	667.14(13)	1311.4(2)	622.5(11)	626.5(2)	718.4(3)
Z	4	4	2	4	4	8	2	2	2
λ/Å	0.71073	0.6889	0.71073	1.54184	0.6889	0.71073	0.6889	0.71073	0.9098
Radiation	MoK_α	synchrotron	MoK_α	CuK_α	synchrotron	MoK_α	synchrotron	MoK_α	synchrotron
$2\Theta_{max}$/°	56	55	60	133.2	58.4	55	49.6	55	70
Reflections total	22237	19075	16527	8029	9388	16949	3986	7346	6906
unique	3840	4121	2239	922	3893	3000	2028	2831	2896
with $I > 2\sigma(I)$	3047	3493	1712	672	2964	2356	844	2495	2211
R_{int}	0.050	0.081	0.047	0.073	0.050	0.036	0.111	0.043	0.057
Parameters	221	221	126	132	237	237	186	188*	230
Restraints	5	3	0	111	0	0	158	154	0
$\Delta\rho_{max,min}$/eÅ^{-3}	0.48, -0.36	2.42, -0.96	0.65, -0.49	0.37, -0.35	0.63, -0.33	0.28, -0.18	0.85, -0.48	2.55, -0.81	0.43, -0.36
Goodness of fit	1.037	1.056	1.037	1.079	1.073	1.039	1.076	1.061	1.125
wR_2 (all data)	0.081	0.240	0.101	0.118	0.159	0.102	0.390	0.224	0.195
wR_2 [$I > 2\sigma(I)$]	0.075	0.235	0.094	0.108	0.154	0.095	0.338	0.214	0.182
R_1 (all data)	0.054	0.093	0.059	0.069	0.068	0.051	0.250	0.094	0.079
R_1 [$I > 2\sigma(I)$]	0.034	0.084	0.037	0.045	0.056	0.037	0.147	0.085	0.064

*Flack parameter x= –0.06(5)

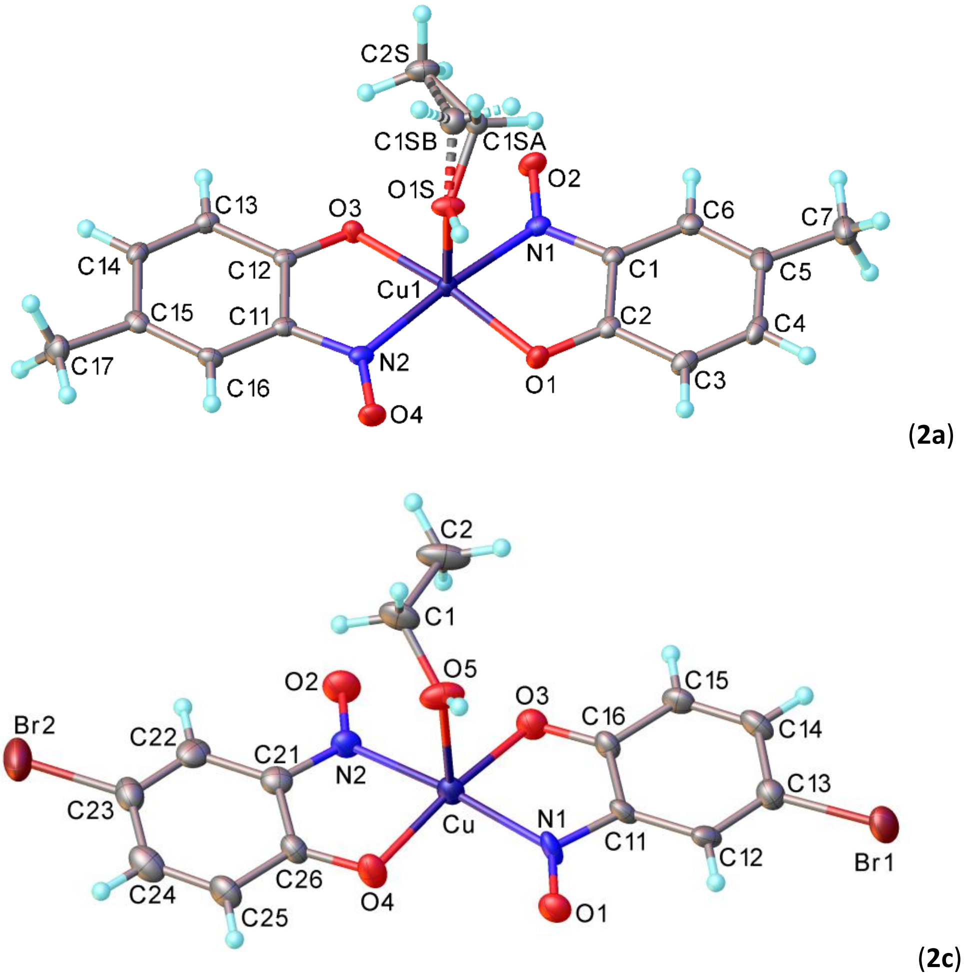

Figure S1. X-ray molecular structures of **2a** (showing the disorder) and **2c.** Thermal ellipsoids are drawn at the 50% probability level.

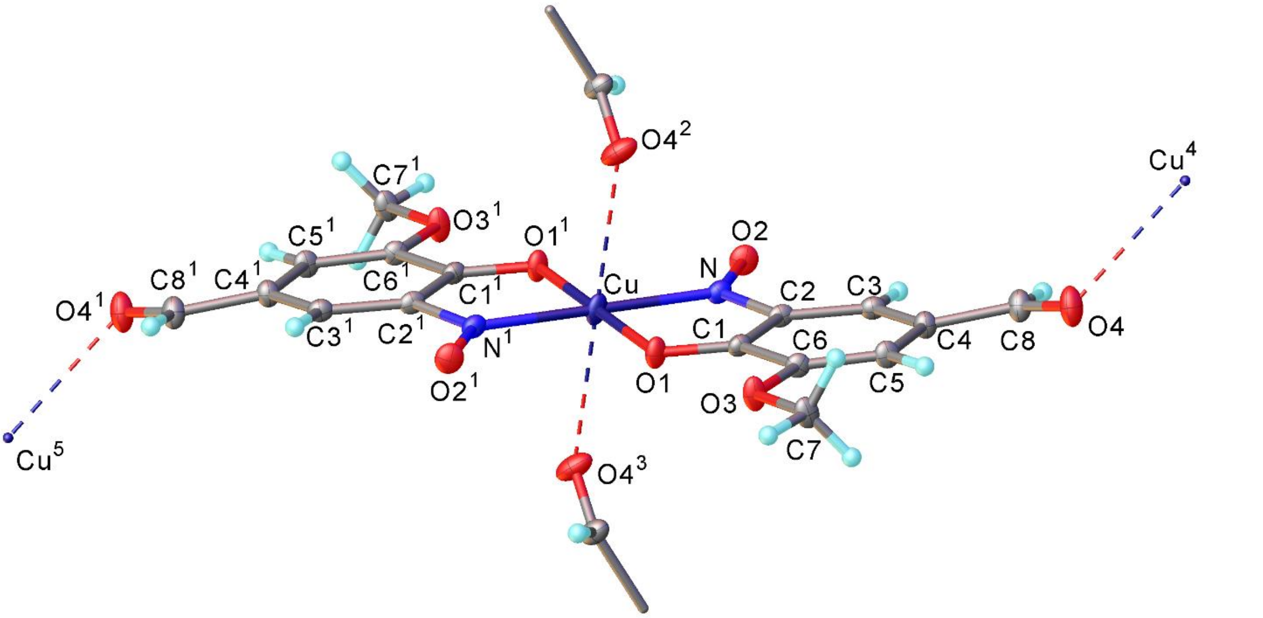

Figure S2. X-ray molecular structure of **2d**, showing intermolecular interactions (dashed lines). Symmetry transformations: (1) –*x*, 1–*y*, –*z*; (2) *x*–1, 3/2–*y*, *z*–½; (3) 1–*x*, *y*–½, ½–*z*; (4) *x*+1, 3/2–*y*, *z*+½; (5) –1–*x*, *y*–½, –½–*z*.

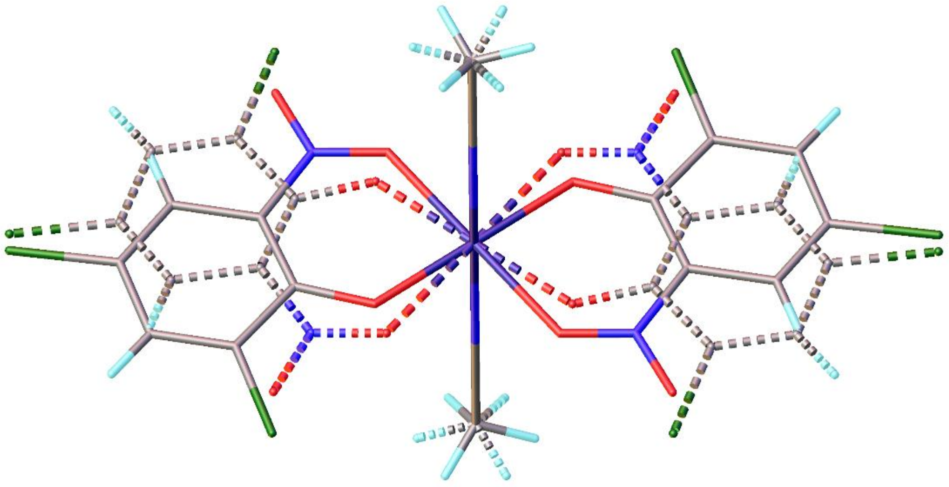

Figure S3. Disorder of the molecule **2k** in crystal.

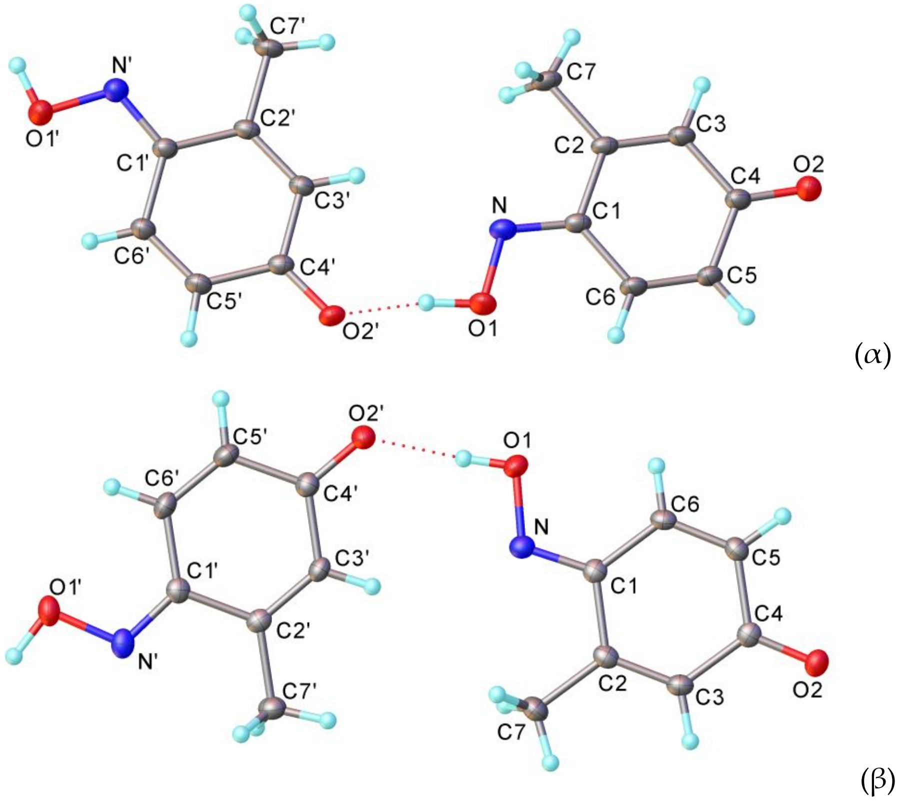

Figure S4. Independent molecules in the triclinic (α) and monoclinic (β) polymorphs of **3j**

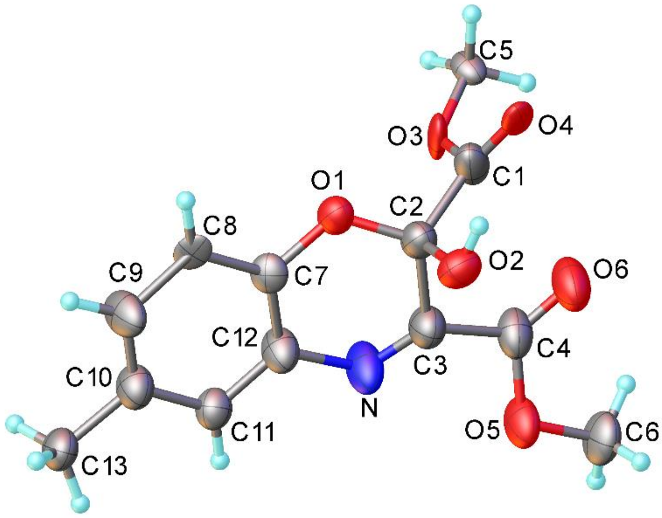

(**4a**)

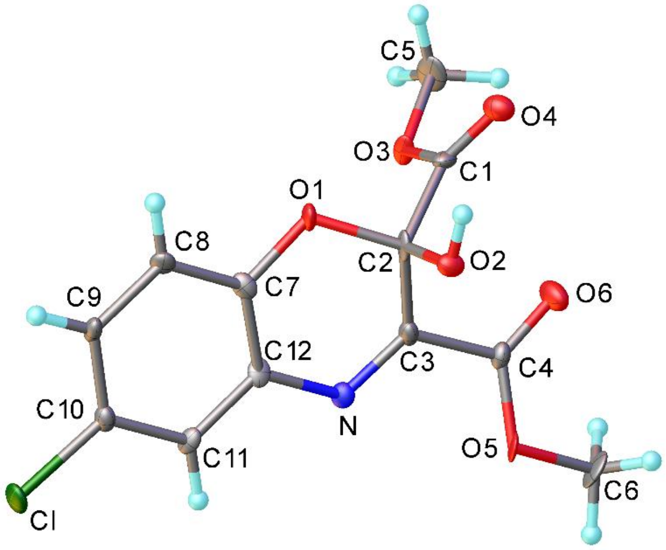

(**4b**)

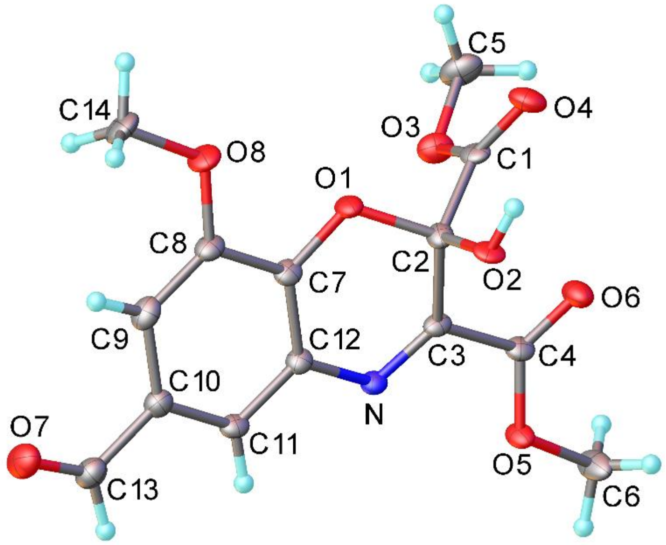

(**4d**)

Figure S5. X-ray molecular structures of **4a, 4b** and **4d**

NMR Spectra of Selected Compounds

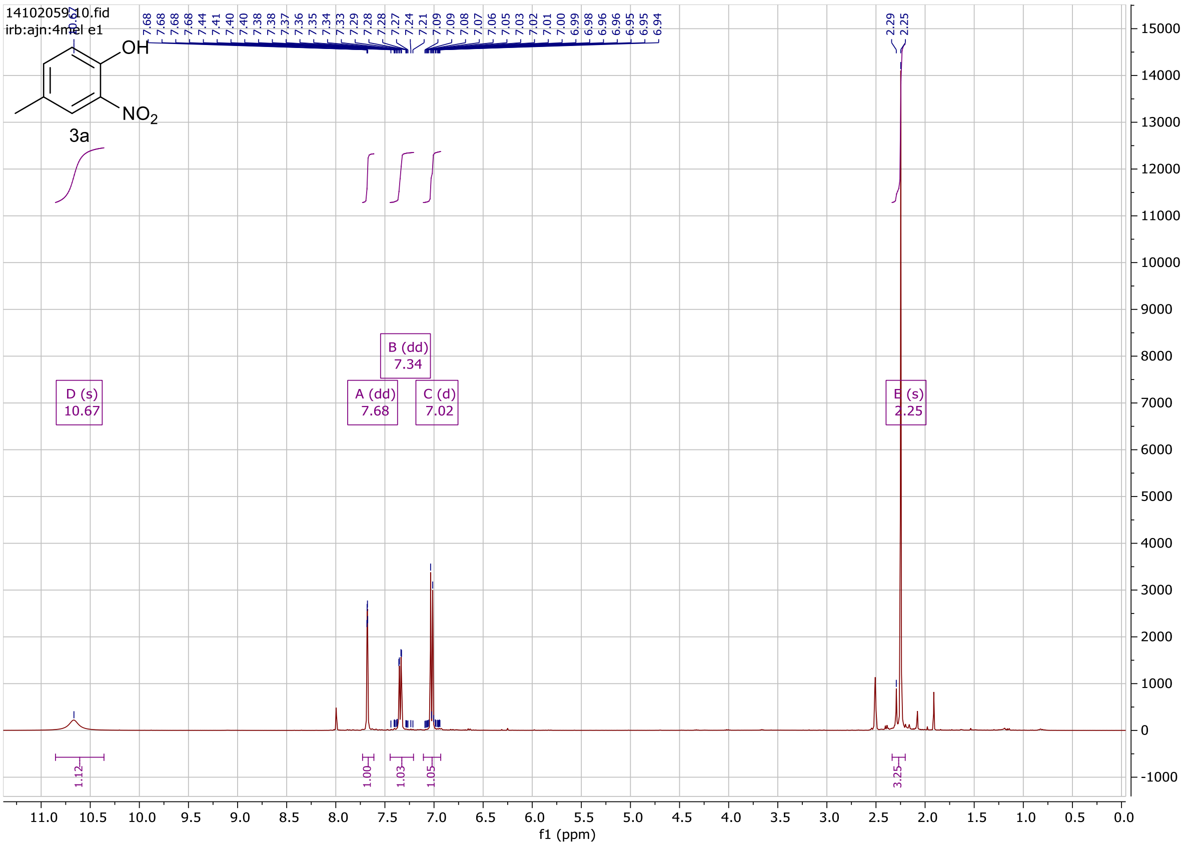

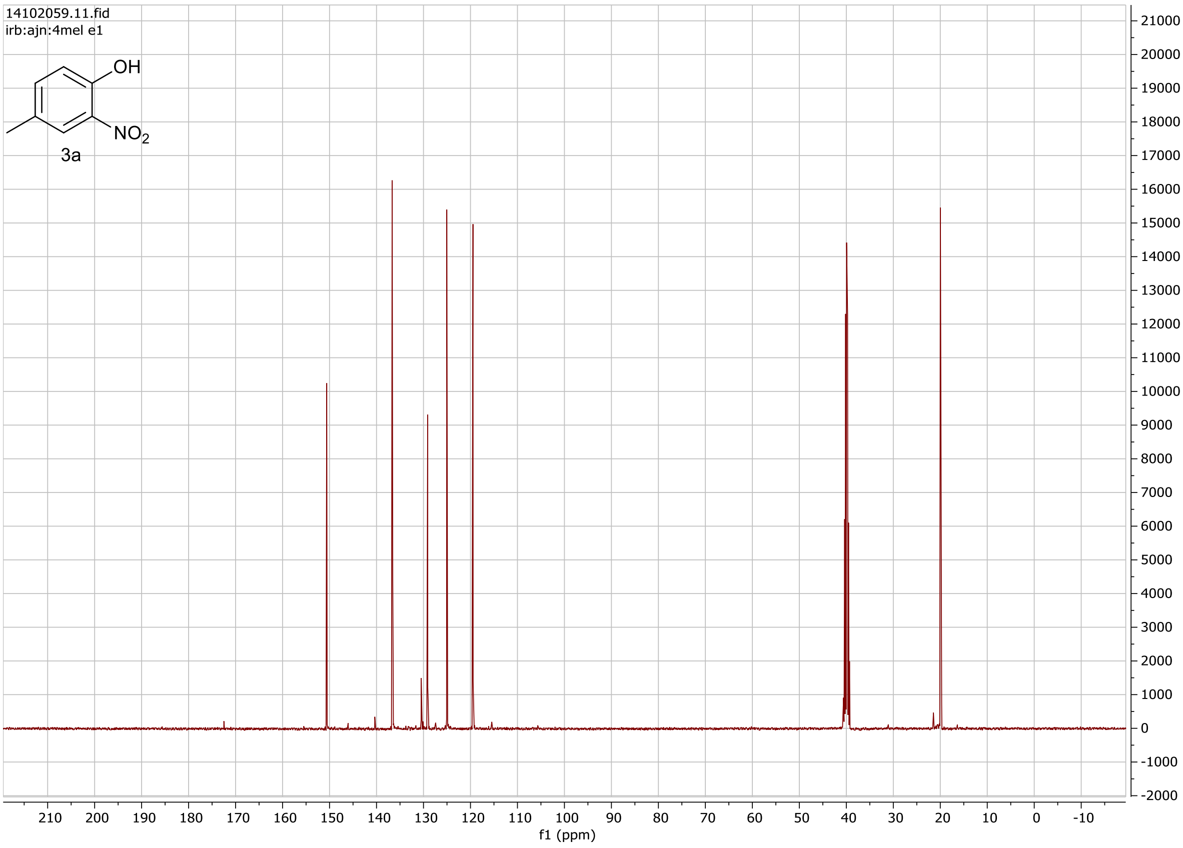

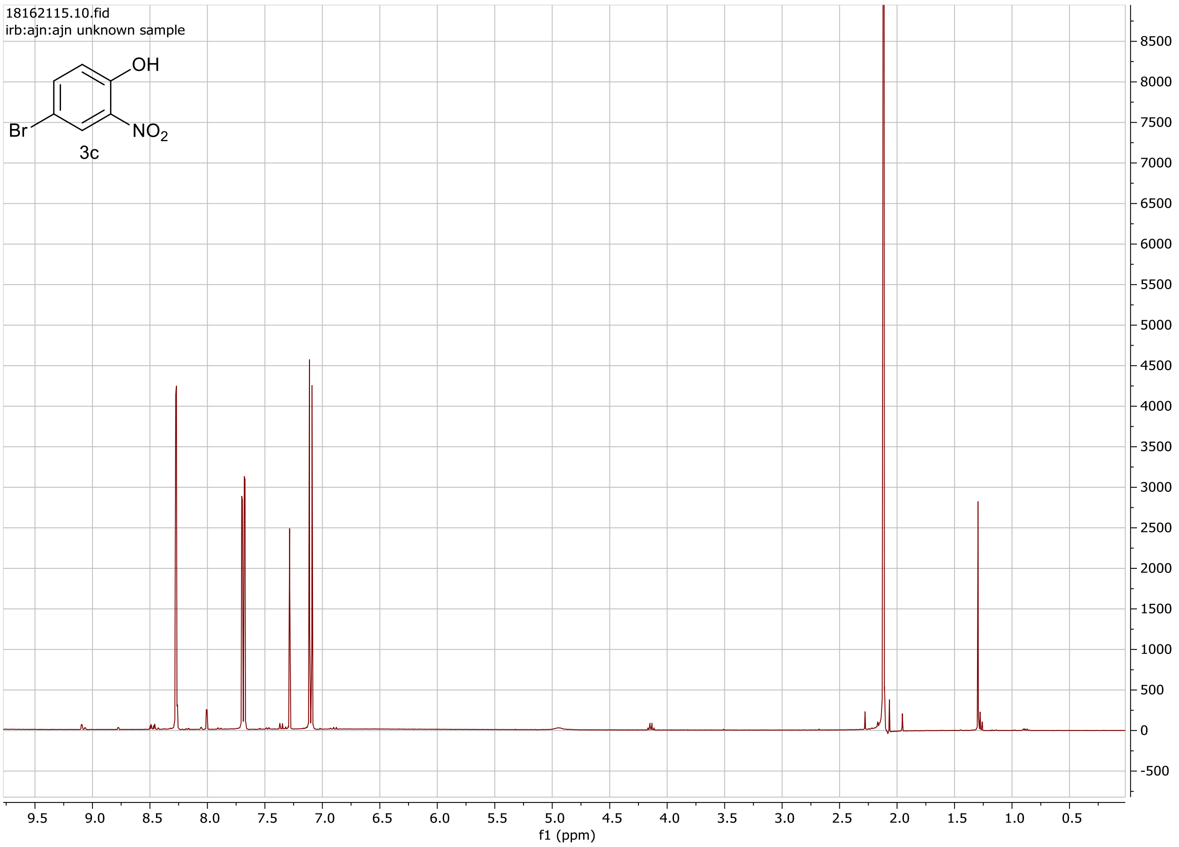
18162115.10.fid
irb:ajn:ajn unknown sample
OH
Br
NO2
3c
f1 (ppm)

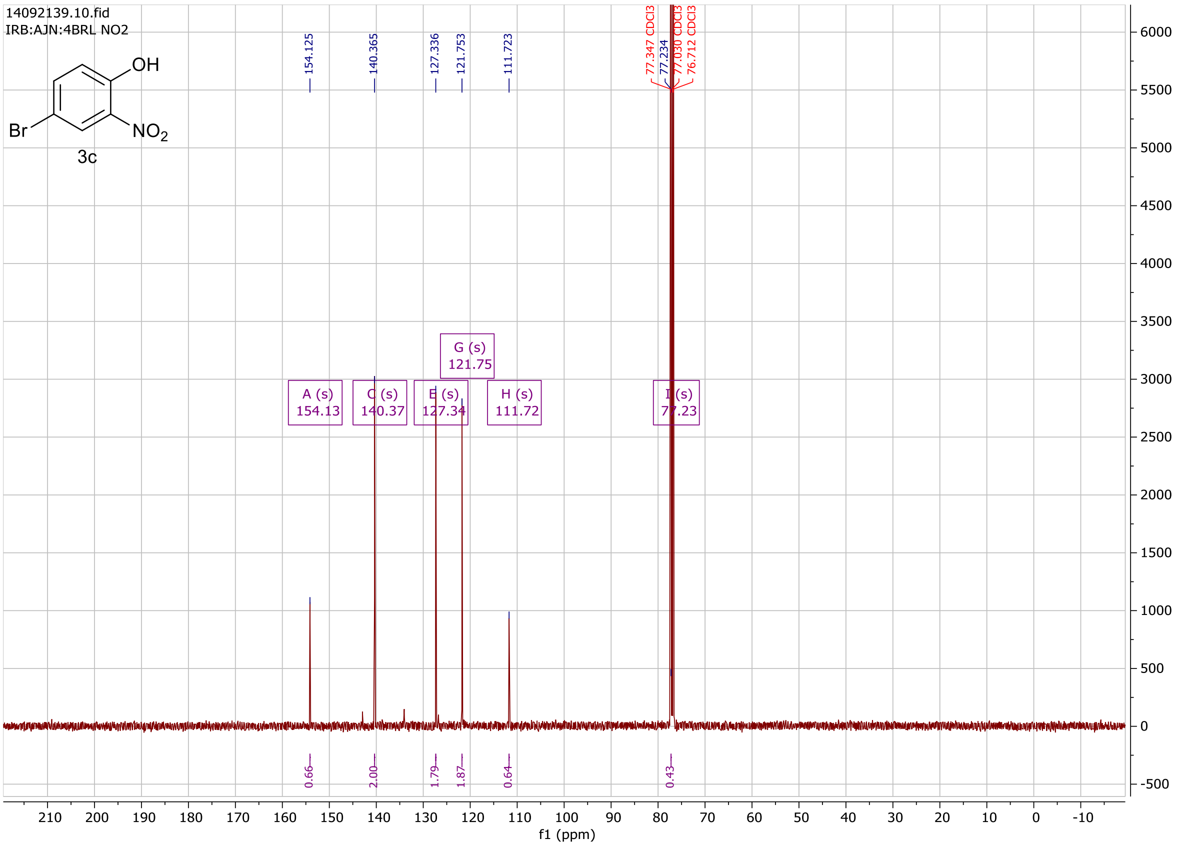
14092139.10.fid
IRB:AJN:4BRL NO2
OH
Br
NO2
3c
154.125
140.365
127.336
121.753
111.723
77.347 CDCl3
77.234
77.030 CDCl3
76.712 CDCl3
A (s) 154.13
C (s) 140.37
E (s) 127.34
G (s) 121.75
H (s) 111.72
I (s) 77.23
0.66
2.00
1.79
1.87
0.64
0.43
f1 (ppm)

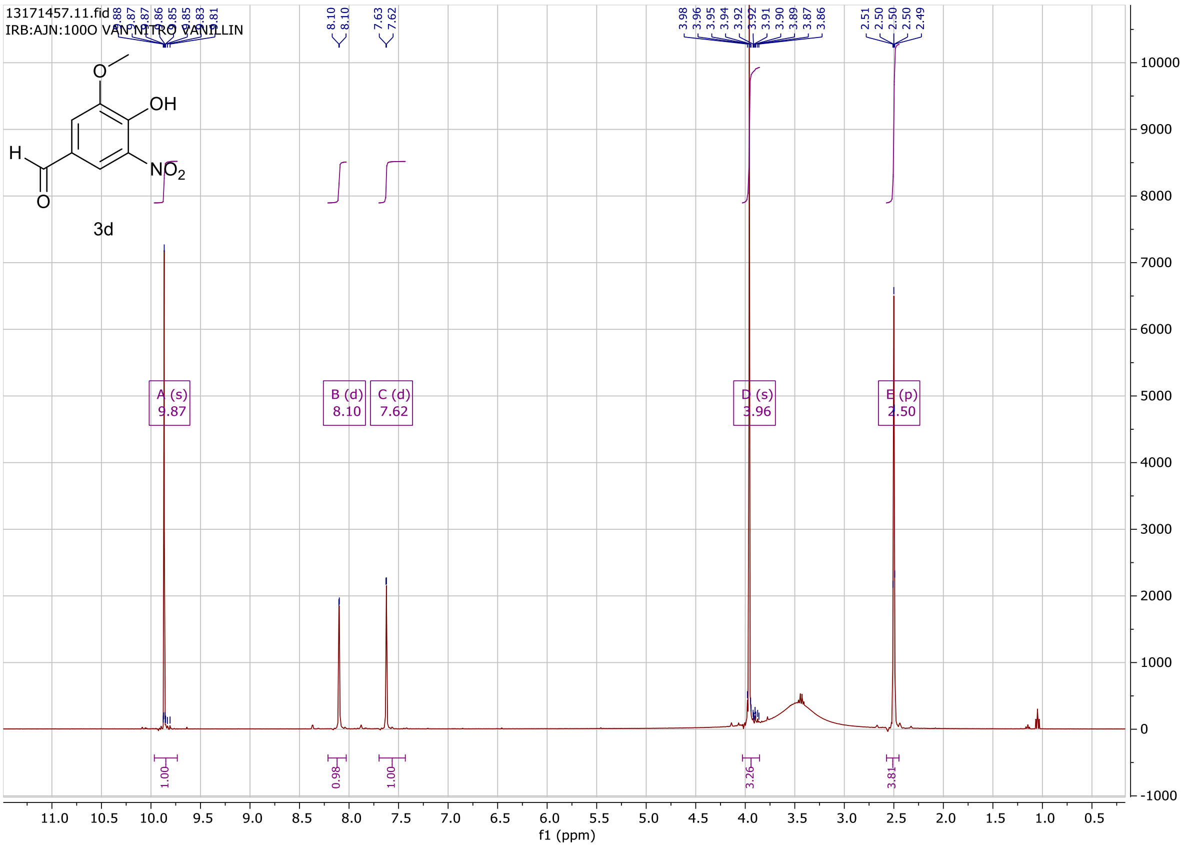
13171457.11.fid
IRB:AJN:100O VAN NITRO VANILLIN
OH
NO_2
O
H
3d
A (s) 9.87
B (d) 8.10
C (d) 7.62
D (s) 3.96
E (p) 2.50
f1 (ppm)

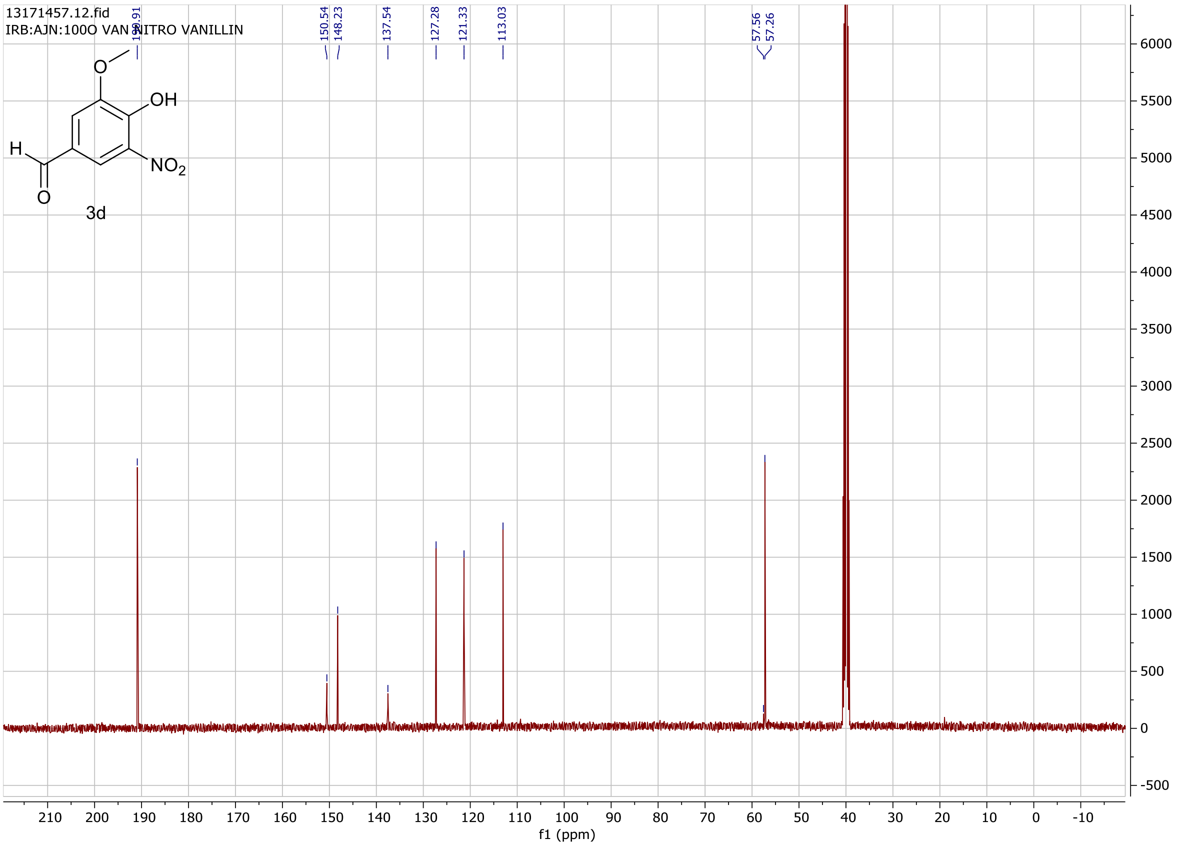
13171457.12.fid
IRB:AJN:100O VAN NITRO VANILLIN
150.54
148.23
137.54
127.28
121.33
113.03
57.56
57.26
OH
NO_2
O
H
3d
f1 (ppm)

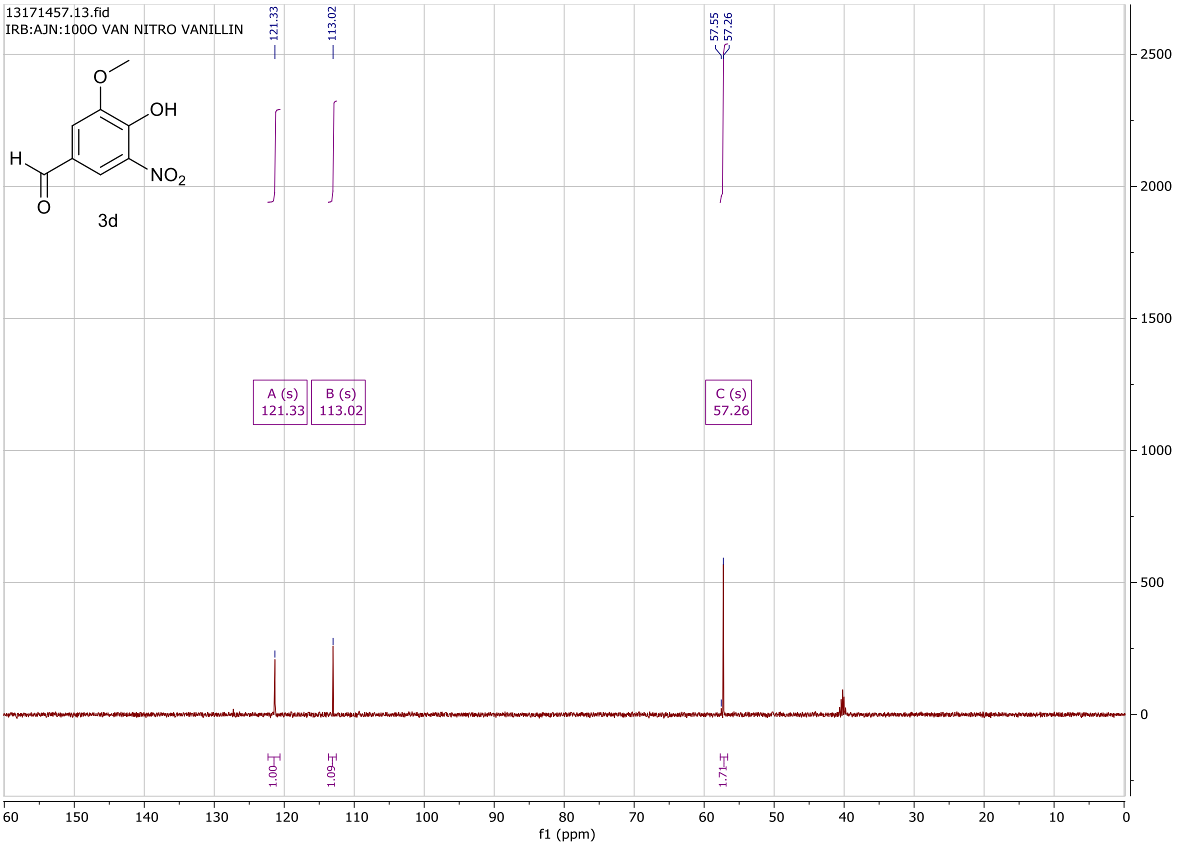
13171457.13.fid
IRB:AJN:100O VAN NITRO VANILLIN
OH
NO2
H
O
3d
121.33
113.02
57.55
57.26
A (s)
121.33
B (s)
113.02
C (s)
57.26
1.00
1.09
1.71
f1 (ppm)

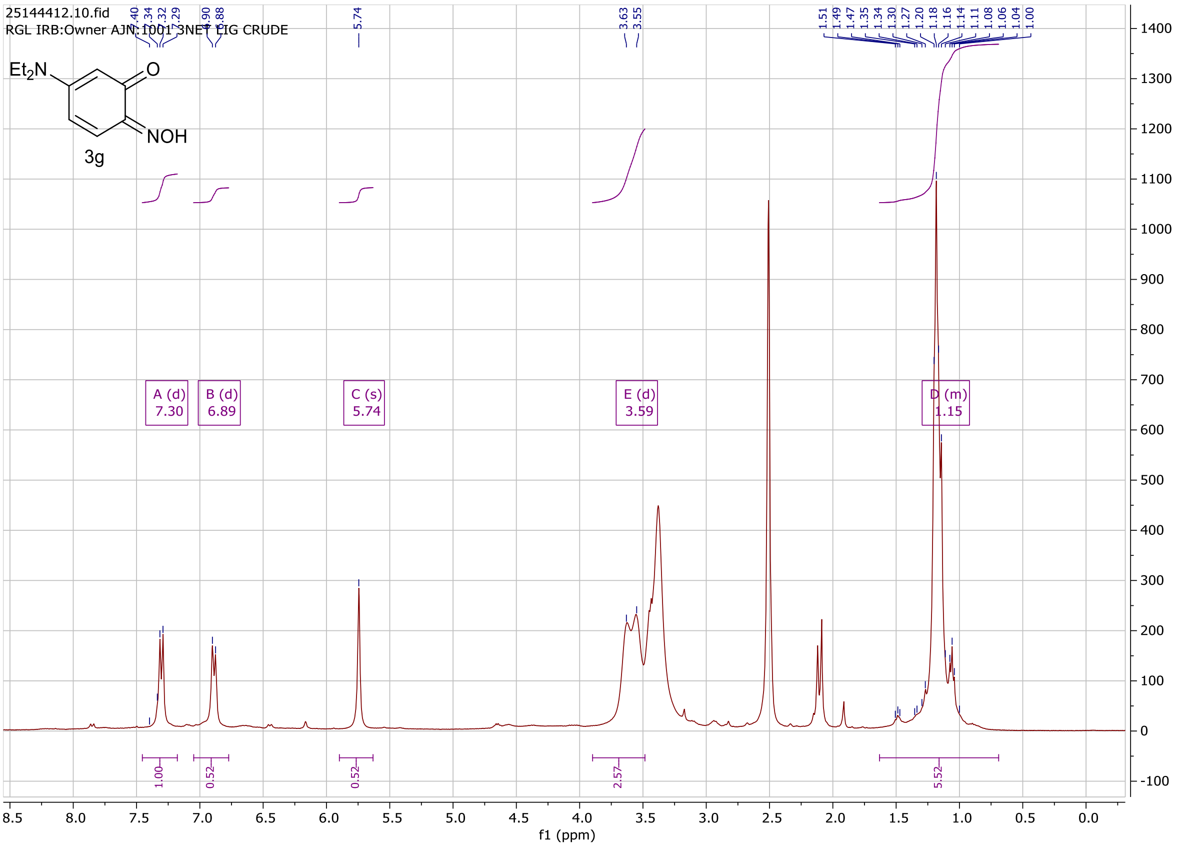
25144412.10.fid
RGL IRB:Owner AJN:I001 3NET EIG CRUDE
Et2N
O
NOH
3g
5.74
3.63
3.55
A (d)
7.30
B (d)
6.89
C (s)
5.74
E (d)
3.59
D (m)
1.15
1.00
0.52
0.52
2.57
5.52
f1 (ppm)

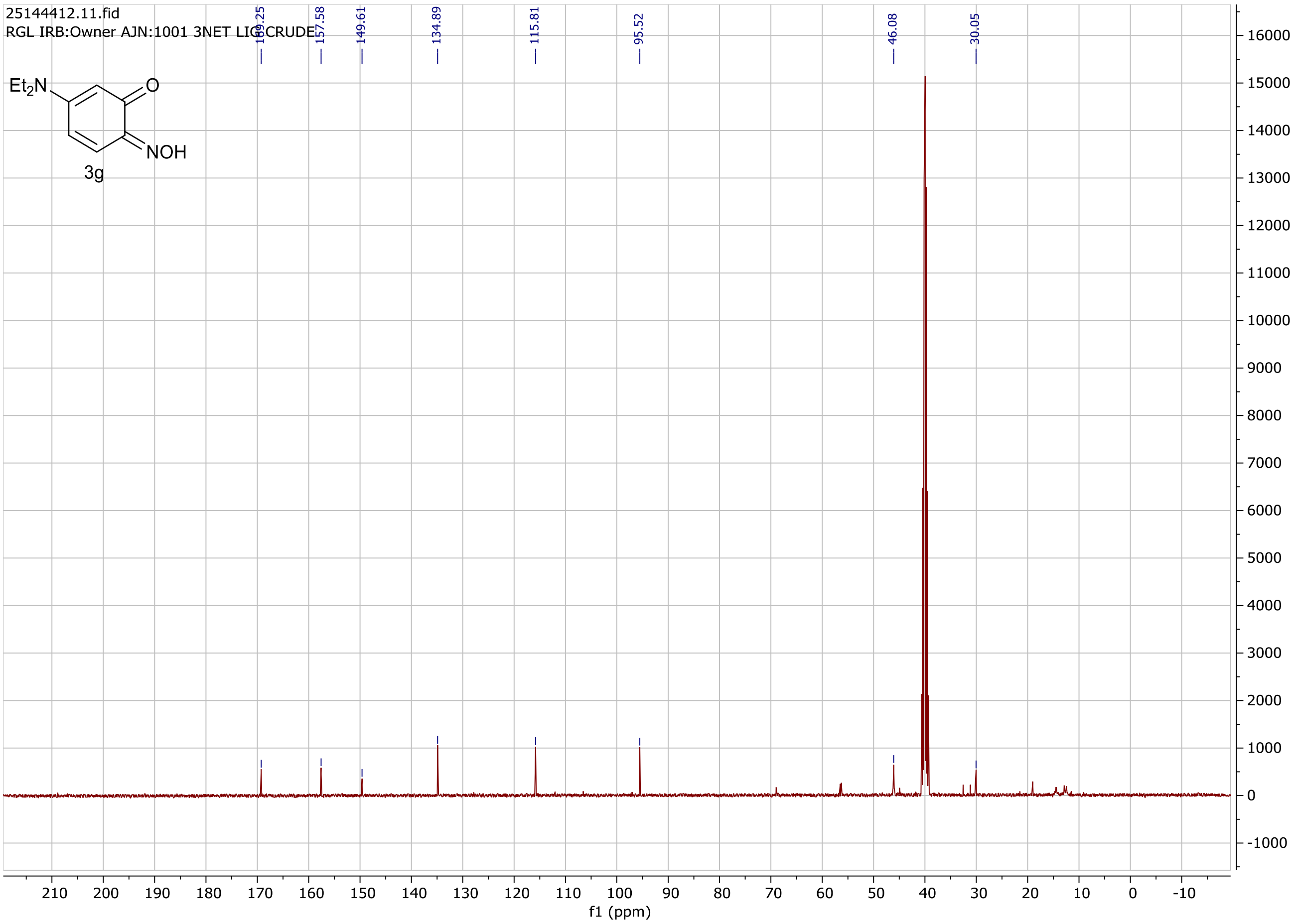
25144412.11.fid
RGL IRB:Owner AJN:1001 3NET LIG CRUDE
169.25
157.58
149.61
134.89
115.81
95.52
46.08
30.05
Et2N
O
NOH
3g
f1 (ppm)

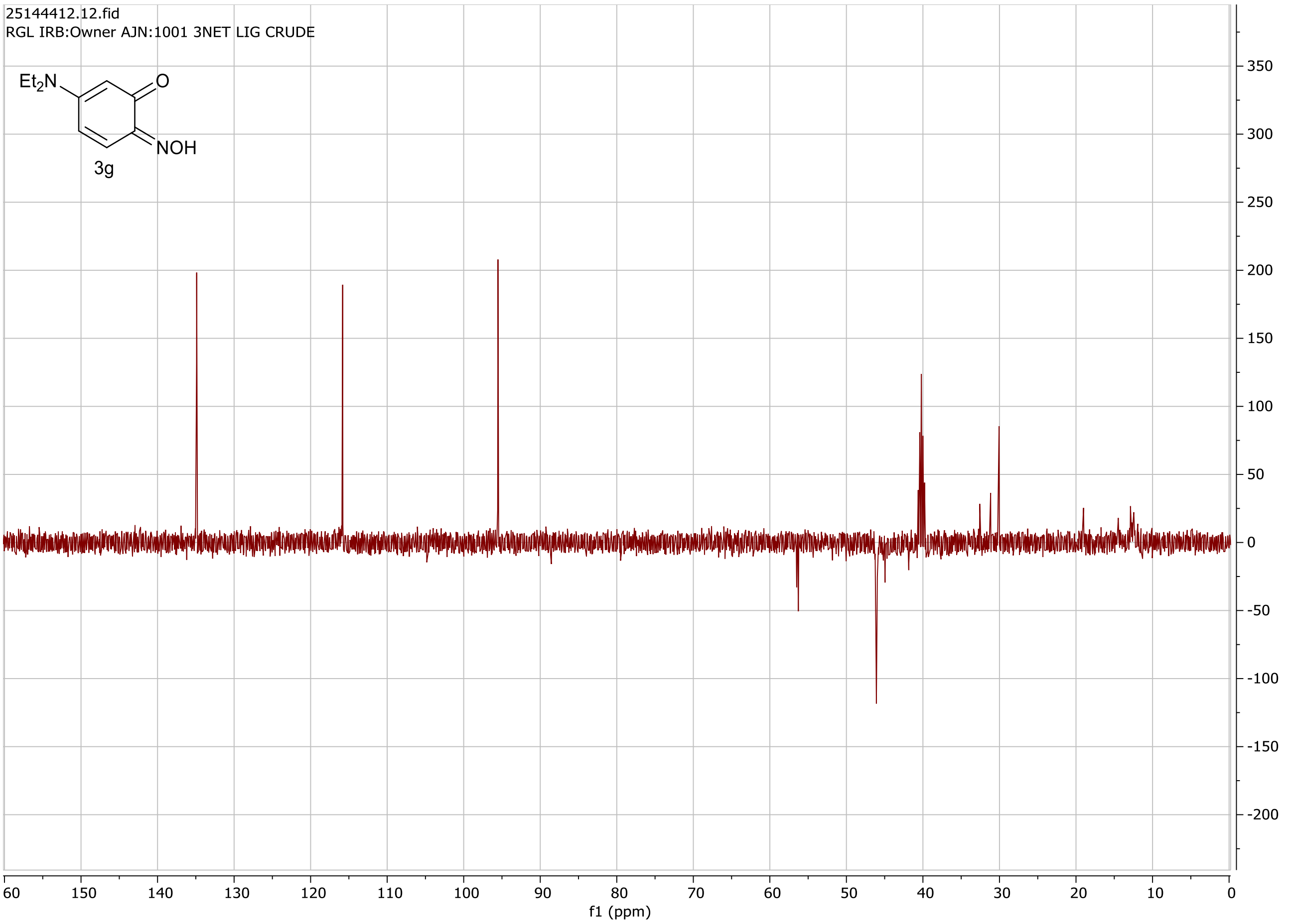
25144412.12.fid
RGL IRB:Owner AJN:1001 3NET LIG CRUDE
Et2N
O
NOH
3g
f1 (ppm)

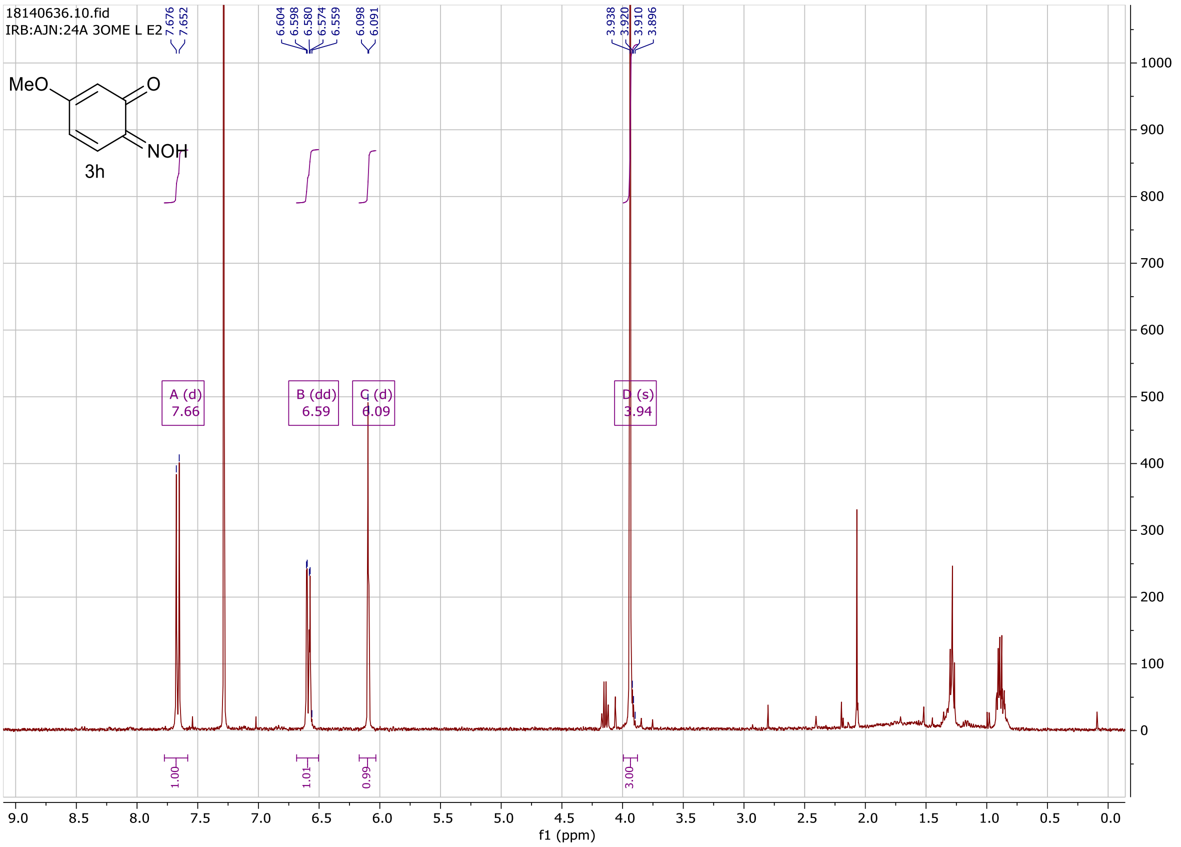
18140636.10.fid
IRB:AJN:24A 3OME L E2
7.676
7.652
6.604
6.598
6.580
6.574
6.559
6.098
6.091
3.938
3.920
3.910
3.896
MeO
O
NOH
3h
A (d)
7.66
B (dd)
6.59
C (d)
6.09
D (s)
3.94
1.00
1.01
0.99
3.00
f1 (ppm)

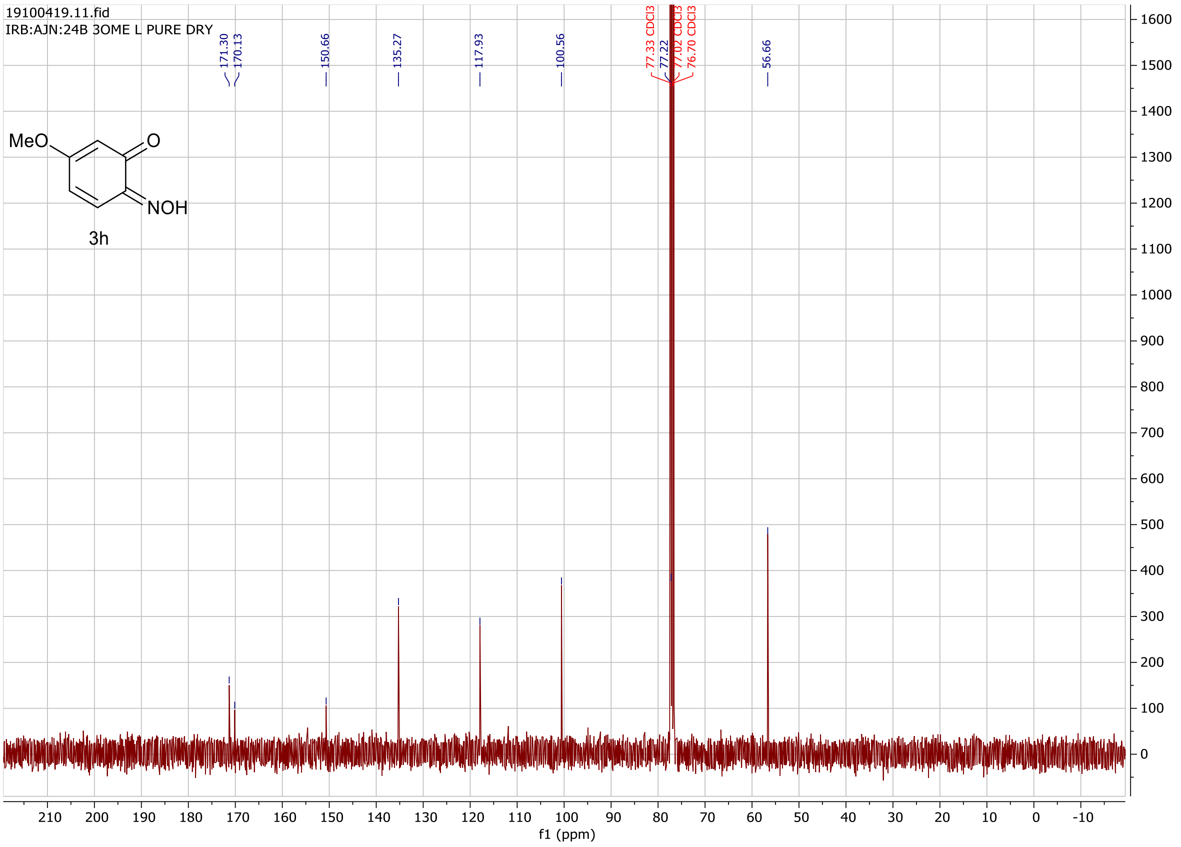
19100419.11.fid
IRB:AJN:24B 3OME L PURE DRY
171.30
170.13
150.66
135.27
117.93
100.56
77.33 CDCl3
77.22
77.02 CDCl3
76.70 CDCl3
56.66
MeO
O
NOH
3h
f1 (ppm)

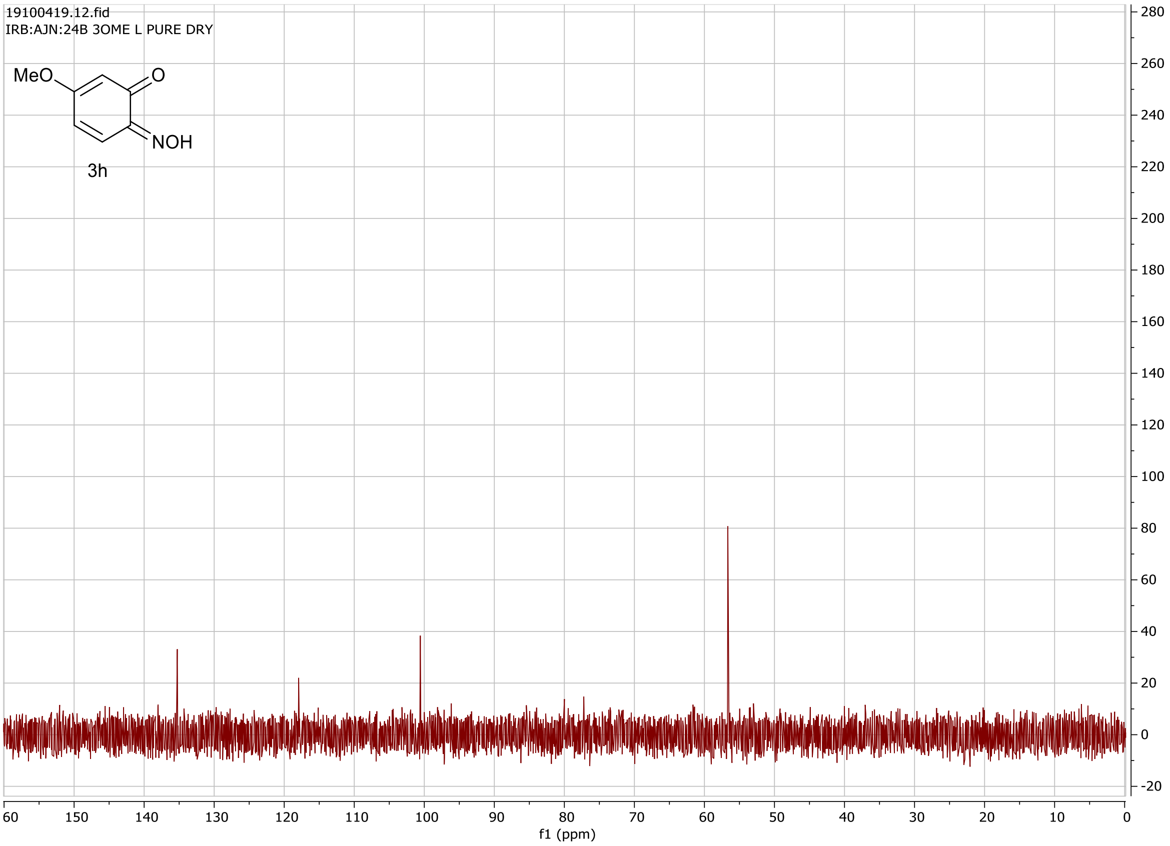
19100419.12.fid
IRB:AJN:24B 3OME L PURE DRY
MeO
O
NOH
3h
f1 (ppm)

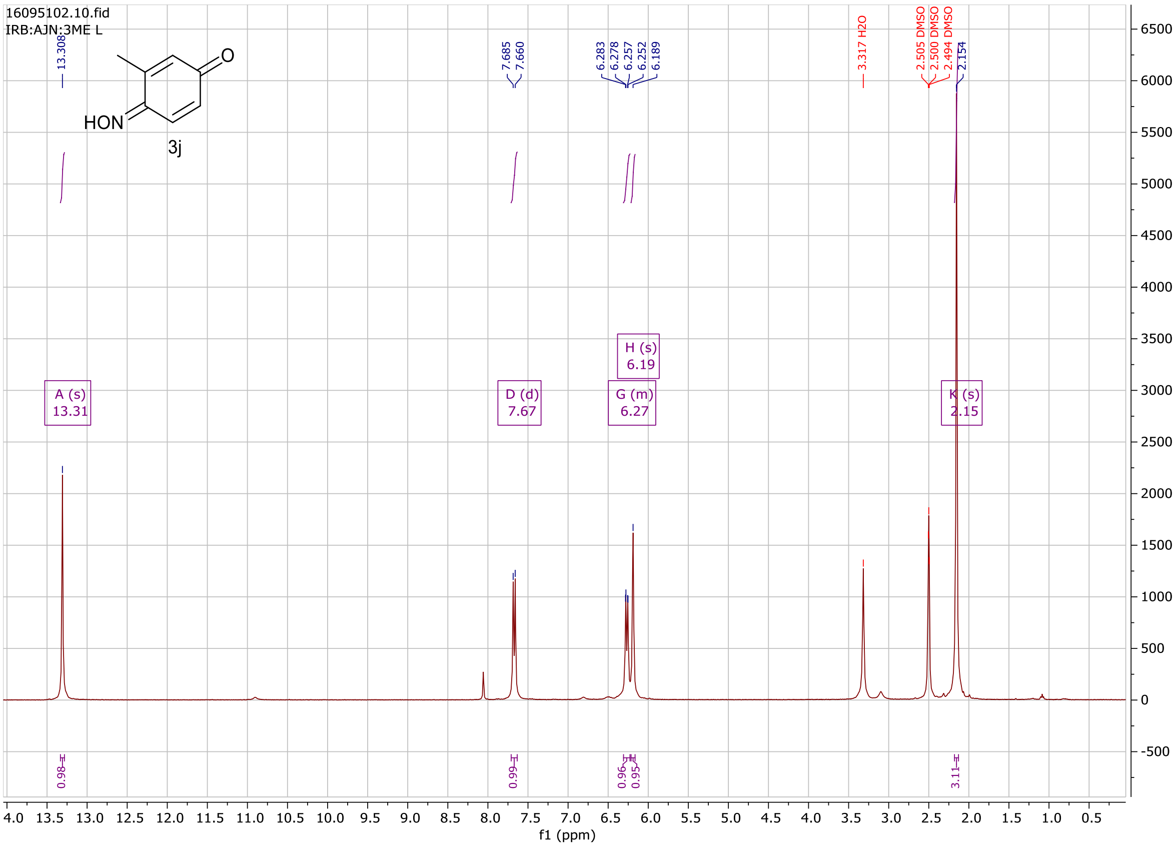
16095102.10.fid
IRB:AJN:3ME L
13.308
7.685
7.660
6.283
6.278
6.257
6.252
6.189
3.317 H2O
2.505 DMSO
2.500 DMSO
2.494 DMSO
2.154
O
HON
3j
A (s)
13.31
D (d)
7.67
H (s)
6.19
G (m)
6.27
K (s)
2.15
0.98
0.99
0.96
0.95
3.11
f1 (ppm)

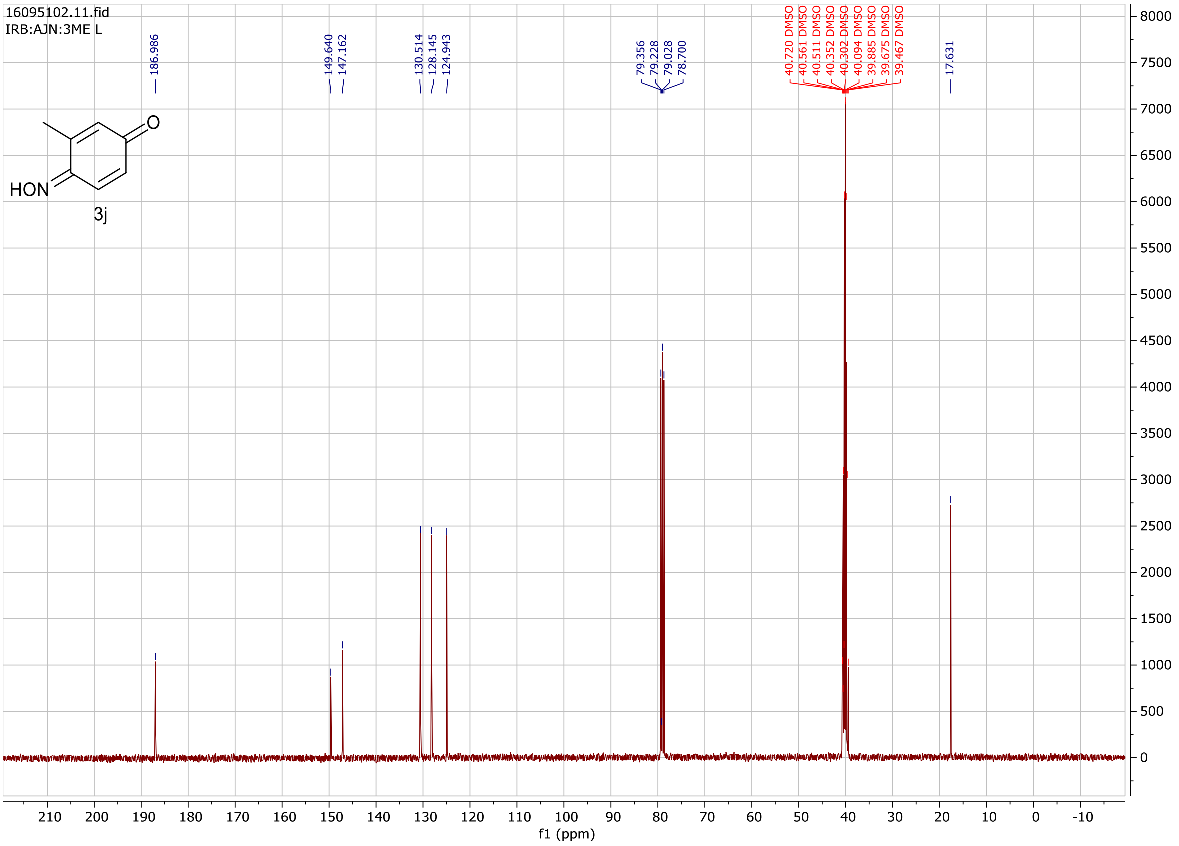
16095102.11.fid
IRB:AJN:3ME L
186.986
149.640
147.162
130.514
128.145
124.943
79.356
79.228
79.028
78.700
40.720 DMSO
40.561 DMSO
40.511 DMSO
40.352 DMSO
40.302 DMSO
40.094 DMSO
39.885 DMSO
39.675 DMSO
39.467 DMSO
17.631
HON
O
3j
f1 (ppm)

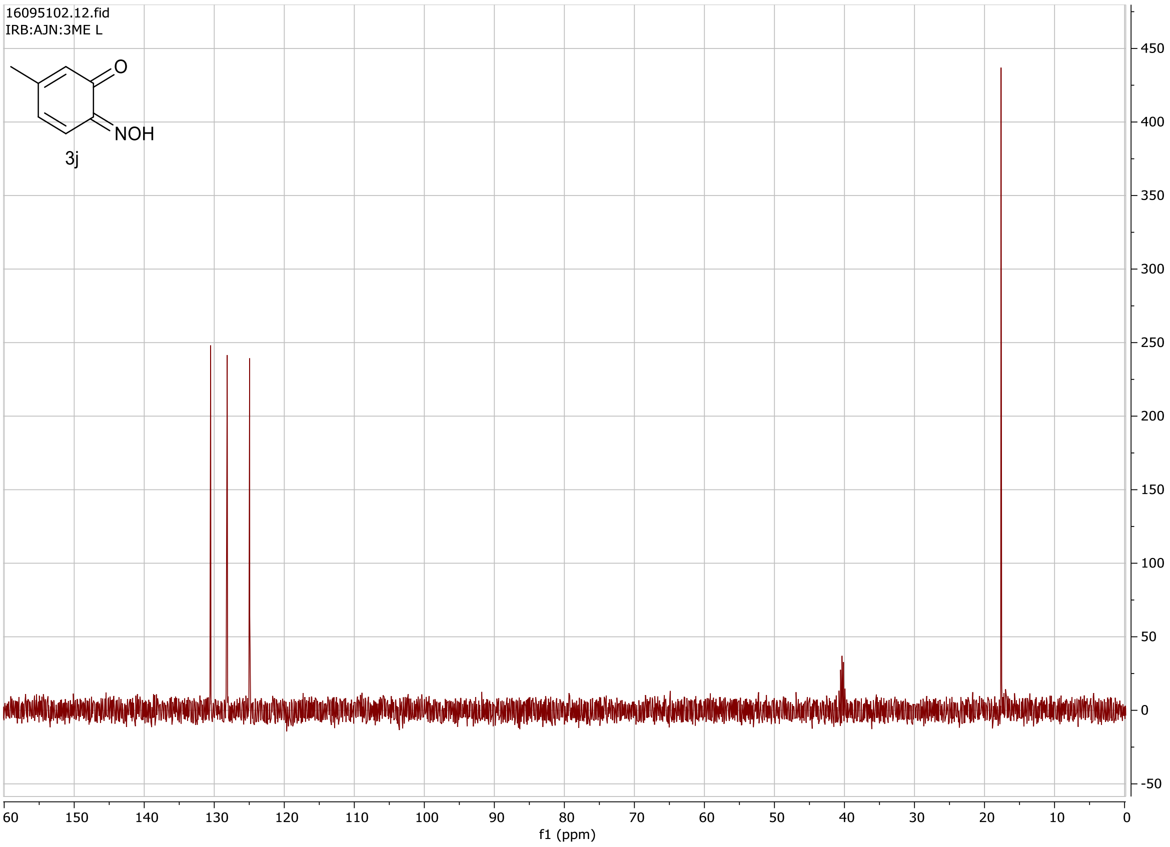
16095102.12.fid
IRB:AJN:3ME L
O
NOH
3j
f1 (ppm)

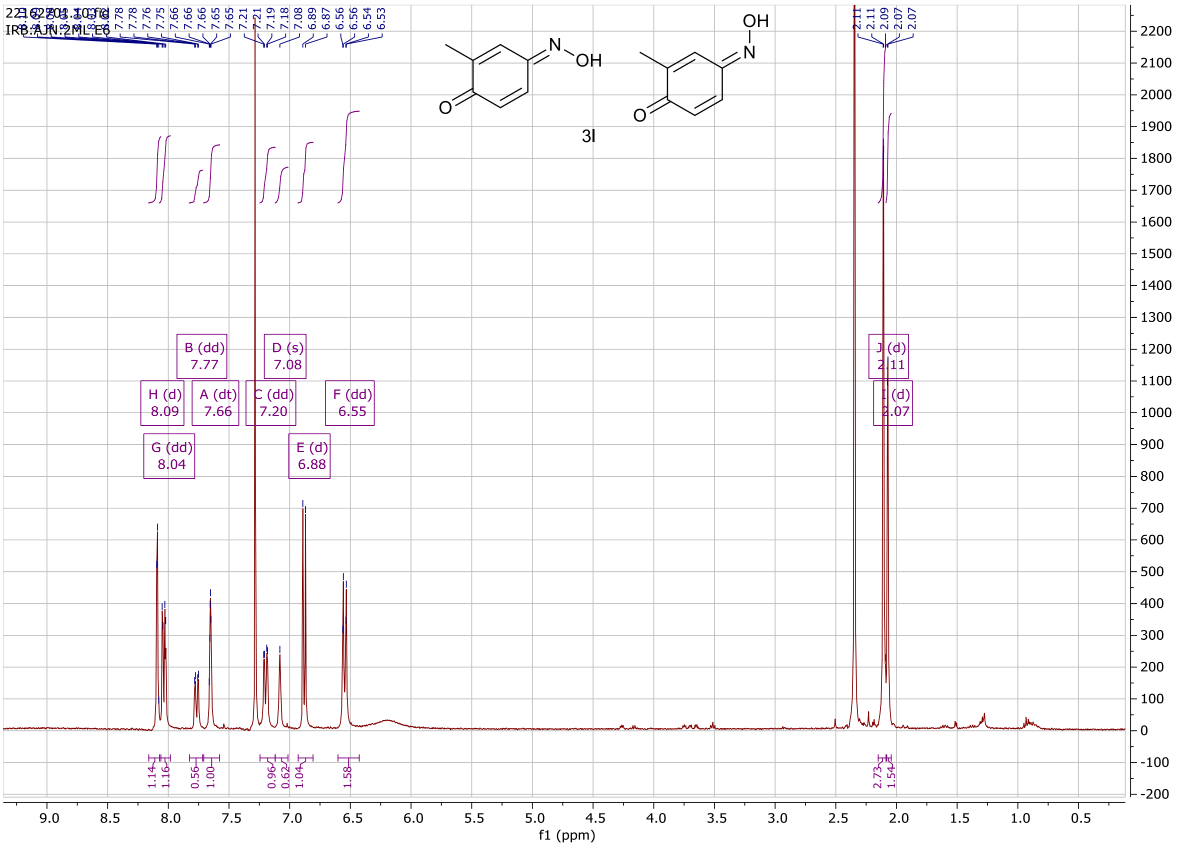

3l

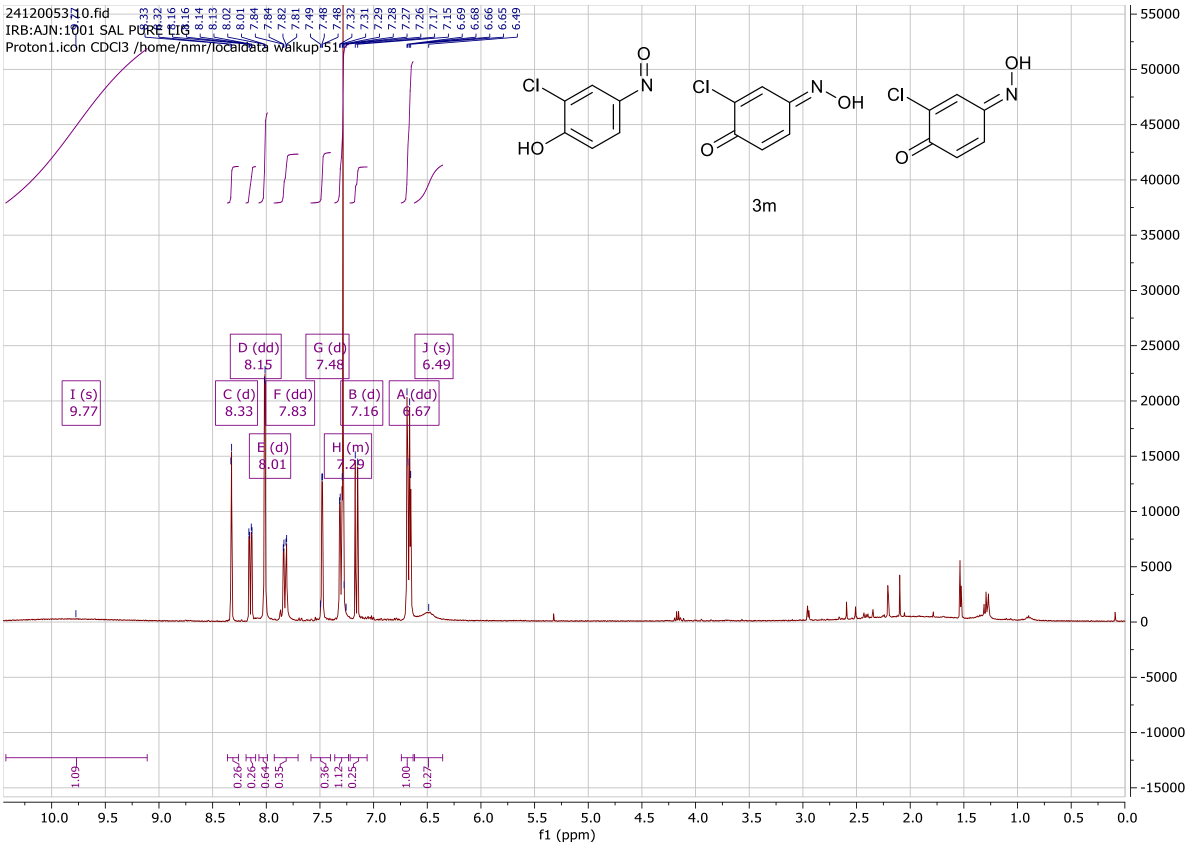

3m

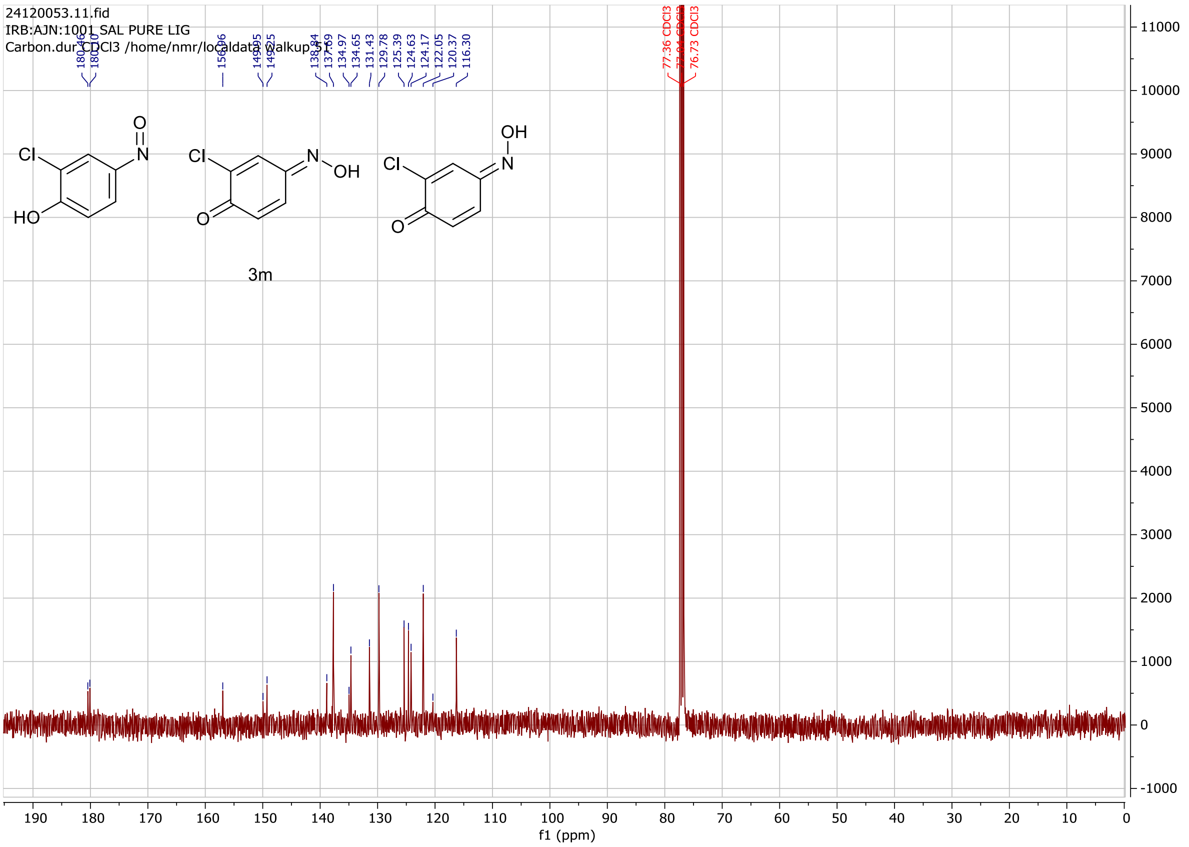

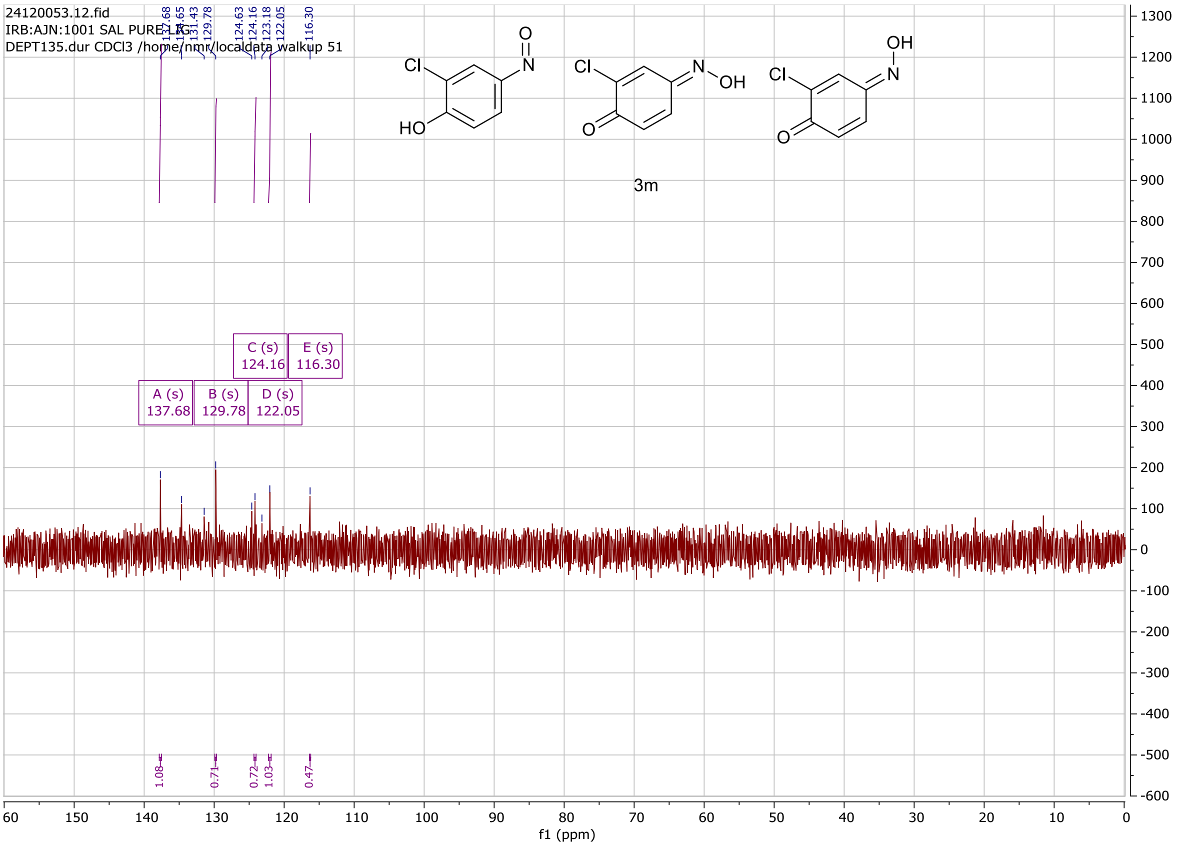

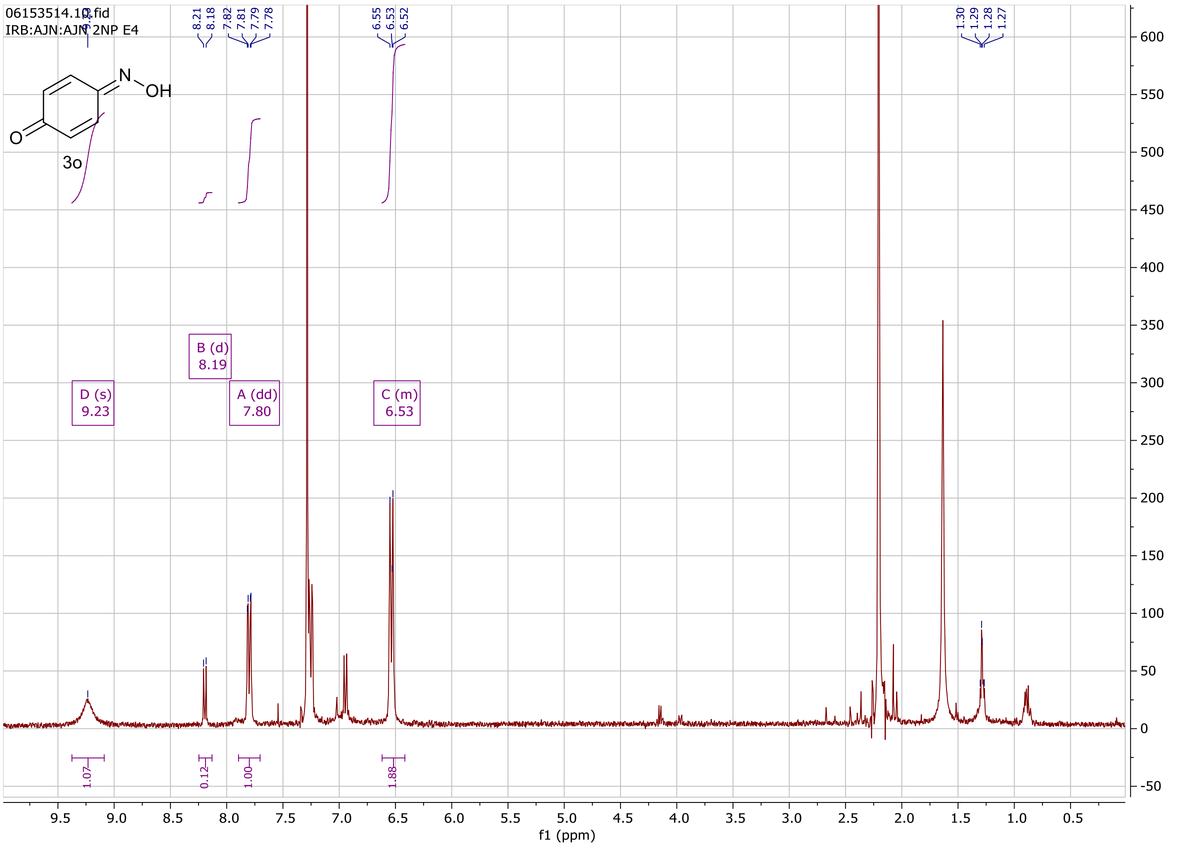
06153514.10.fid
IRB:AJN:AJN 2NP E4
N
OH
O
3o
B (d)
8.19
D (s)
9.23
A (dd)
7.80
C (m)
6.53
f1 (ppm)

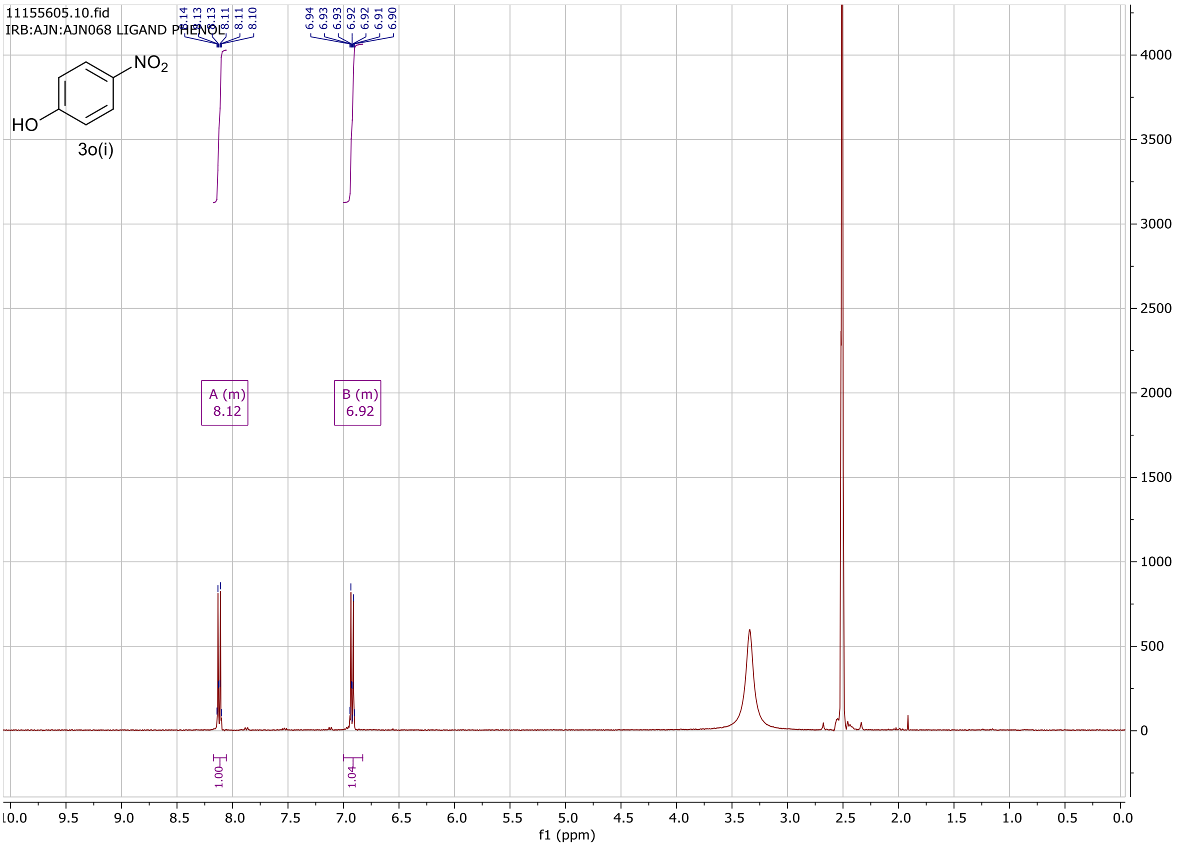
11155605.10.fid
IRB:AJN:AJN068 LIGAND PHENOL
NO2
HO
3o(i)
A (m)
8.12
B (m)
6.92
f1 (ppm)

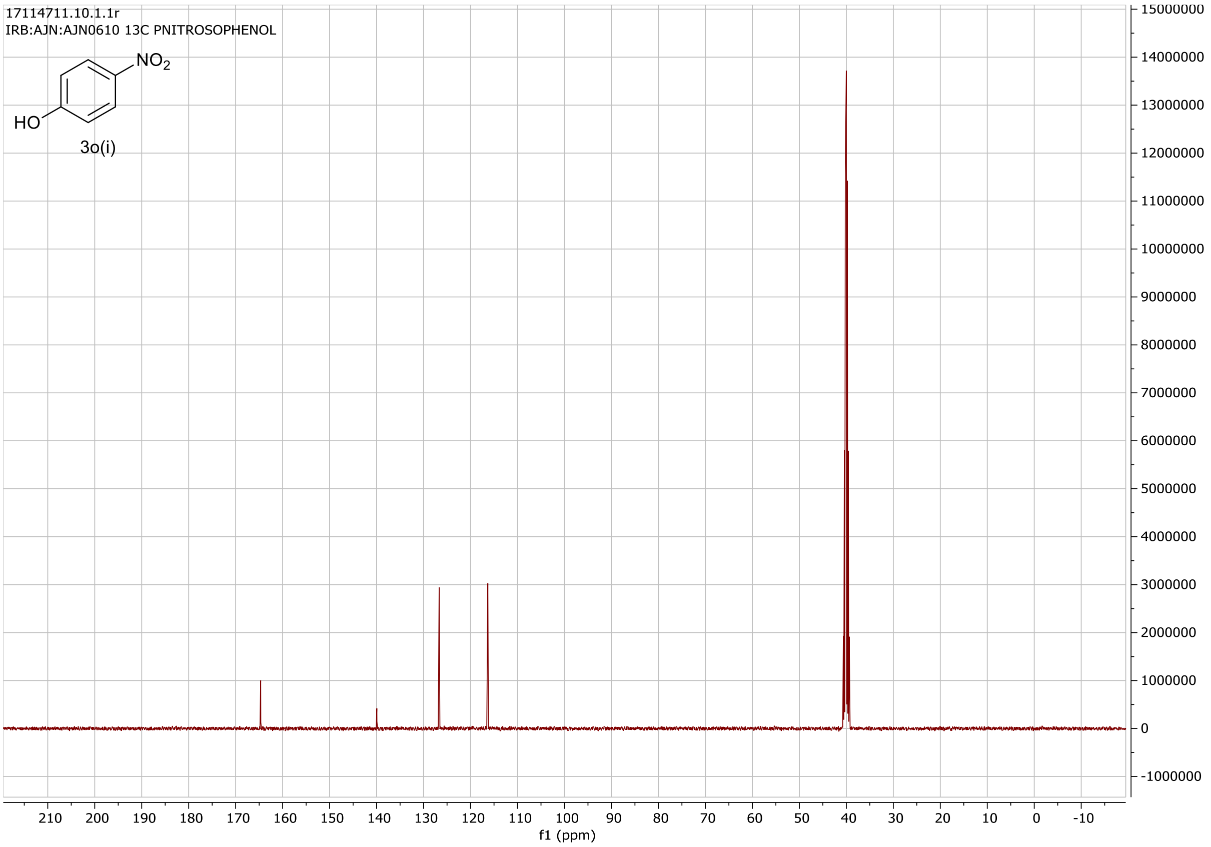
17114711.10.1.1r
IRB:AJN:AJN0610 13C PNITROSOPHENOL
NO2
HO
3o(i)
f1 (ppm)
210 200 190 180 170 160 150 140 130 120 110 100 90 80 70 60 50 40 30 20 10 0 -10
15000000 14000000 13000000 12000000 11000000 10000000 9000000 8000000 7000000 6000000 5000000 4000000 3000000 2000000 1000000 0 -1000000

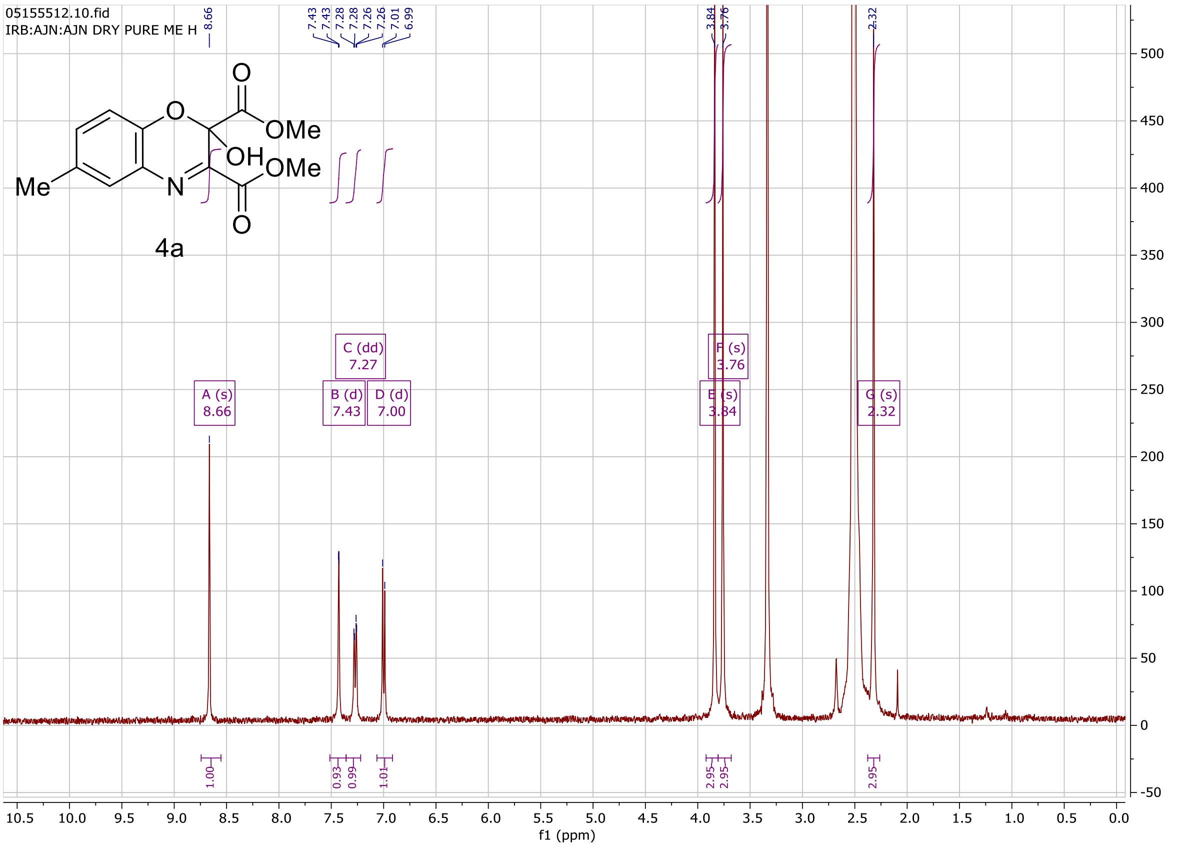
05155512.10.fid
IRB:AJN:AJN DRY PURE ME H
8.66
7.43
7.43
7.28
7.28
7.26
7.26
7.01
6.99
3.84
3.76
2.32
O
OMe
OH
OMe
Me
N
O
4a
C (dd)
7.27
F (s)
3.76
A (s)
8.66
B (d)
7.43
D (d)
7.00
E (s)
3.84
G (s)
2.32
1.00
0.93
0.99
1.01
2.95
2.95
2.95
f1 (ppm)

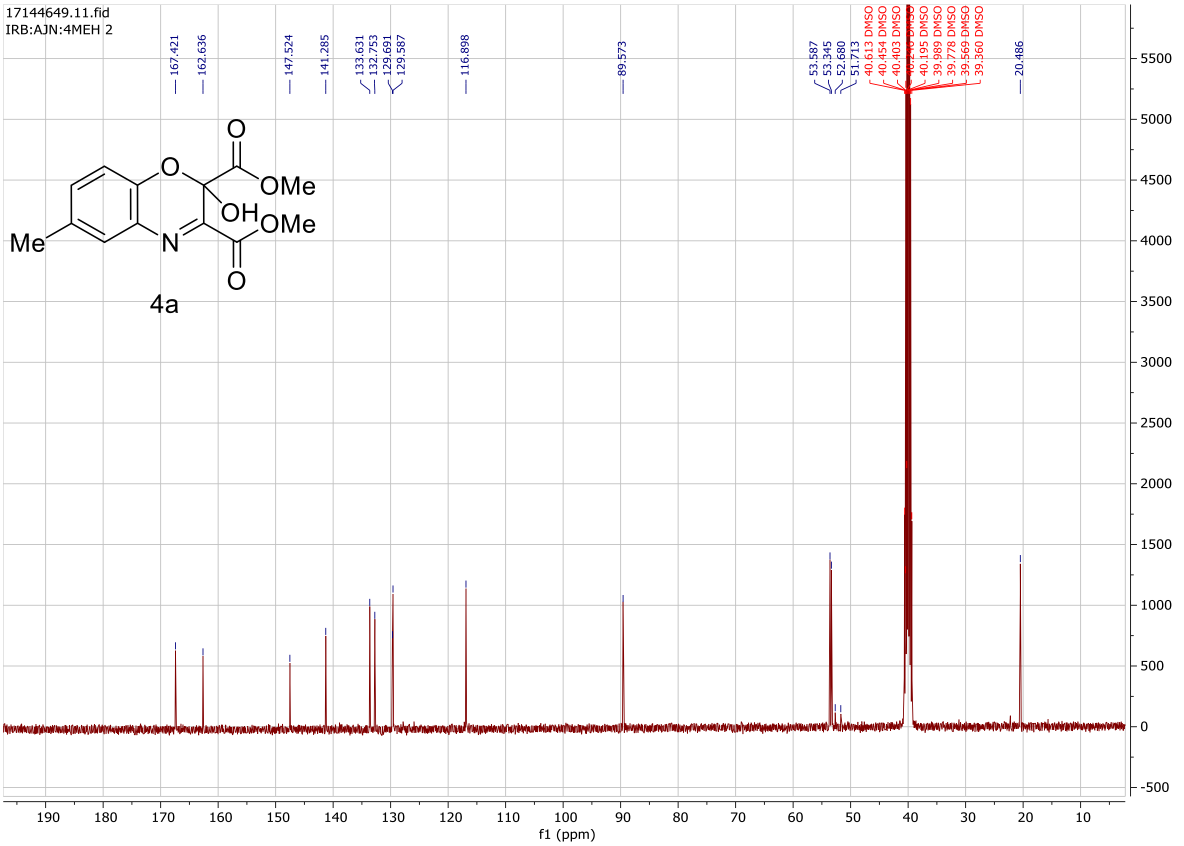
17144649.11.fid
IRB:AJN:4MEH 2
167.421
162.636
147.524
141.285
133.631
132.753
129.691
129.587
116.898
89.573
53.587
53.345
52.680
51.713
40.613 DMSO
40.454 DMSO
40.403 DMSO
40.195 DMSO
39.989 DMSO
39.778 DMSO
39.569 DMSO
39.360 DMSO
20.486
O
OMe
OH
OMe
Me
N
O
4a
f1 (ppm)

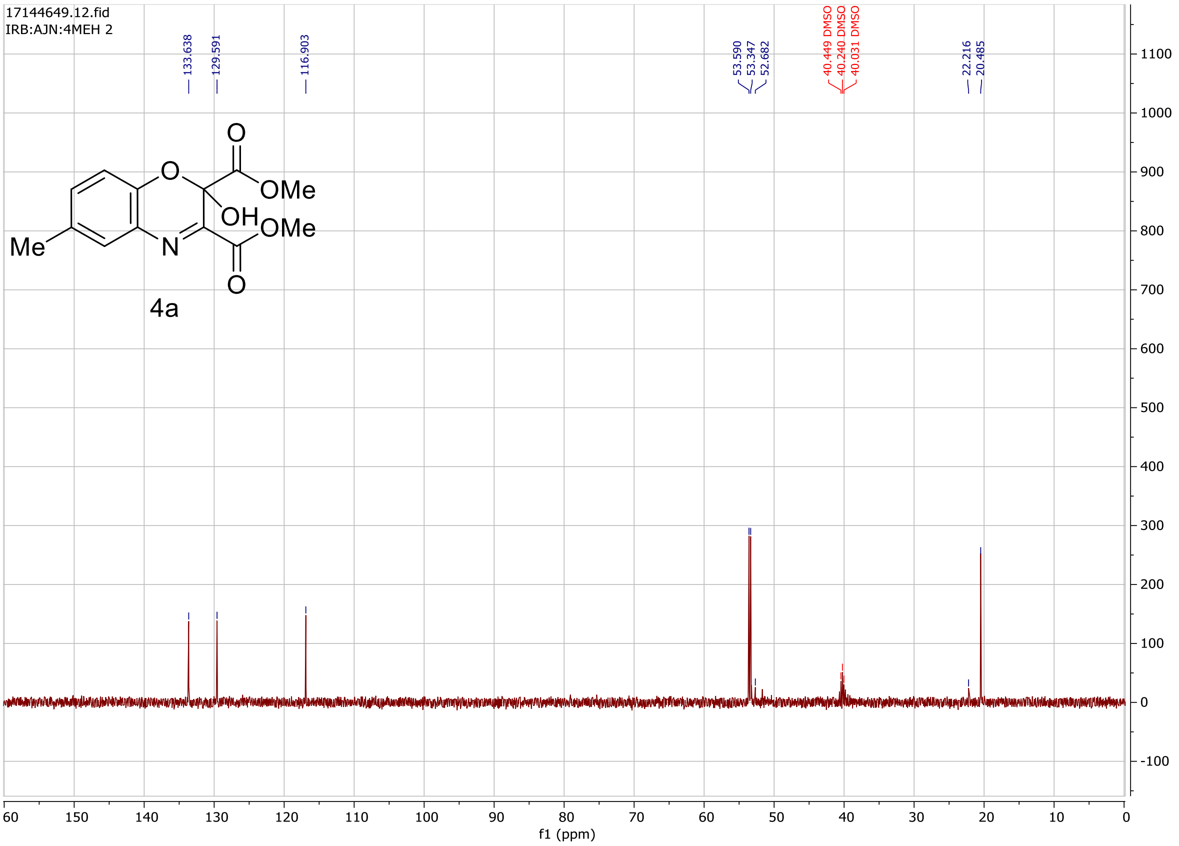

17144649.12.fid
IRB:AJN:4MEH 2
133.638
129.591
116.903
53.590
53.347
52.682
40.449 DMSO
40.240 DMSO
40.031 DMSO
22.216
20.485
4a
f1 (ppm)

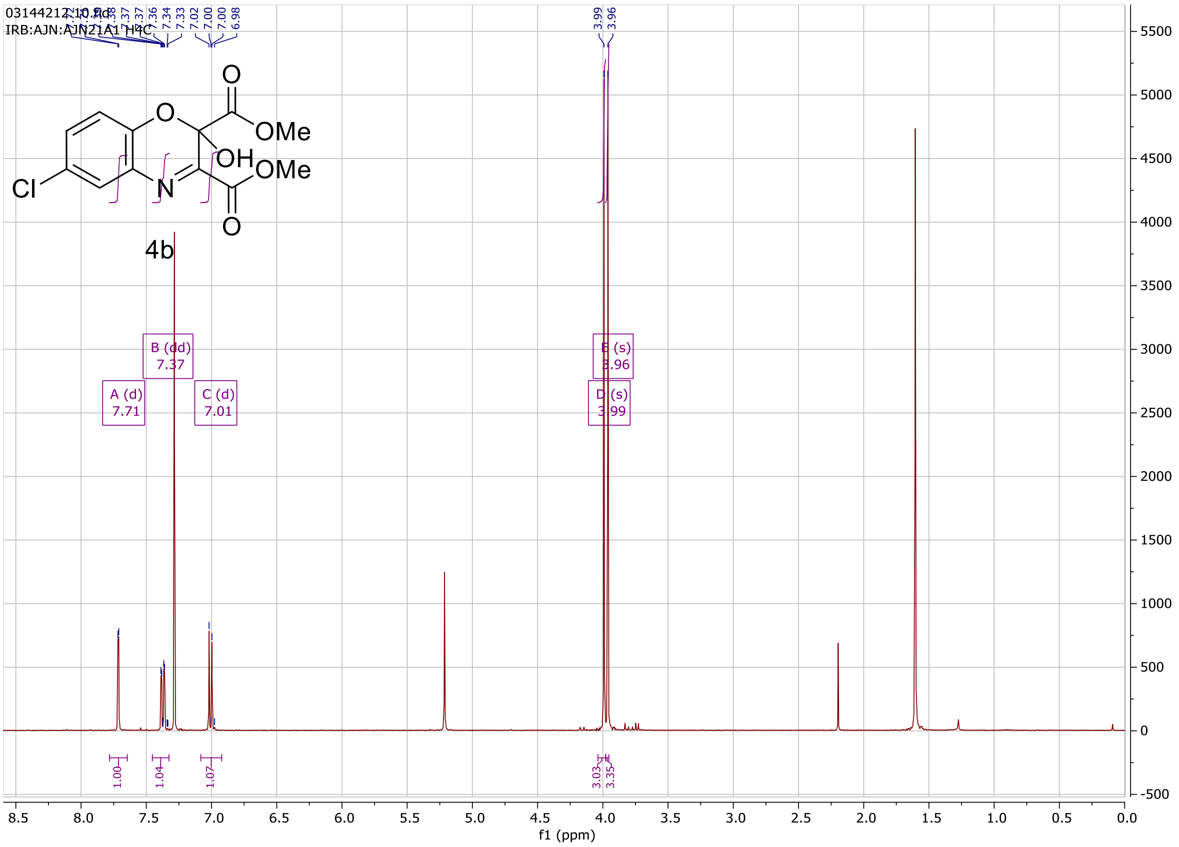

IRB:AJN:AJN21A1 H4C
3.99
3.96
4b
B (dd)
7.37
A (d)
7.71
C (d)
7.01
E (s)
3.96
D (s)
3.99
f1 (ppm)

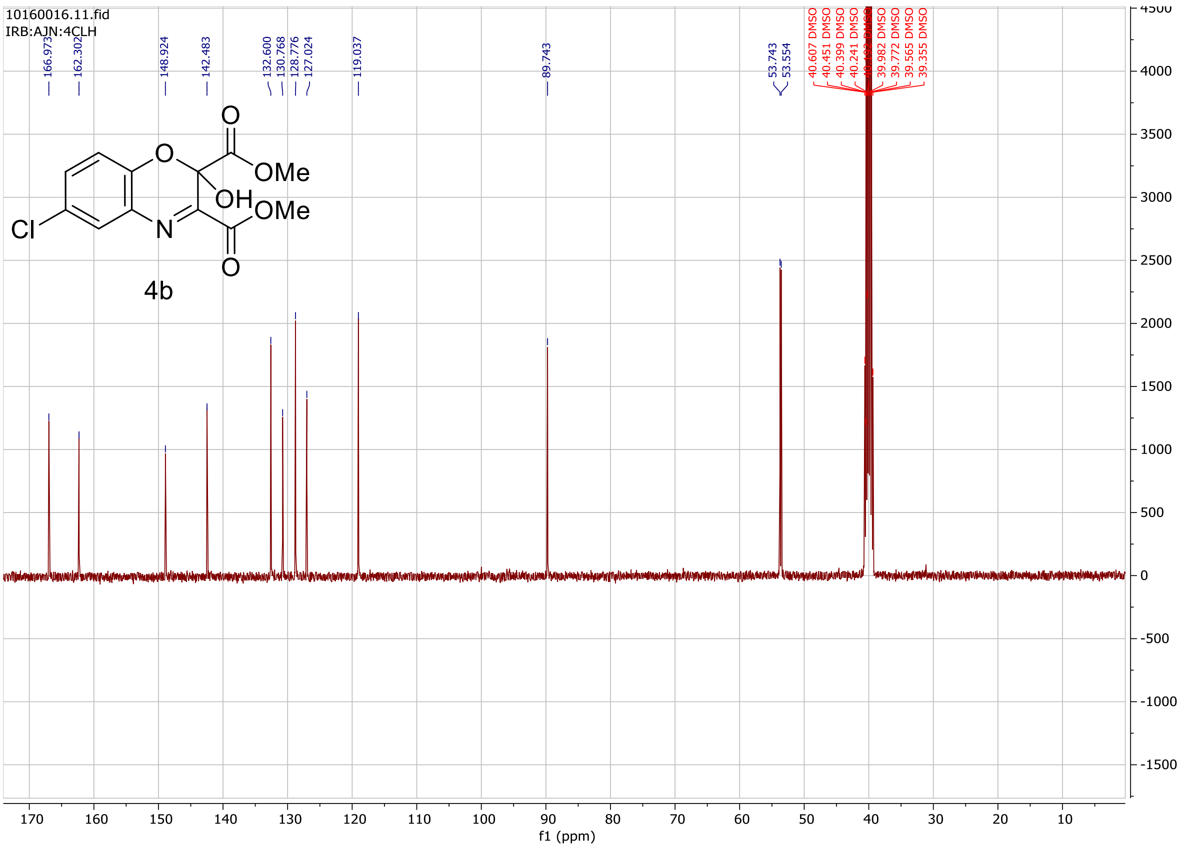
10160016.11.fid
IRB:AJN:4CLH
166.973
162.302
148.924
142.483
132.600
130.768
128.776
127.024
119.037
89.743
53.743
53.554
40.607 DMSO
40.451 DMSO
40.399 DMSO
40.241 DMSO
39.982 DMSO
39.772 DMSO
39.565 DMSO
39.355 DMSO
Cl
O
N
OH
OMe
OMe
4b
f1 (ppm)

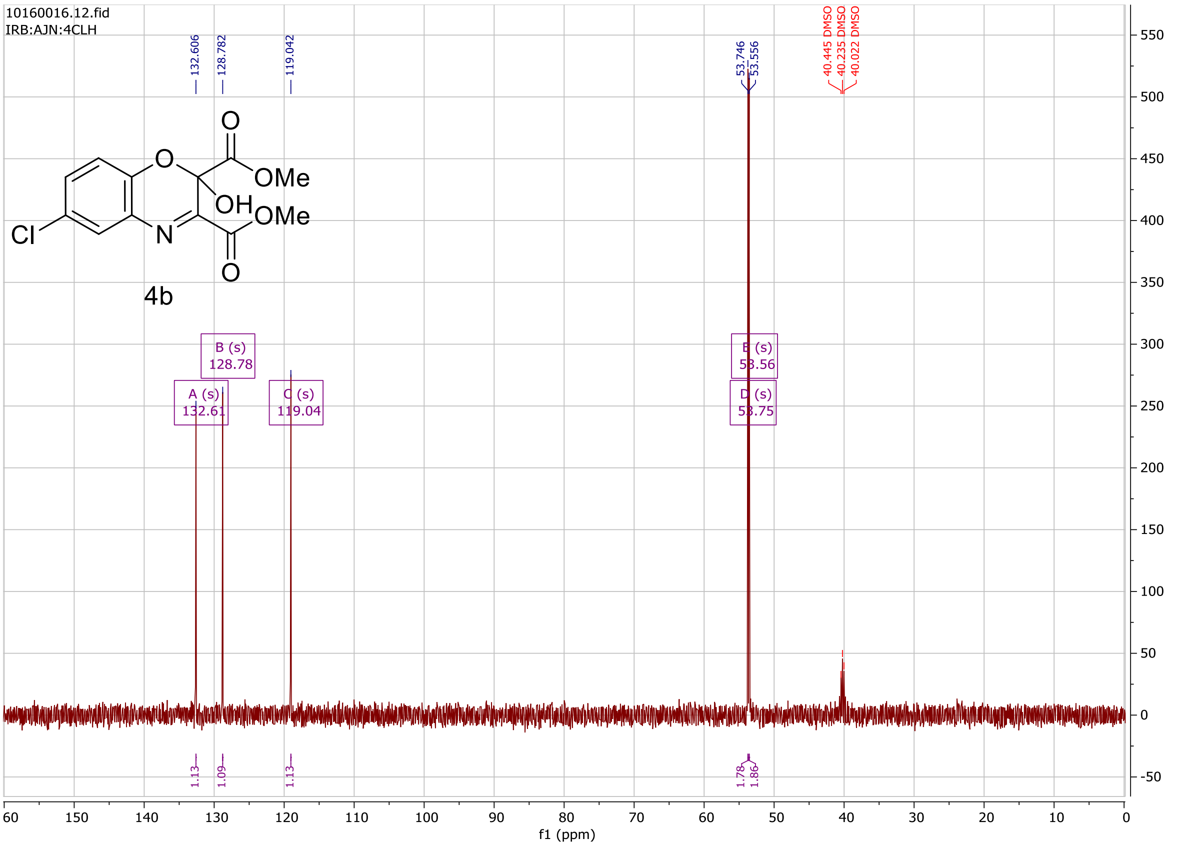
10160016.12.fid
IRB:AJN:4CLH
132.606
128.782
119.042
53.746
53.556
40.445 DMSO
40.235 DMSO
40.022 DMSO
Cl
O
N
OH
OMe
OMe
4b
B (s)
128.78
A (s)
132.61
C (s)
119.04
E (s)
53.56
D (s)
53.75
1.13
1.09
1.13
1.78
1.86
f1 (ppm)

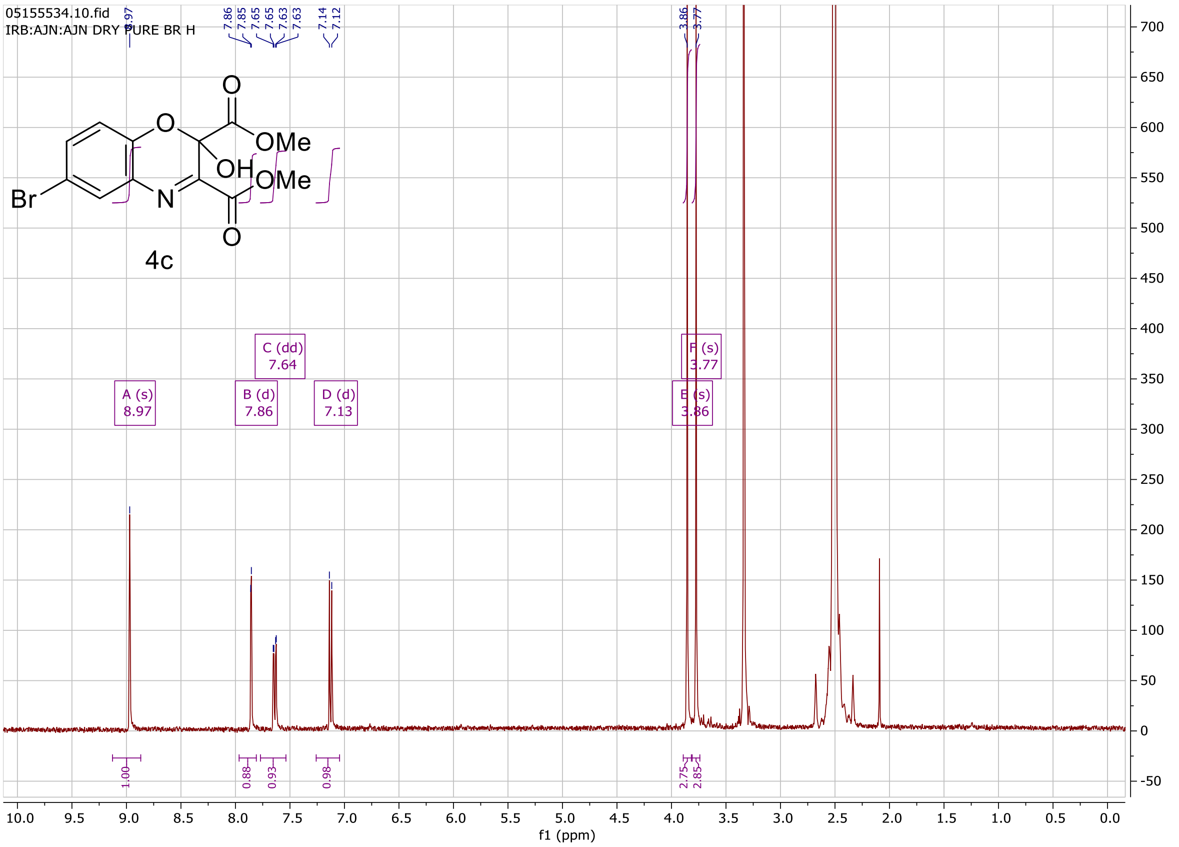
05155534.10.fid
IRB:AJN:AJN DRY PURE BR H
8.97
7.86
7.85
7.65
7.65
7.63
7.63
7.14
7.12
3.86
3.77
Br
O
N
O
OMe
OH
OMe
O
4c
A (s)
8.97
B (d)
7.86
C (dd)
7.64
D (d)
7.13
E (s)
3.86
F (s)
3.77
1.00
0.88
0.93
0.98
2.75
2.85
f1 (ppm)

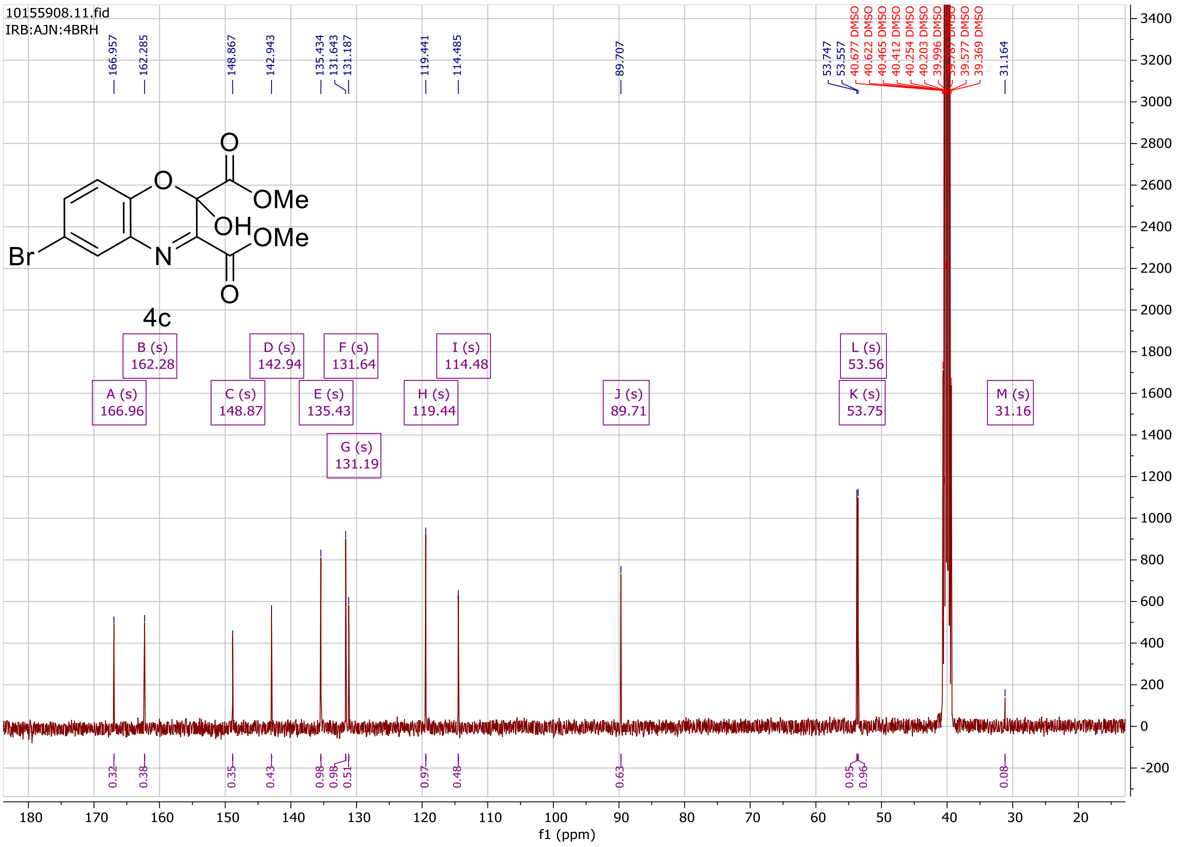
10155908.11.fid
IRB:AJN:4BRH
166.957
162.285
148.867
142.943
135.434
131.643
131.187
119.441
114.485
89.707
53.747
53.557
40.677 DMSO
40.622 DMSO
40.465 DMSO
40.412 DMSO
40.254 DMSO
40.203 DMSO
39.996 DMSO
39.787 DMSO
39.577 DMSO
39.369 DMSO
31.164
Br
O
N
O
OMe
OH
OMe
O
4c
A (s)
166.96
B (s)
162.28
C (s)
148.87
D (s)
142.94
E (s)
135.43
F (s)
131.64
G (s)
131.19
H (s)
119.44
I (s)
114.48
J (s)
89.71
K (s)
53.75
L (s)
53.56
M (s)
31.16
0.32
0.38
0.35
0.43
0.98
0.98
0.51
0.97
0.48
0.63
0.95
0.96
0.08
f1 (ppm)

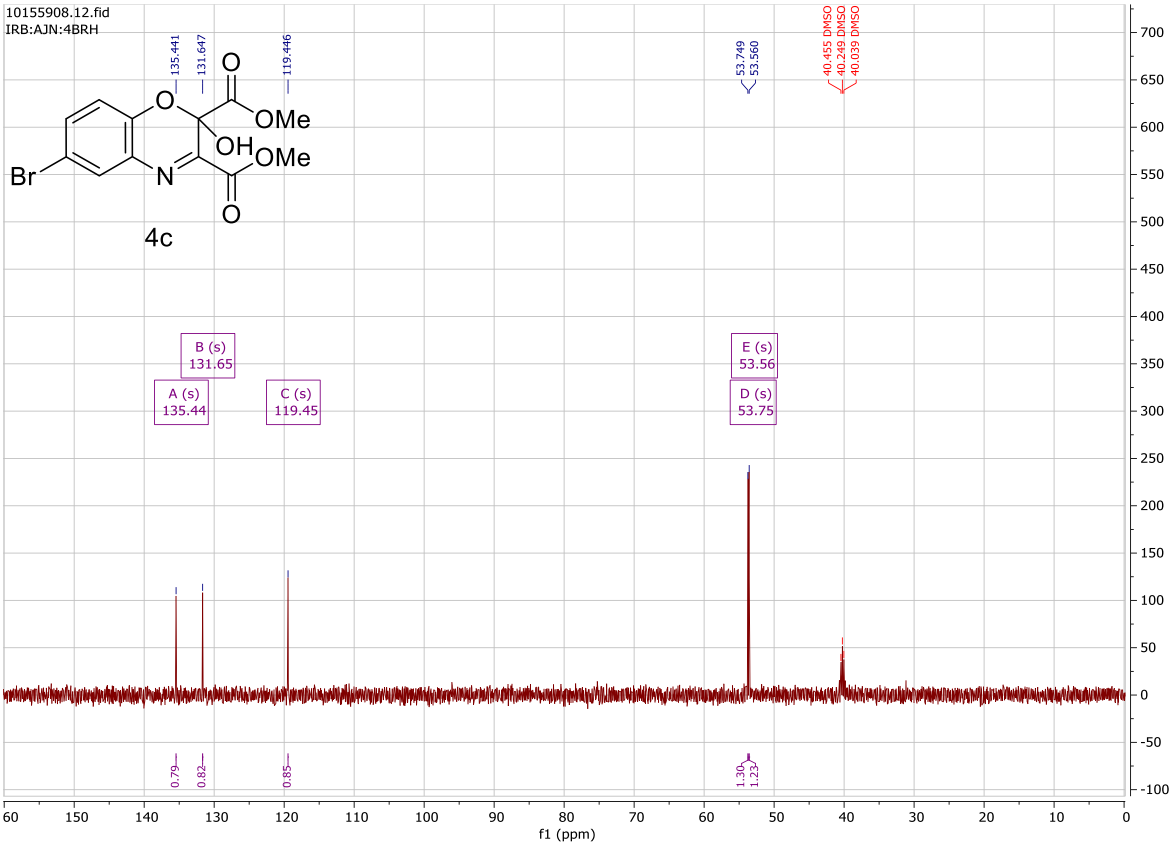
10155908.12.fid
IRB:AJN:4BRH
135.441
131.647
119.446
53.749
53.560
40.455 DMSO
40.249 DMSO
40.039 DMSO
O
OMe
OH
OMe
Br
N
O
4c
B (s)
131.65
A (s)
135.44
C (s)
119.45
E (s)
53.56
D (s)
53.75
0.79
0.82
0.85
1.30
1.23
f1 (ppm)

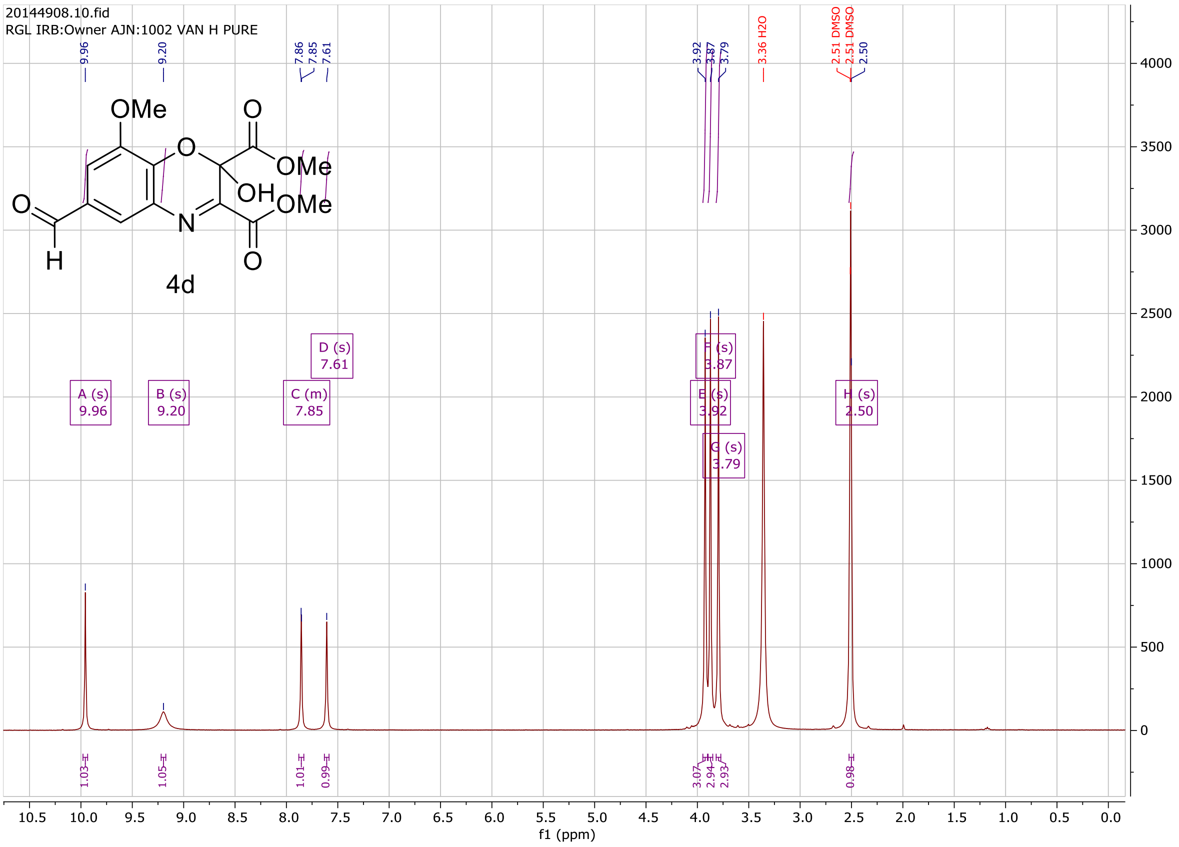
20144908.10.fid
RGL IRB:Owner AJN:1002 VAN H PURE
9.96
9.20
7.86
7.85
7.61
3.92
3.87
3.79
3.36 H2O
2.51 DMSO
2.51 DMSO
2.50
OMe
O
O
OMe
OH
OMe
O
N
H
O
4d
D (s)
7.61
A (s)
9.96
B (s)
9.20
C (m)
7.85
H (s)
2.50
G (s)
3.79
1.03
1.05
1.01
0.99
3.07
2.94
2.93
0.98
f1 (ppm)

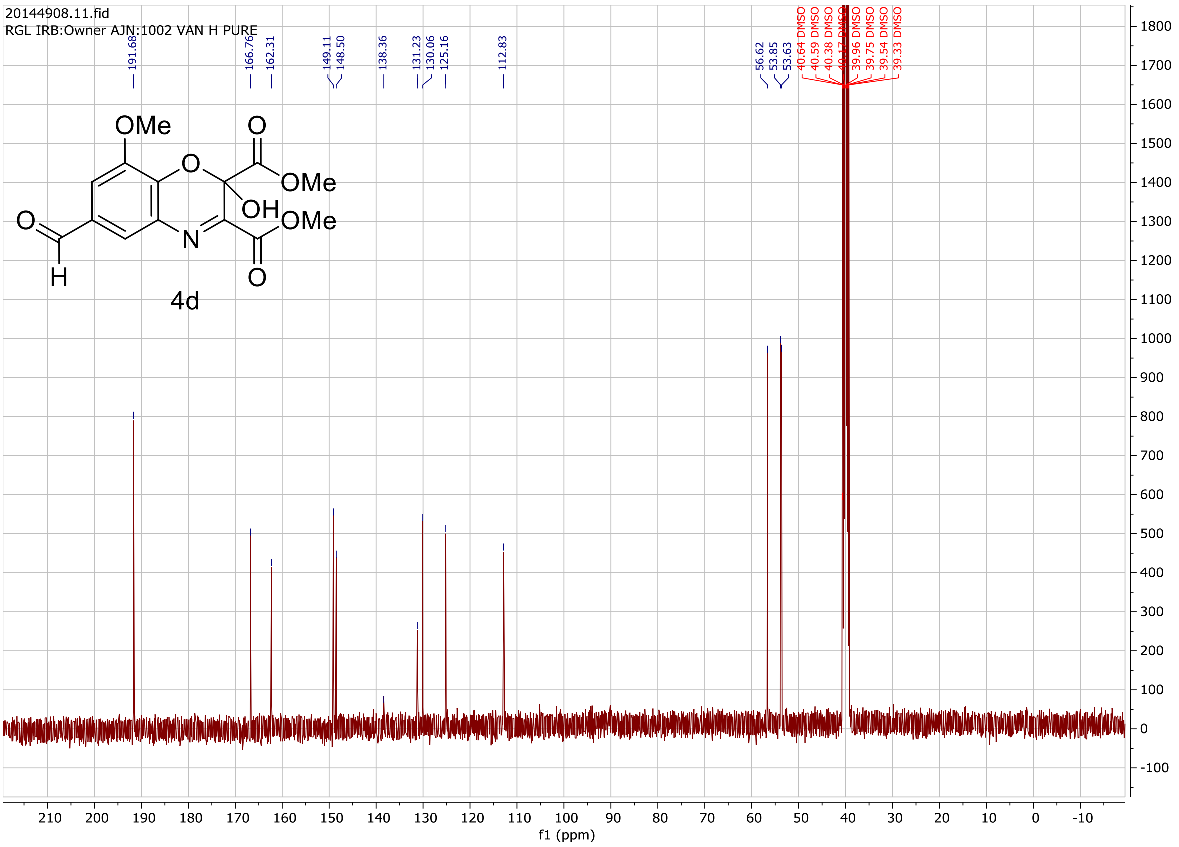

20144908.11.fid
RGL IRB:Owner AJN:1002 VAN H PURE
191.68
166.76
162.31
149.11
148.50
138.36
131.23
130.06
125.16
112.83
56.62
53.85
53.63
40.64 DMSO
40.59 DMSO
40.38 DMSO
39.96 DMSO
39.75 DMSO
39.54 DMSO
39.33 DMSO
OMe
O
OMe
OH
OMe
N
H
4d
f1 (ppm)

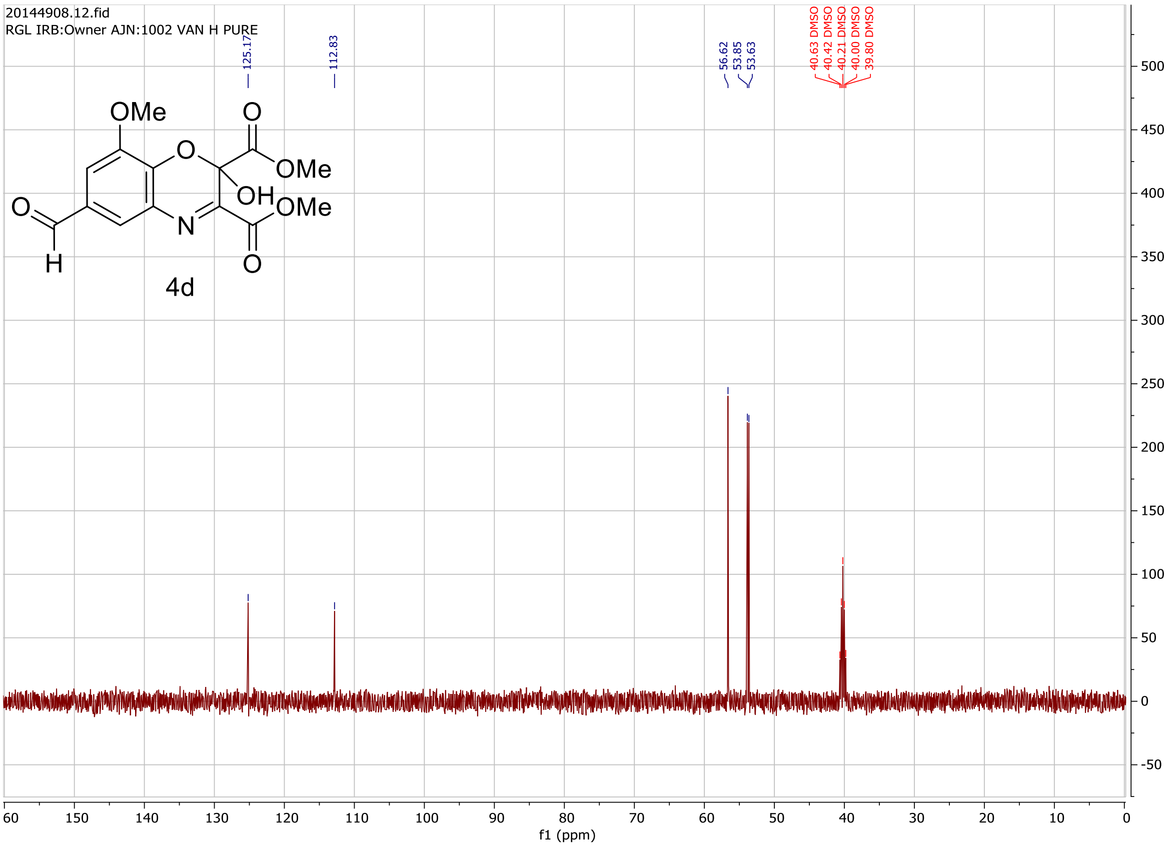

20144908.12.fid
RGL IRB:Owner AJN:1002 VAN H PURE
125.17
112.83
56.62
53.85
53.63
40.63 DMSO
40.42 DMSO
40.21 DMSO
40.00 DMSO
39.80 DMSO
OMe
O
OMe
OH
OMe
N
H
4d
f1 (ppm)

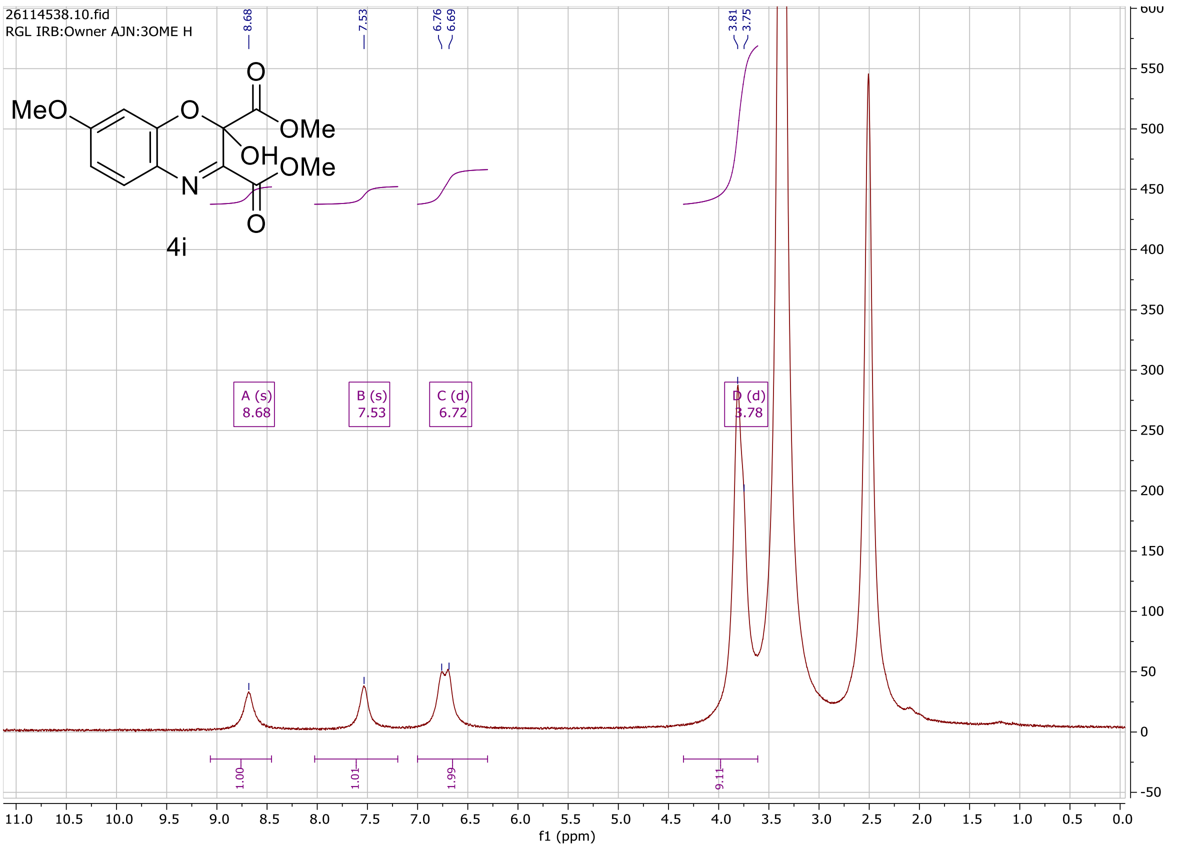
26114538.10.fid
RGL IRB:Owner AJN:3OME H
8.68
7.53
6.76
6.69
3.81
3.75
MeO
O
OMe
OH
OMe
N
O
O
4i
A (s)
8.68
B (s)
7.53
C (d)
6.72
D (d)
3.78
1.00
1.01
1.99
9.11
f1 (ppm)

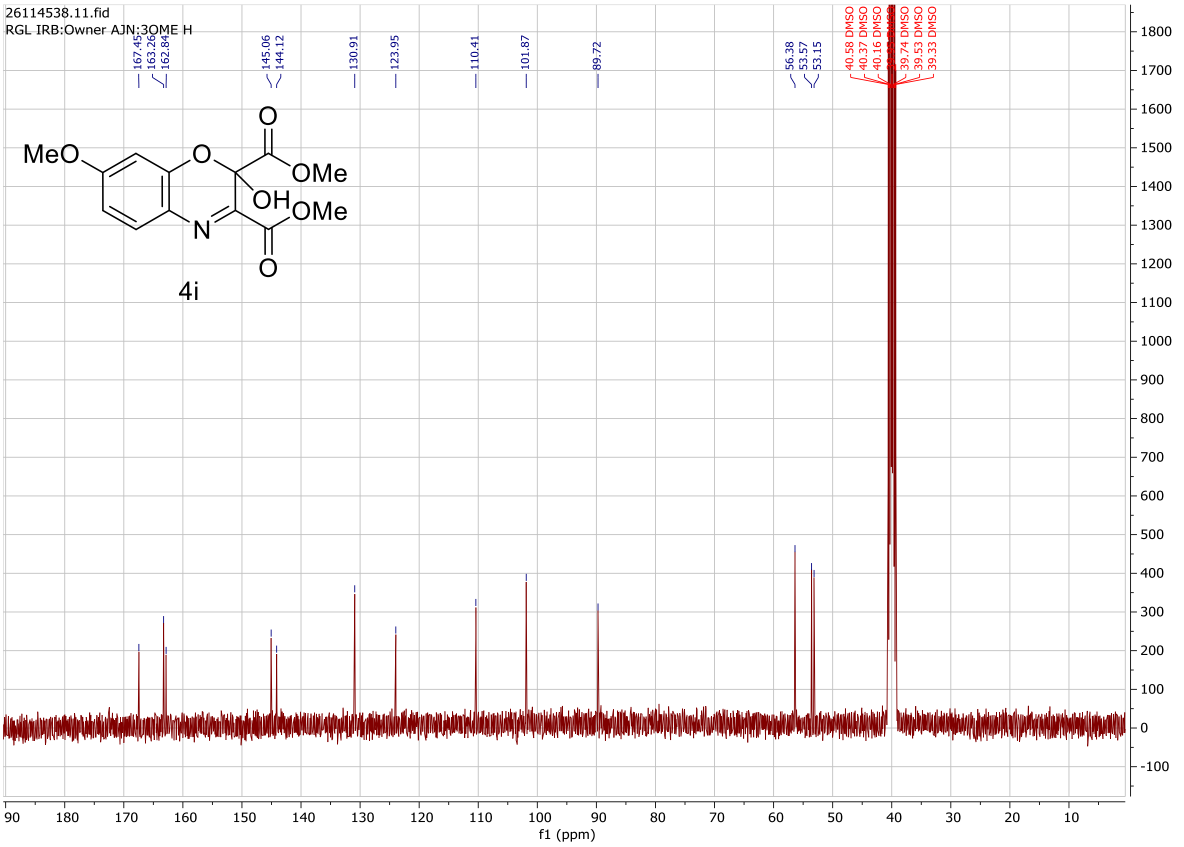
26114538.11.fid
RGL IRB:Owner AJN:3OME H
167.45
163.26
162.84
145.06
144.12
130.91
123.95
110.41
101.87
89.72
56.38
53.57
53.15
40.58 DMSO
40.37 DMSO
40.16 DMSO
39.74 DMSO
39.53 DMSO
39.33 DMSO
MeO
O
OMe
OH
OMe
N
O
O
4i
f1 (ppm)

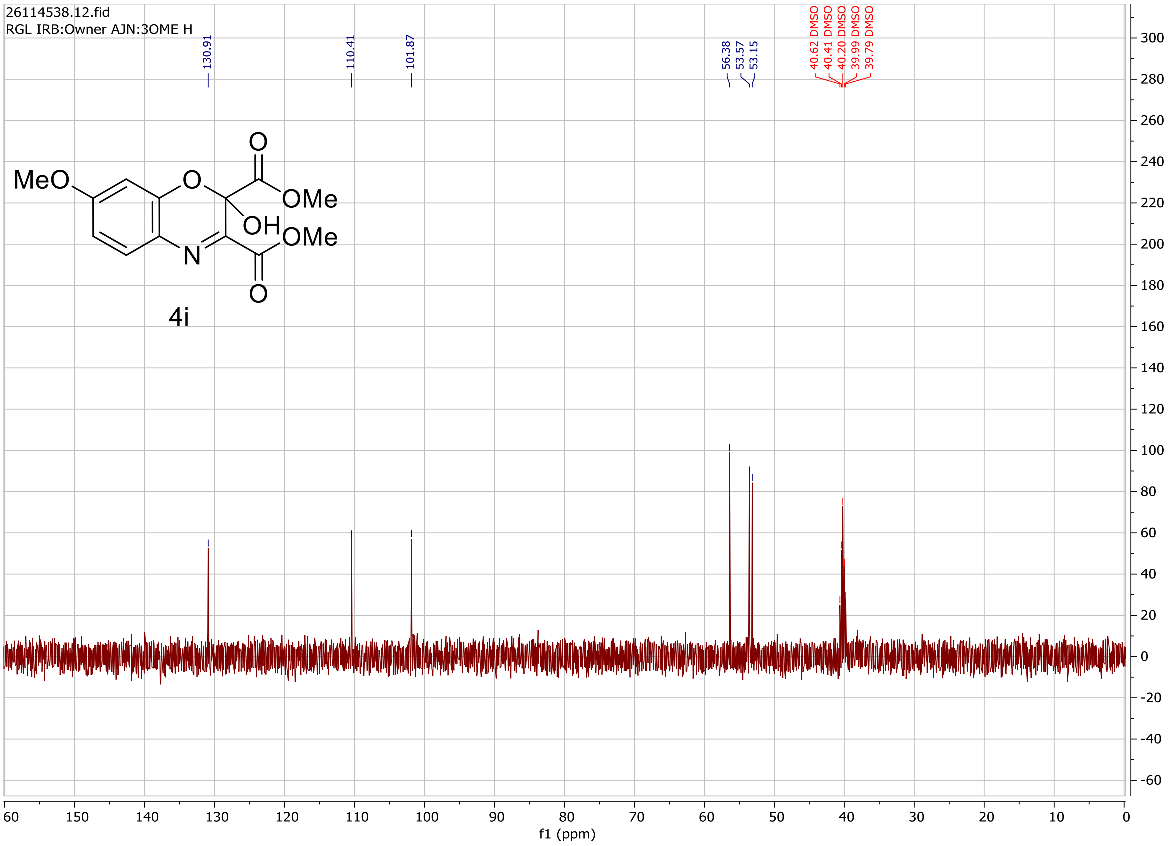
26114538.12.fid
RGL IRB:Owner AJN:3OME H
130.91
110.41
101.87
56.38
53.57
53.15
40.62 DMSO
40.41 DMSO
40.20 DMSO
39.99 DMSO
39.79 DMSO
MeO
O
OMe
OH
OMe
N
4i
f1 (ppm)

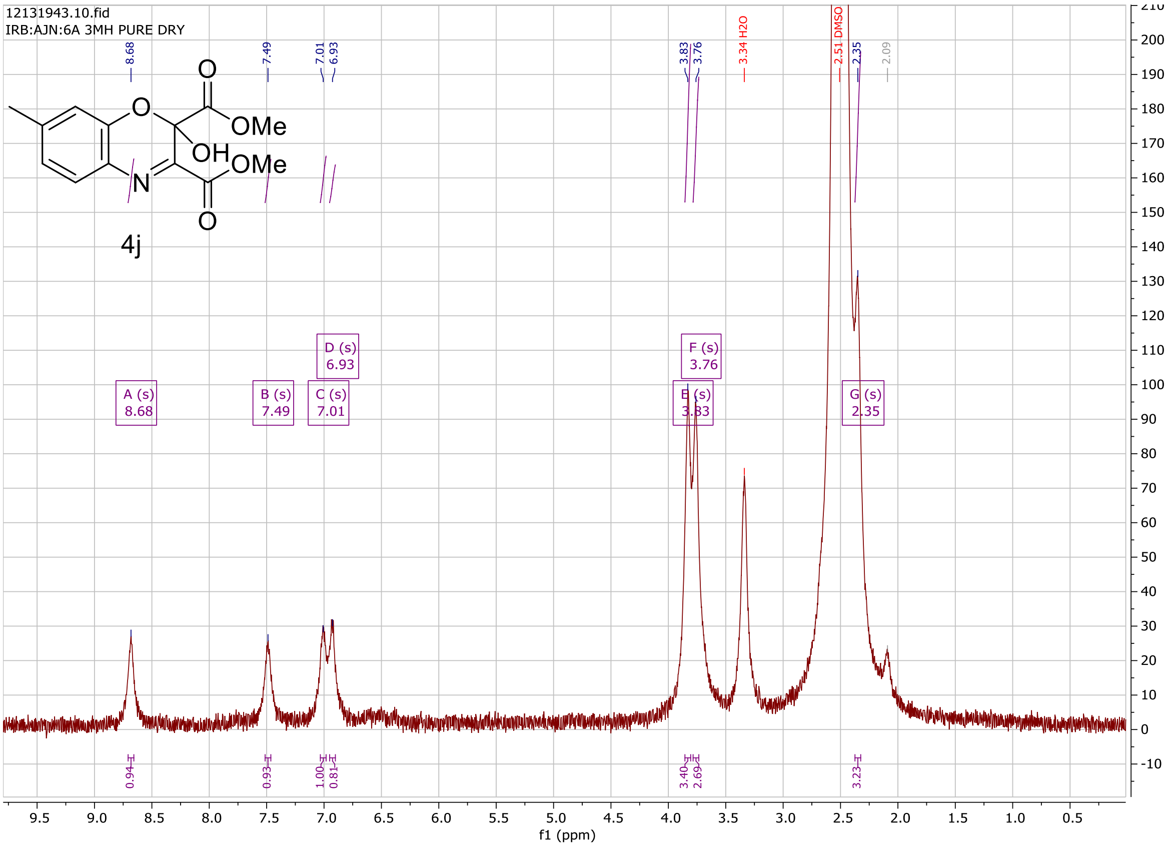
12131943.10.fid
IRB:AJN:6A 3MH PURE DRY
8.68
7.49
7.01
6.93
3.83
3.76
3.34 H2O
2.51 DMSO
2.35
2.09
O
OMe
OH
OMe
N
4j
A (s) 8.68
B (s) 7.49
C (s) 7.01
D (s) 6.93
E (s) 3.83
F (s) 3.76
G (s) 2.35
0.94
0.93
1.00
0.81
3.40
2.69
3.23
f1 (ppm)

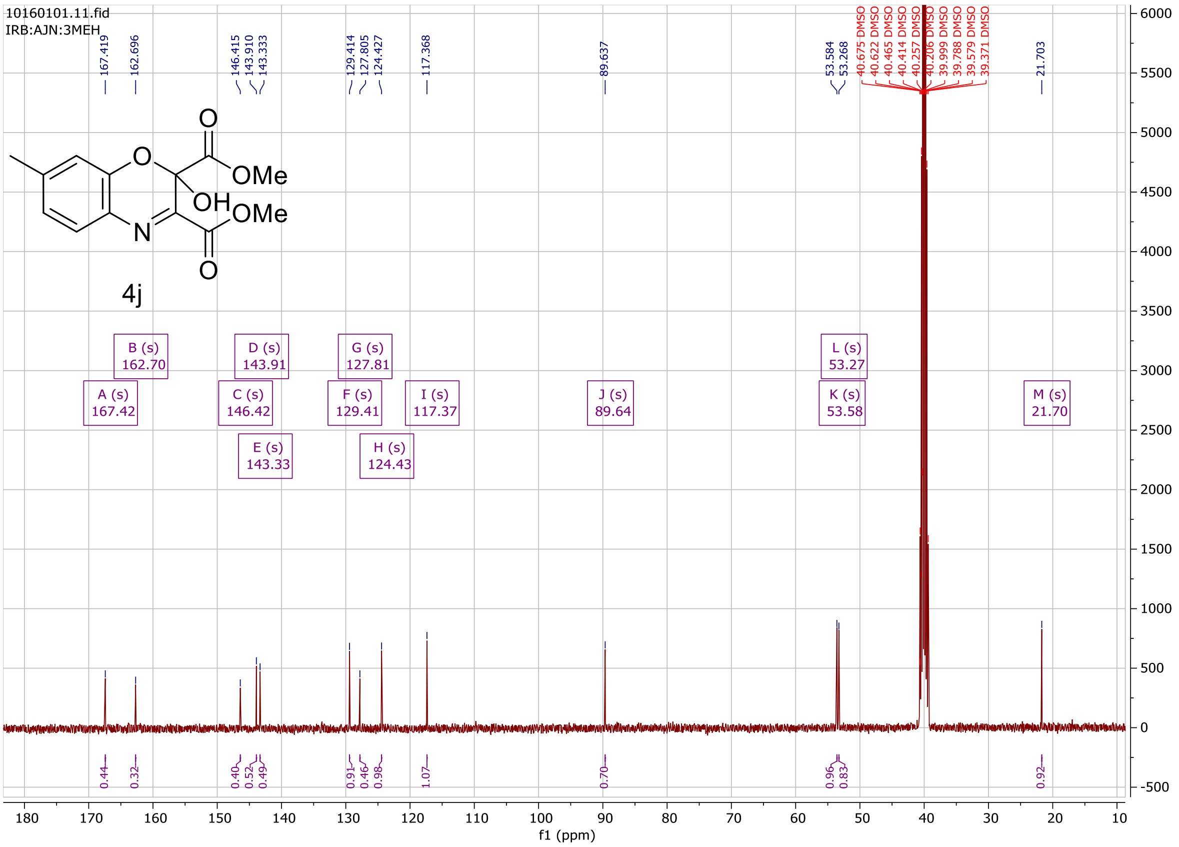

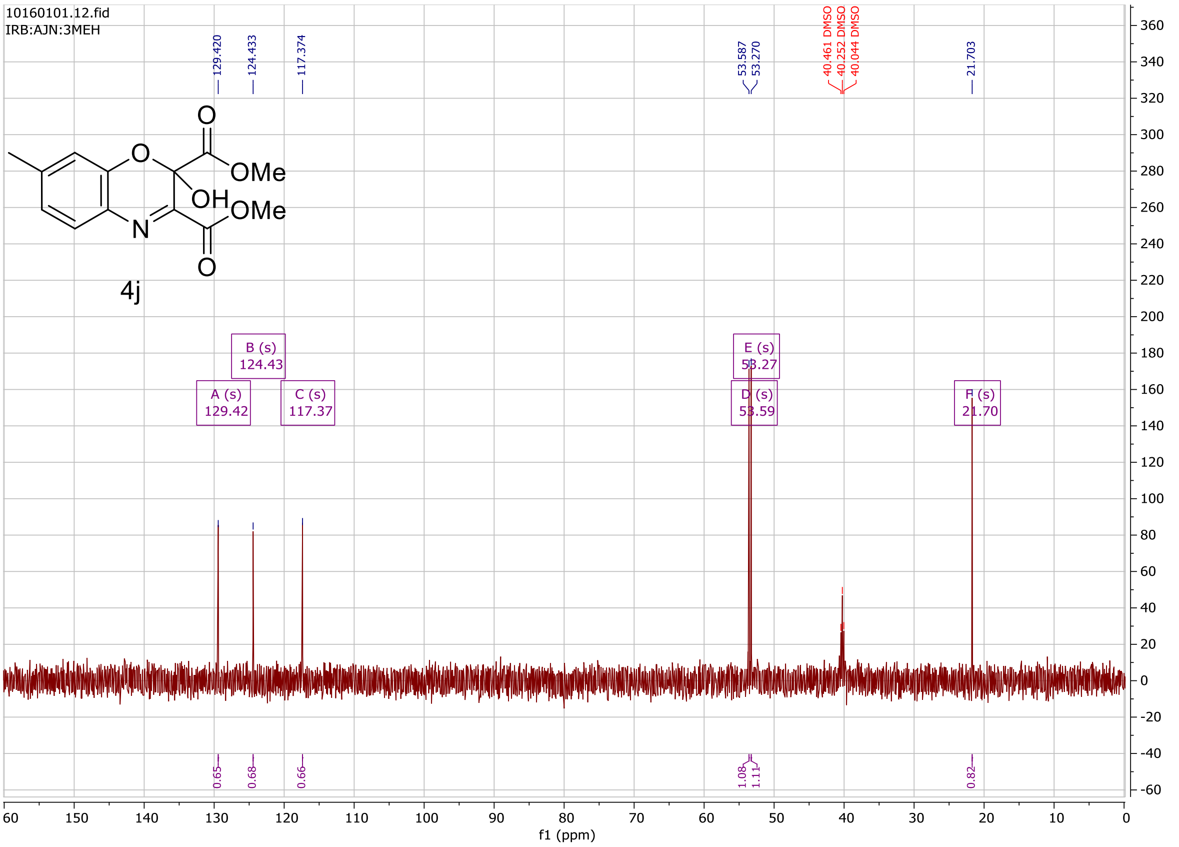

Accurate Mass Spectra of Selected compounds:

Elemental Composition Report **Page 1**

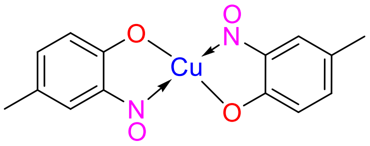

2a, 82% yield

Single Mass Analysis
Tolerance = 5.0 mDa / DBE: min = -1.5, max = 50.0
Element prediction: Off
Number of isotope peaks used for i-FIT = 3

Monoisotopic Mass, Even Electron Ions
815 formula(e) evaluated with 10 results within limits (up to 50 closest results for each mass)
Elements Used:
C: 0-35 H: 0-60 N: 0-3 O: 0-5 Cl: 0-2 I: 0-2 63Cu: 0-1

LCT Premier 350 °C AJN_4M_4148 79 (0.633) Cm (77:79)
1: TOF MS AP+ 4.76e+004

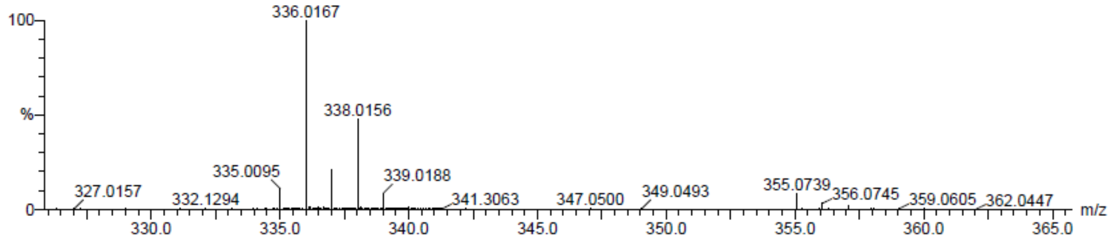

Mass	Calc. Mass	mDa	PPM	DBE	i-FIT	i-FIT (Norm)	Formula
Minimum:				-1.5			
Maximum:		5.0	5.0	50.0			
336.0167	336.0171	-0.4	-1.2	9.5	458.6	8.6	C14 H13 N2 O4 63Cu
	336.0176	-0.9	-2.7	15.5	450.7	0.7	C17 H7 N3 O3 Cl
	336.0154	1.3	3.9	6.5	452.7	2.8	C11 H12 N3 O5 Cl2
	336.0190	-2.3	-6.8	4.5	452.0	2.0	C13 H18 O4 Cl 63Cu
	336.0194	-2.7	-8.0	10.5	452.3	2.3	C16 H12 N O3 Cl2
	336.0198	-3.1	-9.2	24.5	458.9	8.9	C23 H2 N3 O
	336.0209	-4.2	-12.5	4.5	458.7	8.7	C10 H15 N3 O2 I
	336.0124	4.3	12.8	-1.5	459.2	9.2	C8 H22 N2 I 63Cu

Elemental Composition Report **Page 1**

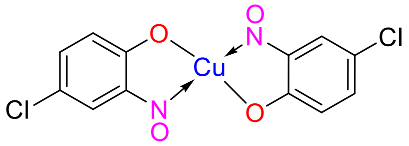

2b, 55 % yield

Single Mass Analysis
Tolerance = 5.0 mDa / DBE: min = -1.5, max = 50.0
Element prediction: Off
Number of isotope peaks used for i-FIT = 3

Monoisotopic Mass, Odd and Even Electron Ions
989 formula(e) evaluated with 11 results within limits (up to 50 closest results for each mass)
Elements Used:
C: 0-35 H: 0-60 N: 0-4 O: 0-8 Cl: 0-2 63Cu: 0-1
LCT Premier 350 °C AJN_4CL_4168 102 (0.825) Cm (102:116-13:88)
1: TOF MS AP+ 1.73e+005

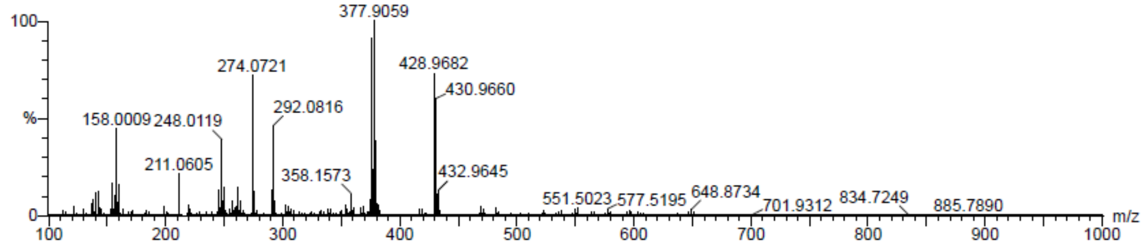

Mass	Calc. Mass	mDa	PPM	DBE	i-FIT	i-FIT (Norm)	Formula
Minimum:				-1.5			
Maximum:		5.0	5.0	50.0			
374.8990	374.8991	-0.1	-0.3	19.0	634.2	1.2	C18 O6 63Cu
	374.8996	-0.6	-1.6	14.5	635.9	2.9	C15 H3 N O5 Cl 63Cu
	374.8982	0.8	2.1	15.0	636.0	3.0	C13 H N4 O4 Cl 63Cu
	374.9001	-1.1	-2.9	10.0	635.8	2.8	C12 H6 N2 O4 Cl2 63Cu
	374.8974	1.6	4.3	5.5	635.9	2.9	C9 H8 N O7 Cl2 63Cu
	374.8969	2.1	5.6	10.0	636.0	3.0	C12 H5 O8 Cl 63Cu
	374.8960	3.0	8.0	6.0	636.0	3.0	C7 H6 N4 O6 Cl2 63Cu
	374.9023	-3.3	-8.8	19.0	635.8	2.8	C18 H N2 O2 Cl 63Cu

Elemental Composition Report

Page 1

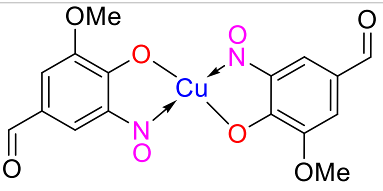

2d, 63% yield

Single Mass Analysis

Tolerance = 5.0 PPM / DBE: min = -1.5, max = 50.0
Element prediction: Off
Number of isotope peaks used for i-FIT = 3

Monoisotopic Mass, Even Electron Ions
506 formula(e) evaluated with 3 results within limits (up to 50 best isotopic matches for each mass)
Elements Used:
C: 0-60 H: 0-120 N: 0-5 O: 0-8 63Cu: 0-1

AJN_VAN_128456 97 (0.776) Cm (85:104)

1: TOF MS AP+
1.67e+005

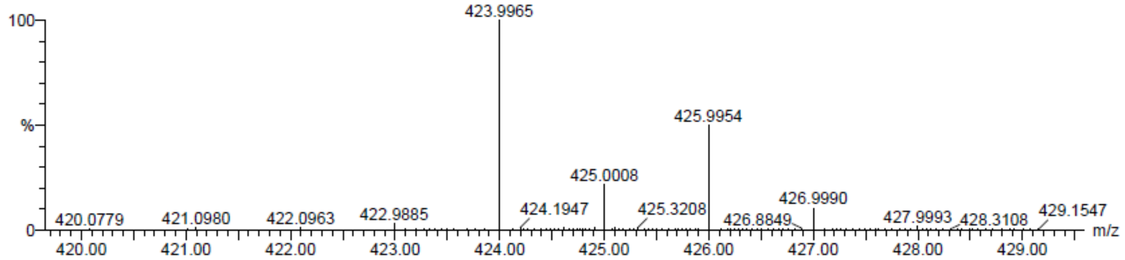

Minimum:				-1.5			
Maximum:		5.0	5.0	50.0			
Mass	Calc. Mass	mDa	PPM	DBE	i-FIT	i-FIT (Norm)	Formula
423.9965	423.9954	1.1	2.6	22.5	689.2	0.9	C20 H2 N5 O7
	423.9968	-0.3	-0.7	11.5	689.3	1.0	C16 H13 N2 O8 63Cu
	423.9949	1.6	3.8	24.5	689.9	1.6	C28 H9 O 63Cu

Elemental Composition Report

Page 1

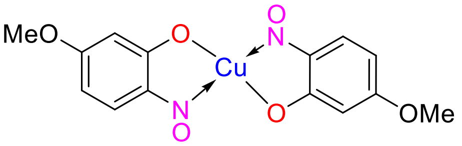

2h, 86% yield

Single Mass Analysis

Tolerance = 5.0 mDa / DBE: min = -1.5, max = 50.0
Element prediction: Off
Number of isotope peaks used for i-FIT = 3

Monoisotopic Mass, Odd and Even Electron Ions
366 formula(e) evaluated with 7 results within limits (up to 50 closest results for each mass)
Elements Used:
C: 0-35 H: 0-60 N: 0-4 O: 0-8 63Cu: 0-1

LCT Premier 350 °C AJN_3OME_4169 116 (0.929) Cm (116:122-(24:84+213:246))
1: TOF MS AP+

7.84e+003

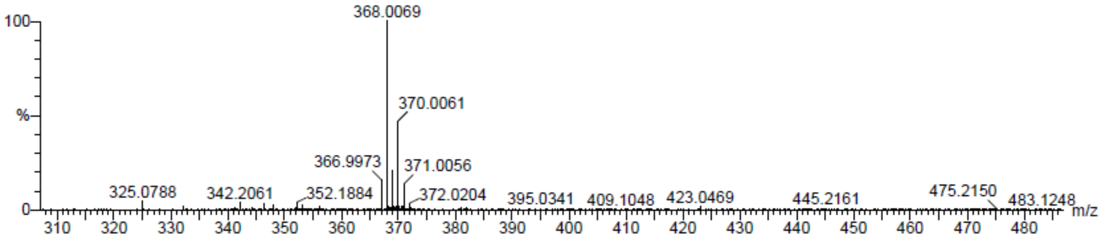

Minimum:				-1.5			
Maximum:		5.0	5.0	50.0			
Mass	Calc. Mass	mDa	PPM	DBE	i-FIT	i-FIT (Norm)	Formula
366.9973	366.9991	-1.8	-4.9	10.0	209.4	1.8	C14 H12 N2 O6 63Cu
	366.9991	-1.8	-4.9	20.5	209.4	1.7	C20 H3 N2 O6
	366.9951	2.2	6.0	16.5	209.8	2.1	C15 H3 N4 O8
	366.9951	2.2	6.0	6.0	209.9	2.3	C9 H12 N4 O8 63Cu
	366.9933	4.0	10.9	19.0	209.9	2.3	C21 H8 N2 O 63Cu
	367.0018	-4.5	-12.3	25.0	209.4	1.8	C23 H N3 O3
	367.0018	-4.5	-12.3	14.5	209.5	1.8	C17 H10 N3 O3 63Cu

Elemental Composition Report

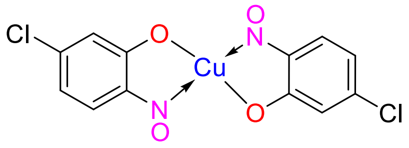

2i, 73% yield

Single Mass Analysis

Tolerance = 5.0 mDa / DBE: min = -1.5, max = 50.0
Element prediction: Off
Number of isotope peaks used for i-FIT = 3

Monoisotopic Mass, Even Electron Ions
1011 formula(e) evaluated with 8 results within limits (up to 50 closest results for each mass)
Elements Used:
C: 0-35 H: 0-60 N: 0-3 O: 0-5 Cl: 0-2 I: 0-2 63Cu: 0-1

LCT Premier 350 °C AJN_3C_4149 98 (0.786) Cm (93:104)
1: TOF MS AP+ 1.33e+005

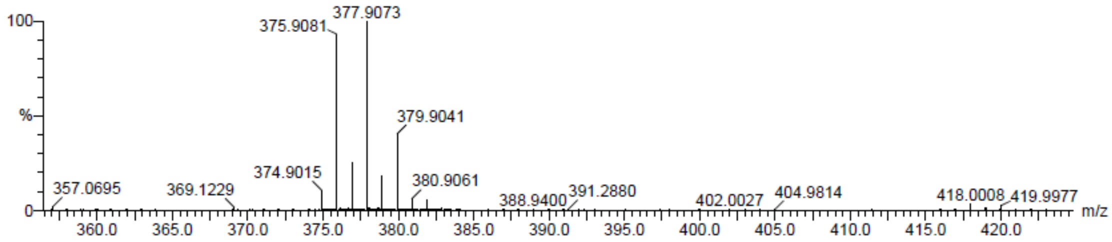

Minimum:				-1.5			
Maximum:		5.0	5.0	50.0			

Mass	Calc. Mass	mDa	PPM	DBE	i-FIT	i-FIT (Norm)	Formula
375.9081	375.9079	0.2	0.5	9.5	655.7	1.3	C12 H7 N2 O4 Cl2 63Cu
	375.9101	-2.0	-5.3	18.5	657.8	3.4	C18 H2 N2 O2 Cl 63Cu
	375.9059	2.2	5.9	2.5	660.0	5.6	C8 H12 N I2
	375.9107	-2.6	-6.9	13.5	659.6	5.2	C14 H3 N O4 I
	375.9112	-3.1	-8.2	-1.5	658.5	4.0	C5 H15 N2 O3 Cl I 63Cu
	375.9117	-3.6	-9.6	4.5	656.2	1.7	C8 H9 N3 O2 Cl2 I
	375.9119	-3.8	-10.1	13.5	655.4	1.0	C17 H7 O2 Cl2 63Cu
	375.9031	5.0	13.3	-1.5	656.7	2.2	C6 H16 N2 Cl2 I 63Cu

Elemental Composition Report

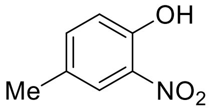

3a, 14 % yield

Single Mass Analysis

Tolerance = 5.0 mDa / DBE: min = -1.5, max = 50.0
Element prediction: Off
Number of isotope peaks used for i-FIT = 3

Monoisotopic Mass, Odd and Even Electron Ions
114 formula(e) evaluated with 5 results within limits (up to 50 closest results for each mass)
Elements Used:
C: 0-50 H: 0-80 N: 0-5 O: 0-8 Br: 0-2 127I: 0-1

LCT Premier 350 °C AJN_4M2NP_3665 47 (0.376) Cm (45:47)
1: TOF MS AP+ 9.84e+002

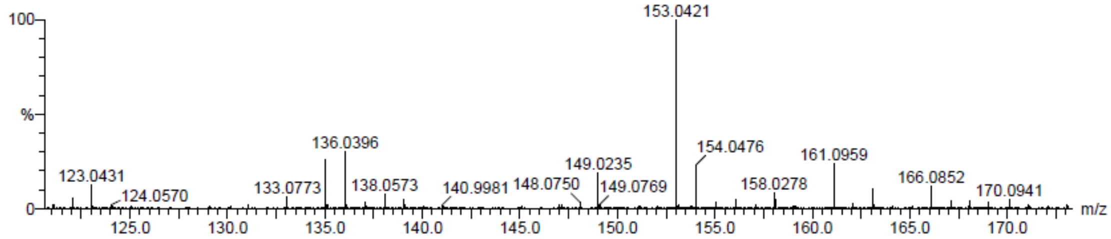

Minimum:				-1.5			
Maximum:		5.0	5.0	50.0			

Mass	Calc. Mass	mDa	PPM	DBE	i-FIT	i-FIT (Norm)	Formula
153.0421	153.0426	-0.5	-3.3	5.0	159.5	1.5	C7 H7 N O3
	153.0413	0.8	5.2	5.5	160.4	2.5	C5 H5 N4 O2
	153.0399	2.2	14.4	0.5	160.3	2.4	C4 H9 O6
	153.0453	-3.2	-20.9	9.5	158.5	0.5	C10 H5 N2
	153.0386	3.5	22.9	1.0	161.4	3.4	C2 H7 N3 O5

Elemental Composition Report

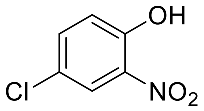

3b, 5 % yield

Single Mass Analysis
Tolerance = 3.0 mDa / DBE: min = -1.5, max = 50.0
Element prediction: Off
Number of isotope peaks used for i-FIT = 3

Monoisotopic Mass, Even Electron Ions
552 formula(e) evaluated with 7 results within limits (up to 500 best isotopic matches for each mass)
Elements Used:
C: 0-60 H: 0-80 N: 0-10 O: 0-10 S: 0-3 Cl: 0-2
Alexander Nicholls

15-Mar-2016
4Cl-LNO2 638 (3.157) Cm (634:690)
1: TOF MS ES-
1.89e+004

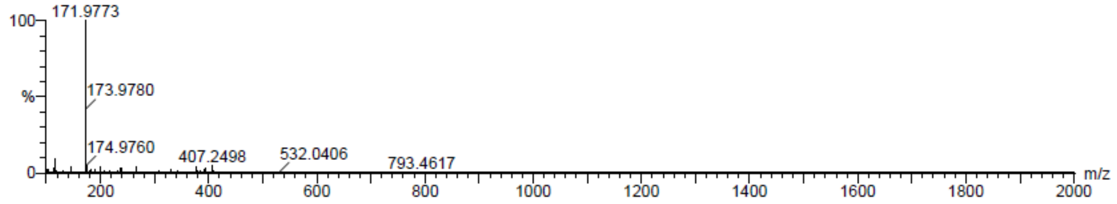

Minimum:				-1.5			
Maximum:		3.0	5.0	50.0			

Mass	Calc. Mass	mDa	PPM	DBE	i-FIT	i-FIT (Norm)	Formula
171.9773	171.9801	-2.8	-16.3	5.5	368.0	0.3	C6 H3 N O3 Cl
	171.9770	0.3	1.7	0.5	369.3	1.6	C2 H7 N3 S2 Cl
	171.9761	1.2	7.0	1.5	371.6	3.8	C H3 N3 O5 Cl
	171.9755	1.8	10.5	0.5	371.7	4.0	C4 H8 N S Cl2
	171.9793	-2.0	-11.6	1.5	373.0	5.2	C H4 N5 O Cl2
	171.9752	2.1	12.2	5.5	374.5	6.8	C3 H2 N5 S2
	171.9785	-1.2	-7.0	0.5	374.8	7.1	H6 N5 S3

Elemental Composition Report

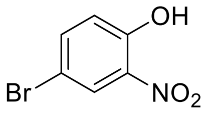

3c, 9 % yield

Page 1

Single Mass Analysis
Tolerance = 3.0 mDa / DBE: min = -1.5, max = 50.0
Element prediction: Off
Number of isotope peaks used for i-FIT = 3

Monoisotopic Mass, Even Electron Ions
636 formula(e) evaluated with 5 results within limits (up to 500 best isotopic matches for each mass)
Elements Used:
C: 0-60 H: 0-80 N: 0-10 O: 0-10 23Na: 0-1 Br: 0-2 127I: 0-1

Alexander Nicholls

17-Mar-2016
4BRLNO2 656 (3.239) Cm (654:658)
1: TOF MS ES-
3.69e+003

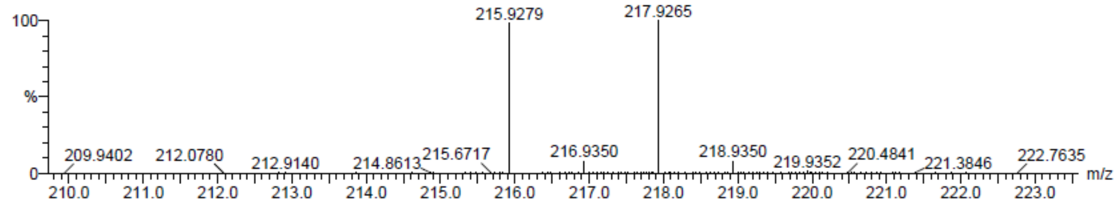

Minimum:				-1.5			
Maximum:		3.0	5.0	50.0			

Mass	Calc. Mass	mDa	PPM	DBE	i-FIT	i-FIT (Norm)	Formula
215.9279	215.9296	-1.7	-7.9	5.5	172.0	0.0	C6 H3 N O3 Br
	215.9272	0.7	3.2	2.5	176.2	4.1	C4 H4 N O3 23Na Br
	215.9256	2.3	10.7	1.5	180.6	8.5	C H3 N3 O5 Br
	215.9270	0.9	4.2	1.5	190.6	18.5	C H3 N3 O2 127I
	215.9286	-0.7	-3.2	2.5	191.1	19.1	C4 H4 N 23Na 127I

Elemental Composition Report

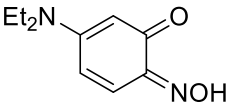

3g, 84 % yield

Single Mass Analysis

Tolerance = 3.0 mDa / DBE: min = -1.5, max = 100.0
Element prediction: Off
Number of isotope peaks used for i-FIT = 5

Monoisotopic Mass, Even Electron Ions
400 formula(e) evaluated with 3 results within limits (up to 500 closest results for each mass)
Elements Used:
C: 0-60 H: 0-50 N: 0-6 O: 0-8 S: 0-4

QToF Premier

26-Sep-2019
AJN_3NEt_L_135718 313 (2.668) Cm (313:330)

1: TOF MS ES+
9.77e+004

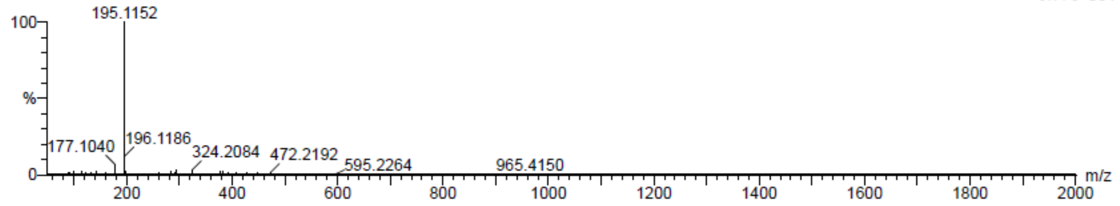

Mass	Calc. Mass	mDa	PPM	DBE	i-FIT	i-FIT (Norm)	Formula
Minimum:				-1.5			
Maximum:		3.0	5.0	100.0			
195.1152	195.1167	-1.5	-7.7	-0.5	842.0	6.7	C7 H19 N2 O2 S
	195.1134	1.8	9.2	4.5	835.3	0.0	C10 H15 N2 O2
	195.1174	-2.2	-11.3	8.5	840.4	5.1	C15 H15

Elemental Composition Report

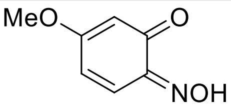

3h, 79 % yield

Single Mass Analysis

Tolerance = 5.0 mDa / DBE: min = -1.5, max = 50.0
Element prediction: Off
Number of isotope peaks used for i-FIT = 3

Monoisotopic Mass, Even Electron Ions
148 formula(e) evaluated with 4 results within limits (up to 500 best isotopic matches for each mass)
Elements Used:
C: 0-50 H: 0-50 N: 0-3 O: 0-9 S: 0-3 191Ir: 0-1

Alexander Nicholls

22-Feb-2016
AN3OMEL 232 (1.938) Cm (232:237)

1: TOF MS ES+
1.22e+004

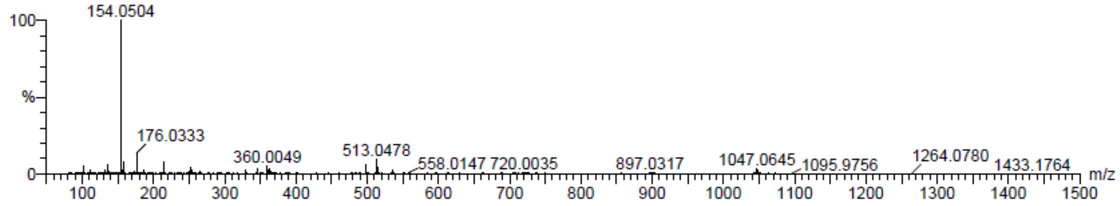

Mass	Calc. Mass	mDa	PPM	DBE	i-FIT	i-FIT (Norm)	Formula
Minimum:				-1.5			
Maximum:		5.0	5.0	50.0			
154.0504	154.0504	0.0	0.0	4.5	406.4	0.0	C7 H8 N O3
	154.0464	4.0	26.0	0.5	411.8	5.4	C2 H8 N3 O5
	154.0538	-3.4	-22.1	-0.5	415.5	9.1	C4 H12 N O3 S
	154.0473	3.1	20.1	-0.5	418.1	11.7	C3 H12 N3 S2

Elemental Composition Report

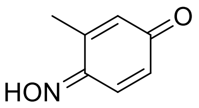

3j(i)

Single Mass Analysis

Tolerance = 5.0 PPM / DBE: min = -1.5, max = 50.0
Element prediction: Off
Number of isotope peaks used for i-FIT = 3

Monoisotopic Mass, Even Electron Ions
615 formula(e) evaluated with 3 results within limits (up to 500 best isotopic matches for each mass)
Elements Used:
C: 0-40 H: 0-80 10B: 0-1 N: 0-8 O: 0-8 F: 0-3 S: 0-1
Alexander Nicholls

11-Feb-2016
3M2NO 191 (1.597) Cm (187:195)

1: TOF MS ES+
1.14e+005

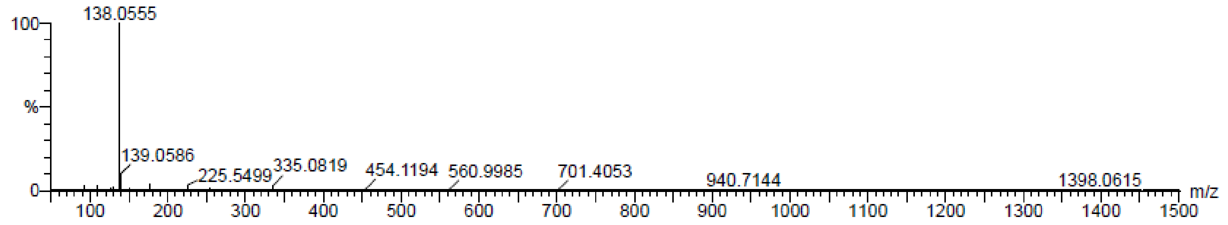

Mass	Calc. Mass	mDa	PPM	DBE	i-FIT	i-FIT (Norm)	Formula
Minimum:				-1.5			
Maximum:		5.0	5.0	50.0			
138.0555	138.0555	0.0	0.0	4.5	713.4	0.0	C7 H8 N O2
	138.0549	0.6	4.3	-0.5	728.3	15.0	C2 H9 10B N2 O F S
	138.0562	-0.7	-5.1	0.5	728.6	15.3	H8 N7 S

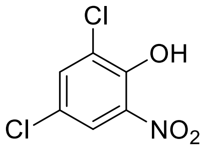

3k, 39% yield

Elemental Composition Report

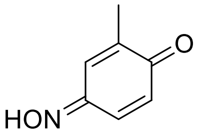

3l, 48 % yield

Single Mass Analysis
Tolerance = 5.0 PPM / DBE: min = -1.5, max = 50.0
Element prediction: Off
Number of isotope peaks used for i-FIT = 3

Monoisotopic Mass, Even Electron Ions
615 formula(e) evaluated with 3 results within limits (up to 500 best isotopic matches for each mass)
Elements Used:
C: 0-40 H: 0-80 10B: 0-1 N: 0-8 O: 0-8 F: 0-3 S: 0-1
Alexander Nicholls

11-Feb-2016
2M6NOP 216 (1.803) Cm (211:220)

1: TOF MS ES+
8.04e+004

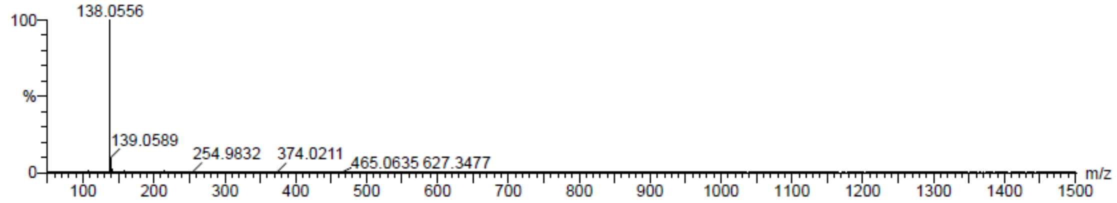

Minimum:				-1.5			
Maximum:		5.0	5.0	50.0			
Mass	Calc. Mass	mDa	PPM	DBE	i-FIT	i-FIT (Norm)	Formula
138.0556	138.0555	0.1	0.7	4.5	682.1	0.0	C7 H8 N O2
	138.0563	-0.7	-5.1	-0.5	688.8	6.6	C H8 10B N2 O5
	138.0562	-0.6	-4.3	0.5	694.1	11.9	H8 N7 S

Elemental Composition Report

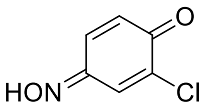

3m, 34% yield

Single Mass Analysis
Tolerance = 3.0 mDa / DBE: min = -1.5, max = 100.0
Element prediction: Off
Number of isotope peaks used for i-FIT = 5

Monoisotopic Mass, Even Electron Ions
426 formula(e) evaluated with 8 results within limits (up to 500 closest results for each mass)
Elements Used:
C: 0-60 H: 0-50 N: 0-6 O: 0-8 P: 0-3 Cl: 0-3
QToF Premier

24-Sep-2019
AJN_2CL_L_135596 294 (2.477) Cm (292:295)

1: TOF MS ES+
4.39e+004

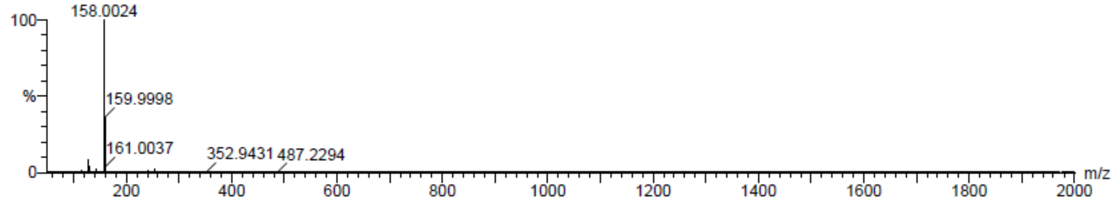

Minimum:				-1.5			
Maximum:		3.0	5.0	100.0			
Mass	Calc. Mass	mDa	PPM	DBE	i-FIT	i-FIT (Norm)	Formula
158.0024	158.0031	-0.7	-4.4	13.5	643.9	15.7	C12 N
	158.0037	-1.3	-8.2	4.5	642.2	14.0	C4 H6 N3 P2
	158.0009	1.5	9.5	4.5	628.2	0.0	C6 H5 N O2 Cl
	158.0007	1.7	10.8	4.5	642.7	14.5	C5 H5 N O3 P
	158.0000	2.4	15.2	0.5	639.5	11.3	C H6 N5 Cl2
	157.9999	2.5	15.8	0.5	636.5	8.3	H6 N5 O P Cl
	158.0049	-2.5	-15.8	0.5	643.7	15.5	H4 N3 O7
	158.0054	-3.0	-19.0	-0.5	642.9	14.7	C2 H11 N O P3

Elemental Composition Report

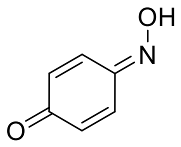

3o, 55% isolated mixture.

Single Mass Analysis
Tolerance = 10.0 mDa / DBE: min = -1.5, max = 50.0
Element prediction: Off
Number of isotope peaks used for i-FIT = 3

Monoisotopic Mass, Even Electron Ions
59 formula(e) evaluated with 3 results within limits (up to 500 best isotopic matches for each mass)
Elements Used:
C: 0-40 H: 0-40 N: 0-6 O: 0-4
Alex Nicholls

18-Mar-2016
2NOP 177 (1.500) Cm (177:197)

1: TOF MS ES+
2.00e+003

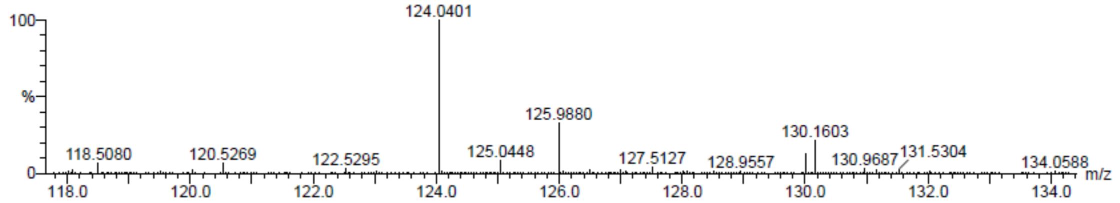

Minimum:				-1.5			
Maximum:		10.0	5.0	50.0			
Mass	Calc. Mass	mDa	PPM	DBE	i-FIT	i-FIT (Norm)	Formula
124.0401	124.0399	0.2	1.6	4.5	211.6	0.0	C6 H6 N O2
	124.0358	4.3	34.7	0.5	217.8	6.2	C H6 N3 O4
	124.0471	-7.0	-56.4	0.5	219.3	7.7	H6 N5 O3

Elemental Composition Report

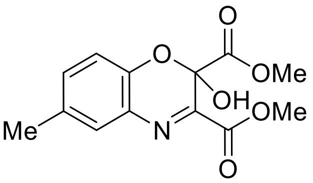

4a, 58% yield

Single Mass Analysis
Tolerance = 5.0 mDa / DBE: min = -1.5, max = 50.0
Element prediction: Off
Number of isotope peaks used for i-FIT = 3

Monoisotopic Mass, Even Electron Ions
429 formula(e) evaluated with 5 results within limits (up to 500 best isotopic matches for each mass)
Elements Used:
C: 0-40 H: 0-60 N: 0-8 O: 0-8 Br: 0-1

Alexander Nicholls

08-Feb-2016
MEDMAD 332 (2.807) Cm (332:337)

1: TOF MS ES+
3.82e+002

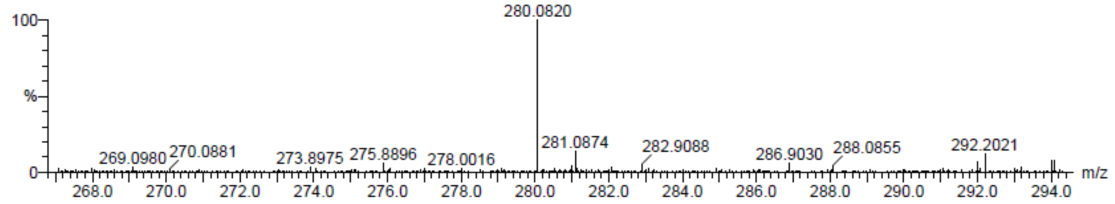

Minimum:				-1.5			
Maximum:		5.0	20.0	50.0			
Mass	Calc. Mass	mDa	PPM	DBE	i-FIT	i-FIT (Norm)	Formula
280.0820	280.0821	-0.1	-0.4	7.5	130.0	1.2	C13 H14 N O6
	280.0834	-1.4	-5.0	12.5	130.0	1.3	C14 H10 N5 O2
	280.0794	2.6	9.3	8.5	129.9	1.1	C9 H10 N7 O4
	280.0781	3.9	13.9	3.5	131.1	2.4	C8 H14 N3 O8
	280.0773	4.7	16.8	1.5	140.5	11.8	C8 H19 N5 O Br

Elemental Composition Report

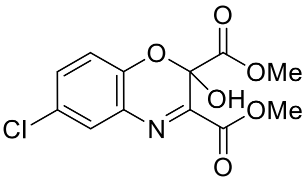

4b, 24 % yield

Page 1

Single Mass Analysis
Tolerance = 5.0 mDa / DBE: min = -1.5, max = 50.0
Element prediction: Off
Number of isotope peaks used for i-FIT = 3

Monoisotopic Mass, Even Electron Ions
712 formula(e) evaluated with 12 results within limits (up to 500 best isotopic matches for each mass)
Elements Used:
C: 0-40 H: 0-60 N: 0-8 O: 0-8 Cl: 0-2
Alexander Nicholls

08-Feb-2016
4CLH 360 (2.997) Cm (360:361)

1: TOF MS ES+
2.47e+002

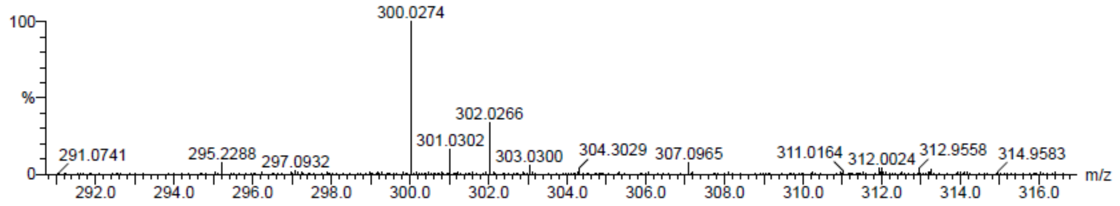

Minimum:				-1.5			
Maximum:		5.0	20.0	50.0			

Mass	Calc. Mass	mDa	PPM	DBE	i-FIT	i-FIT (Norm)	Formula
300.0274	300.0275	-0.1	-0.3	7.5	66.2	0.8	C12 H11 N O6 Cl
	300.0288	-1.4	-4.7	12.5	66.2	0.8	C13 H7 N5 O2 Cl
	300.0248	2.6	8.7	8.5	68.2	2.8	C8 H7 N7 O4 Cl
	300.0235	3.9	13.0	3.5	70.1	4.7	C7 H11 N3 O8 Cl
	300.0266	0.8	2.7	3.5	71.9	6.4	C7 H12 N5 O4 Cl2
	300.0307	-3.3	-11.0	7.5	72.4	6.9	C12 H12 N3 O2 Cl2
	300.0253	2.1	7.0	-1.5	72.8	7.4	C6 H16 N O8 Cl2
	300.0226	4.8	16.0	-0.5	75.3	9.8	C2 H12 N7 O6 Cl2
	300.0270	0.4	1.3	17.5	75.3	9.9	C14 H2 N7 O2
	300.0257	1.7	5.7	12.5	75.3	9.9	C13 H6 N3 O6
	300.0297	-2.3	-7.7	16.5	76.6	11.1	C18 H6 N O4
	300.0310	-3.6	-12.0	21.5	77.1	11.6	C19 H2 N5

Elemental Composition Report

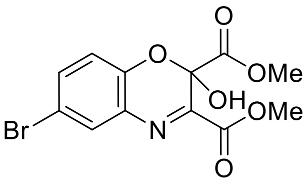

4c, 10% yield

Page 1

Single Mass Analysis
Tolerance = 5.0 mDa / DBE: min = -1.5, max = 50.0
Element prediction: Off
Number of isotope peaks used for i-FIT = 3

Monoisotopic Mass, Even Electron Ions
562 formula(e) evaluated with 5 results within limits (up to 500 best isotopic matches for each mass)
Elements Used:
C: 0-40 H: 0-60 N: 0-8 O: 0-8 Br: 0-1

Alexander Nicholls

08-Feb-2016
4BRH 367 (3.056) Cm (367:371)

1: TOF MS ES+
3.19e+002

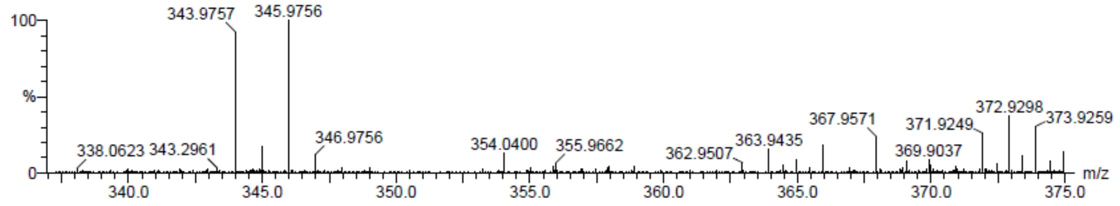

Minimum:				-1.5			
Maximum:		5.0	20.0	50.0			

Mass	Calc. Mass	mDa	PPM	DBE	i-FIT	i-FIT (Norm)	Formula
343.9757	343.9770	-1.3	-3.8	7.5	122.4	0.8	C12 H11 N O6 Br
	343.9743	1.4	4.1	8.5	124.5	2.9	C8 H7 N7 O4 Br
	343.9783	-2.6	-7.6	12.5	122.4	0.8	C13 H7 N5 O2 Br
	343.9730	2.7	7.8	3.5	126.3	4.7	C7 H11 N3 O8 Br
	343.9711	4.6	13.4	16.5	125.4	3.8	C19 H7 N O Br

Elemental Composition Report

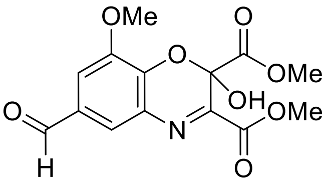

4d, 57% yield

Page 1

Single Mass Analysis

Tolerance = 3.0 mDa / DBE: min = -1.5, max = 100.0
Element prediction: Off
Number of isotope peaks used for i-FIT = 5

Monoisotopic Mass, Even Electron Ions
633 formula(e) evaluated with 4 results within limits (up to 500 closest results for each mass)
Elements Used:
C: 0-60 H: 0-50 N: 0-6 O: 0-8 S: 0-2

QToF Premier

23-Sep-2019
AJN_VAN_H_135501 720 (2.908) Cm (720:754)

1: TOF MS ES-
7.19e+003

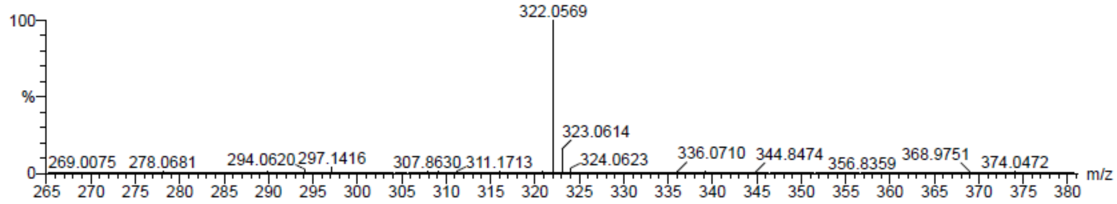

Minimum:				-1.5			
Maximum:		3.0	5.0	100.0			

Mass	Calc. Mass	mDa	PPM	DBE	i-FIT	i-FIT (Norm)	Formula
322.0569	322.0572	-0.3	-0.9	8.5	398.3	19.0	C15 H16 N O3 S2
	322.0563	0.6	1.9	9.5	379.3	0.0	C14 H12 N O8
	322.0576	-0.7	-2.2	14.5	385.1	5.8	C15 H8 N5 O4
	322.0597	-2.8	-8.7	4.5	394.0	14.7	C11 H16 N O8 S

Elemental Composition Report

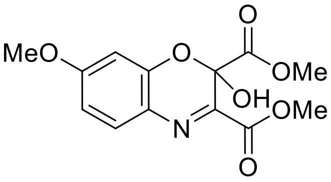

4h, 31% yield

Page 1

Single Mass Analysis

Tolerance = 3.0 mDa / DBE: min = -1.5, max = 100.0
Element prediction: Off
Number of isotope peaks used for i-FIT = 5

Monoisotopic Mass, Even Electron Ions
836 formula(e) evaluated with 6 results within limits (up to 500 closest results for each mass)
Elements Used:
C: 0-60 H: 0-50 N: 0-6 O: 0-8 S: 0-4

QToF Premier

26-Sep-2019
AJN_3OME_H_135690 343 (2.892) Cm (343:345)

1: TOF MS ES+
1.45e+005

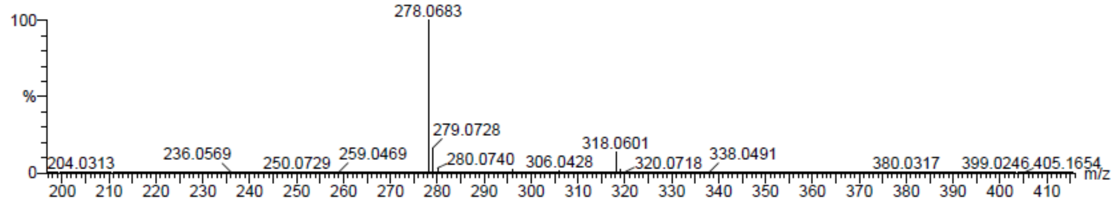

Minimum:				-1.5			
Maximum:		3.0	5.0	100.0			

Mass	Calc. Mass	mDa	PPM	DBE	i-FIT	i-FIT (Norm)	Formula
296.0790	296.0784	0.6	2.0	12.5	198.4	1.5	C14 H10 N5 O3
	296.0779	1.1	3.7	6.5	200.4	3.6	C14 H18 N O2 S2
	296.0804	-1.4	-4.7	2.5	199.3	2.5	C10 H18 N O7 S
	296.0770	2.0	6.8	7.5	197.5	0.7	C13 H14 N O7
	296.0813	-2.3	-7.8	1.5	202.2	5.4	C11 H22 N O2 S3
	296.0817	-2.7	-9.1	7.5	198.7	1.9	C11 H14 N5 O3 S

Elemental Composition Report

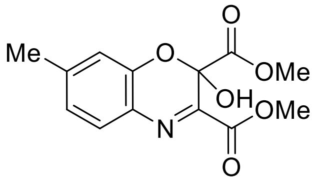

4j, > 9% yield

Page 1

Single Mass Analysis
Tolerance = 3.0 mDa / DBE: min = -1.5, max = 50.0
Element prediction: Off
Number of isotope peaks used for i-FIT = 3

Monoisotopic Mass, Even Electron Ions
3704 formula(e) evaluated with 30 results within limits (up to 500 best isotopic matches for each mass)
Elements Used:
C: 0-40 H: 0-80 N: 0-8 O: 0-8 F: 0-3 S: 0-1 127I: 0-1 23Na: 0-1

Alexander Nicholls

16-Feb-2016
3MEH 351 (2.921) Cm (342:357)

1: TOF MS ES+
4.65e+004

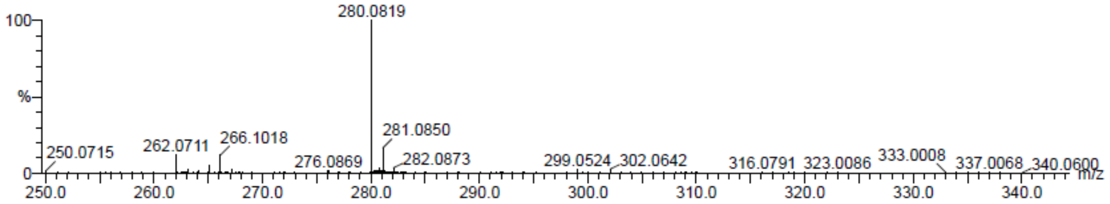

Minimum:				-1.5			
Maximum:		3.0	5.0	50.0			
Mass	Calc. Mass	mDa	PPM	DBE	i-FIT	i-FIT (Norm)	Formula
280.0819	280.0821	-0.2	-0.7	7.5	573.4	0.1	C13 H14 N O6
	280.0834	-1.5	-5.4	12.5	576.5	3.2	C14 H10 N5 O2
	280.0810	0.9	3.2	9.5	577.1	3.8	C12 H11 N5 O2 23Na
	280.0810	0.9	3.2	9.5	577.9	4.6	C12 H9 N5 F3
	280.0833	-1.4	-5.0	3.5	578.0	4.7	C10 H15 N O7 F
	280.0797	2.2	7.9	4.5	578.2	4.9	C11 H15 N O6 23Na
	280.0797	2.2	7.9	4.5	578.8	5.5	C11 H13 N O4 F3
	280.0846	-2.7	-9.6	8.5	579.1	5.8	C11 H11 N5 O3 F
	280.0822	-0.3	-1.1	5.5	579.3	6.0	C9 H12 N5 O3 F 23Na

IR Spectra of selected Compounds

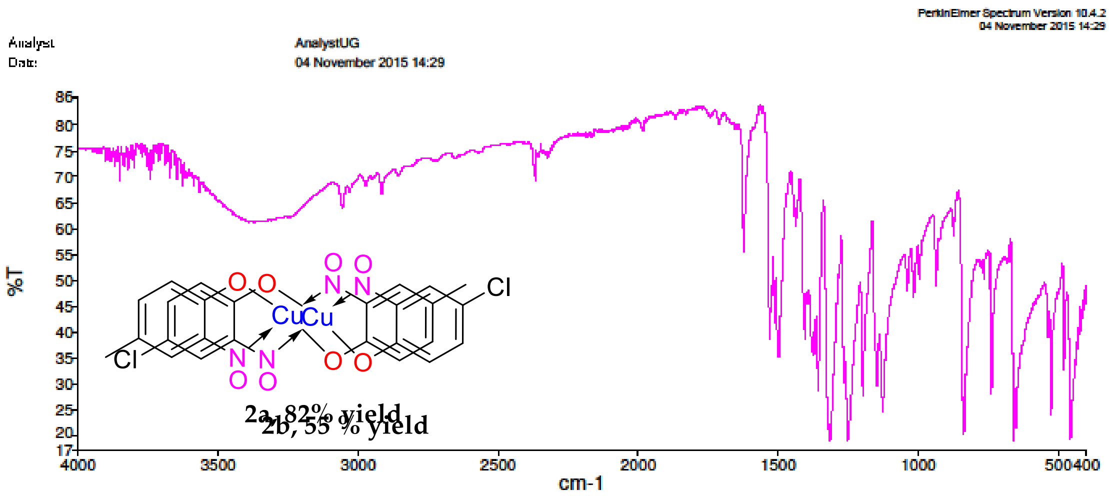

Sample Name	Description	Quality Checks
AJN_004	ME	The Quality Checks do not report any warnings for the sample.

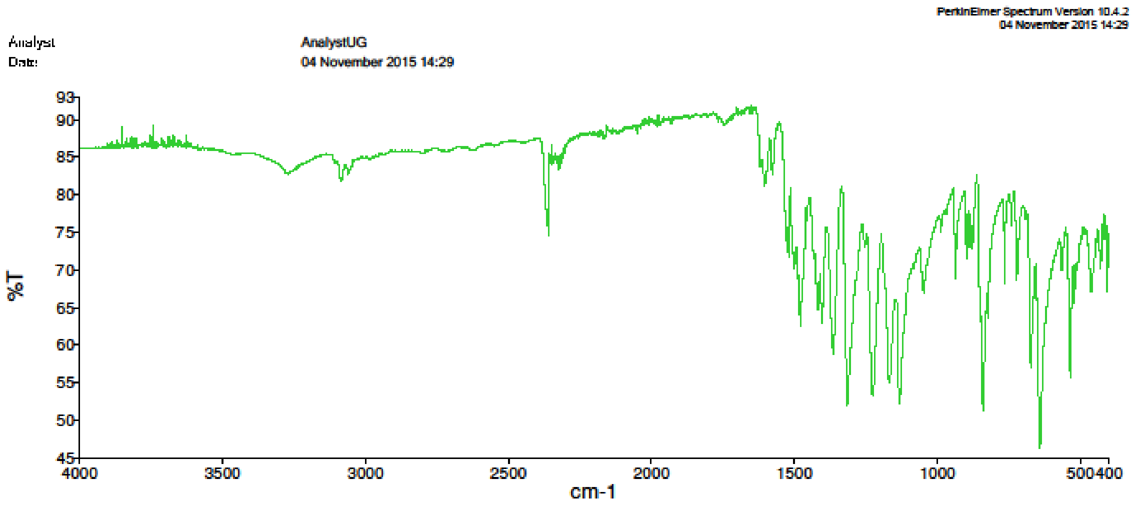

Sample Name	Description	Quality Checks
AJN_005	CL	The Quality Checks do not report any warnings for the sample.

PerkinElmer Spectrum Version 10.4.2
04 November 2015 14:30

Analyst AnalystUG
Date: 04 November 2015 14:30

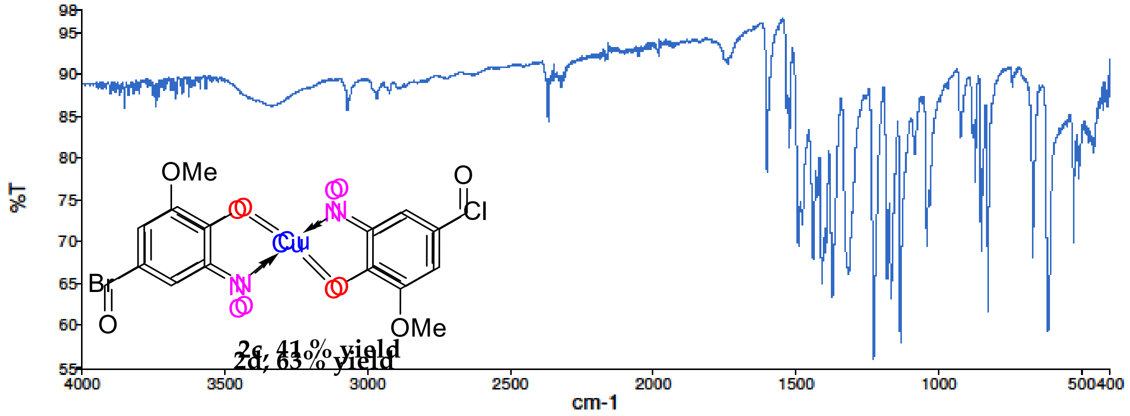

Sample Name	Description	Quality Checks
AJN_003	BR	The Quality Checks do not report any warnings for the sample.

PerkinElmer Spectrum Version 10.5.3
02 October 2019 14:17

Analyst AnalystUG
Date: 02 October 2019 14:17

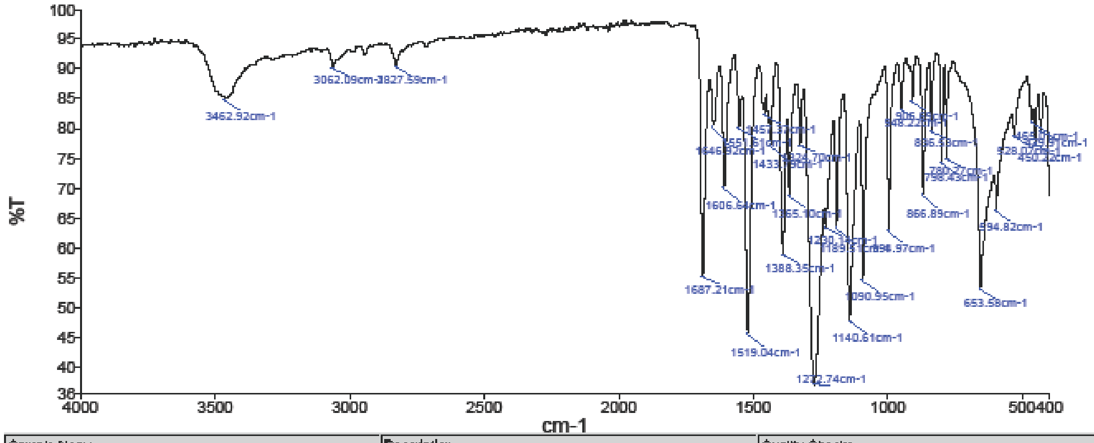

Sample Name	Description	Quality Checks
AnalystUG 918	Sample 918 By AnalystUG Date Wednesday, October 02 2019	The Quality Checks do not report any warnings for the sample.

PerkinElmer Spectrum Version 10.5.3
02 October 2019 14:19

Analyst AnalystUG
Date 02 October 2019 14:19

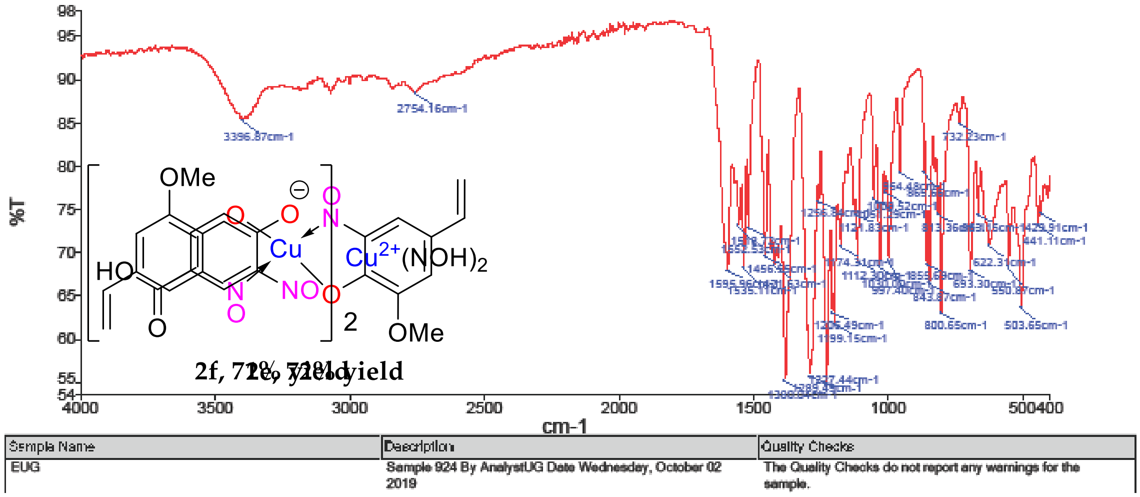

Sample Name	Description	Quality Checks
EUG	Sample 924 By AnalystUG Date Wednesday, October 02 2019	The Quality Checks do not report any warnings for the sample.

PerkinElmer Spectrum Version 10.5.3
02 October 2019 14:24

Analyst AnalystUG
Date 02 October 2019 14:24

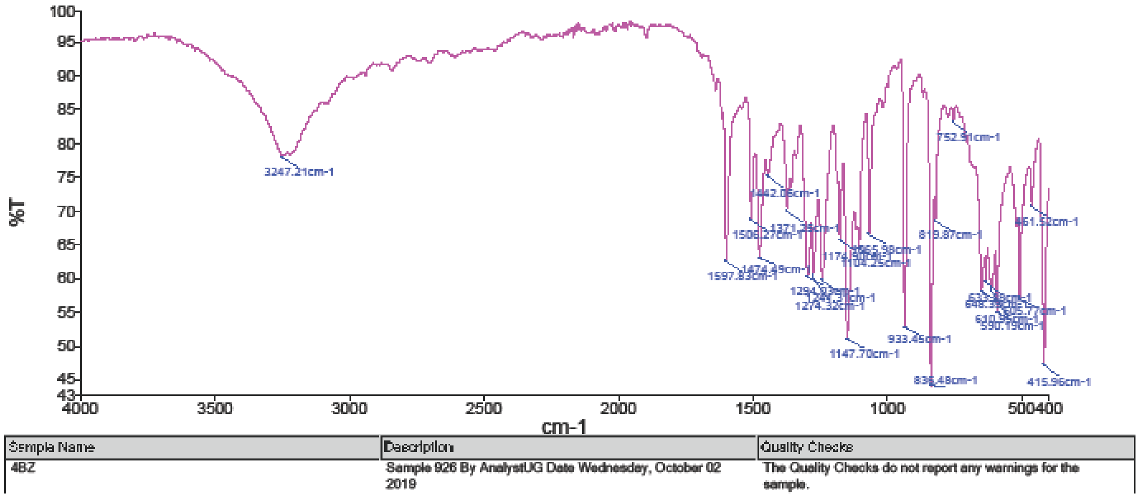

Sample Name	Description	Quality Checks
4BZ	Sample 926 By AnalystUG Date Wednesday, October 02 2019	The Quality Checks do not report any warnings for the sample.

PerkinElmer Spectrum Version 10.5.3
02 October 2019 14:26

Analyst AnalystUG
Date 02 October 2019 14:26

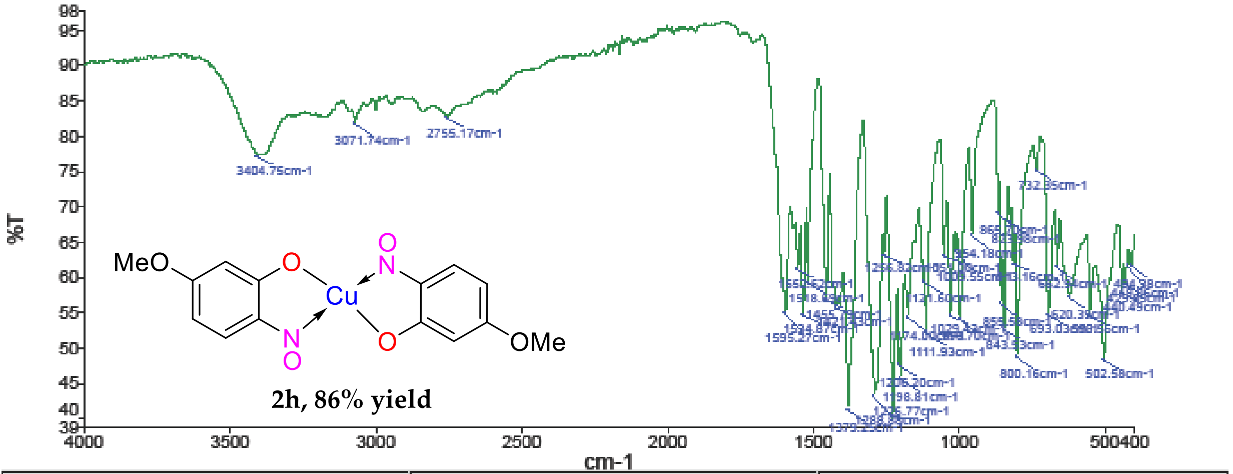

Sample Name	Description	Quality Checks
AnalystUG 928	Sample 928 By AnalystUG Date Wednesday, October 02 2019	The Quality Checks do not report any warnings for the sample.

PerkinElmer Spectrum Version 10.4.2
04 November 2015 14:31

Analyst AnalystUG
Date 04 November 2015 14:31

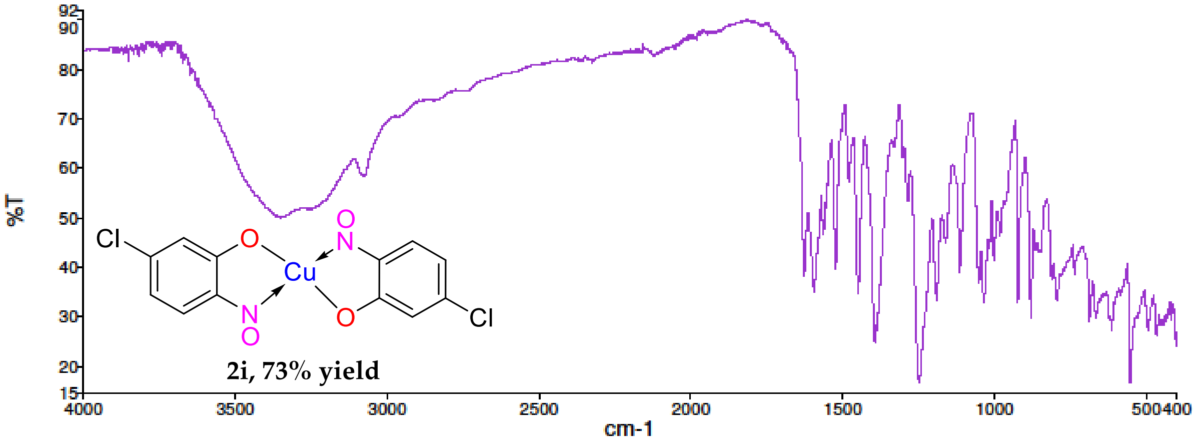

Sample Name	Description	Quality Checks
Alex N_001	3Ca	The Quality Checks do not report any warnings for the sample.

PerkinElmer Spectrum Version 10.4.2
10 February 2016 16:45

Analyst	AnalystUG
Date	10 February 2016 16:45

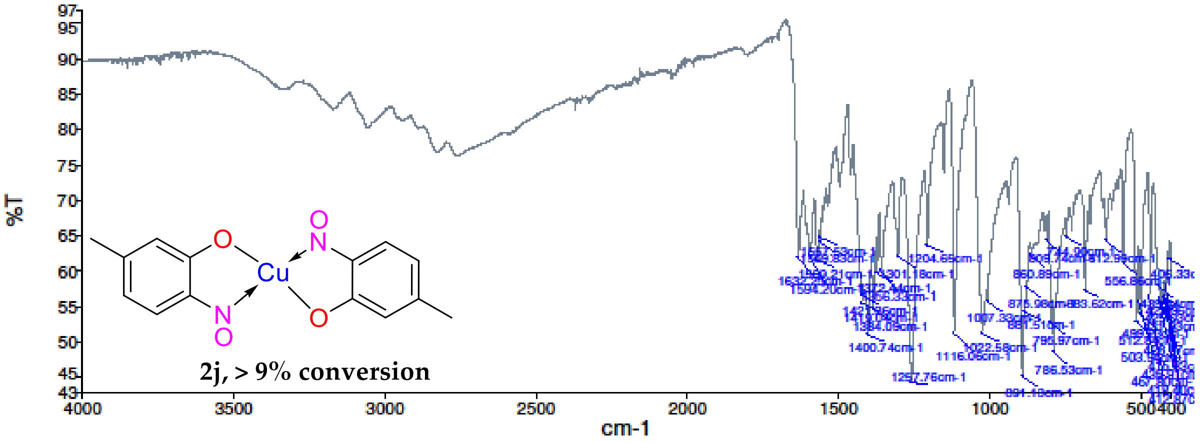

Sample Name	Description	Quality Checks
3M	AJN	The Quality Checks do not report any warnings for the sample.

PerkinElmer Spectrum Version 10.5.3
02 October 2019 14:22

Analyst	AnalystUG
Date	02 October 2019 14:22

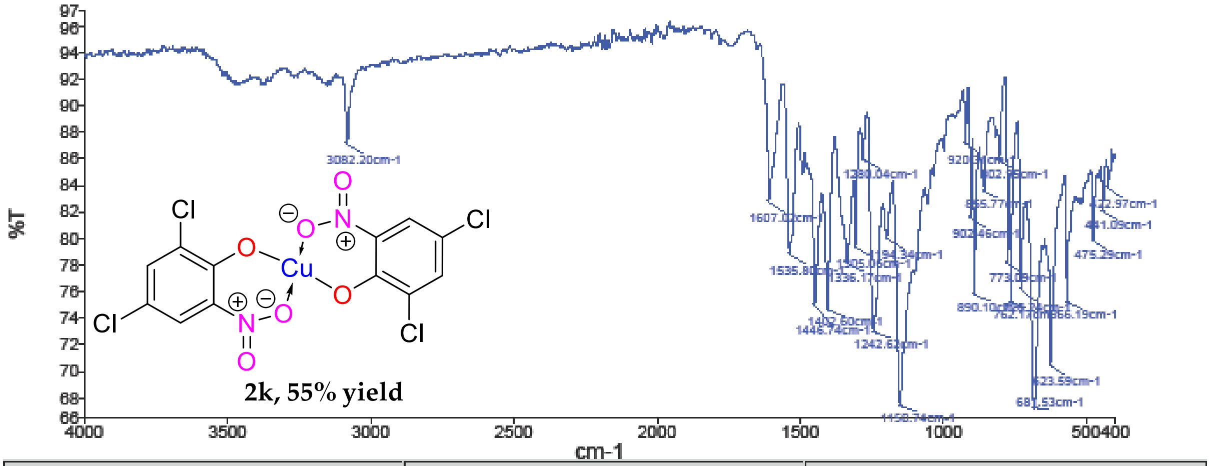

Sample Name	Description	Quality Checks
24CL	Sample 925 By AnalystUG Date Wednesday, October 02 2019	The Quality Checks give rise to a Weak Bands warning for the sample.

Page 1

PerkinElmer Spectrum Version 10.4.2
10 February 2016 16:48

Analyst	AnalystUG
Date	10 February 2016 16:48

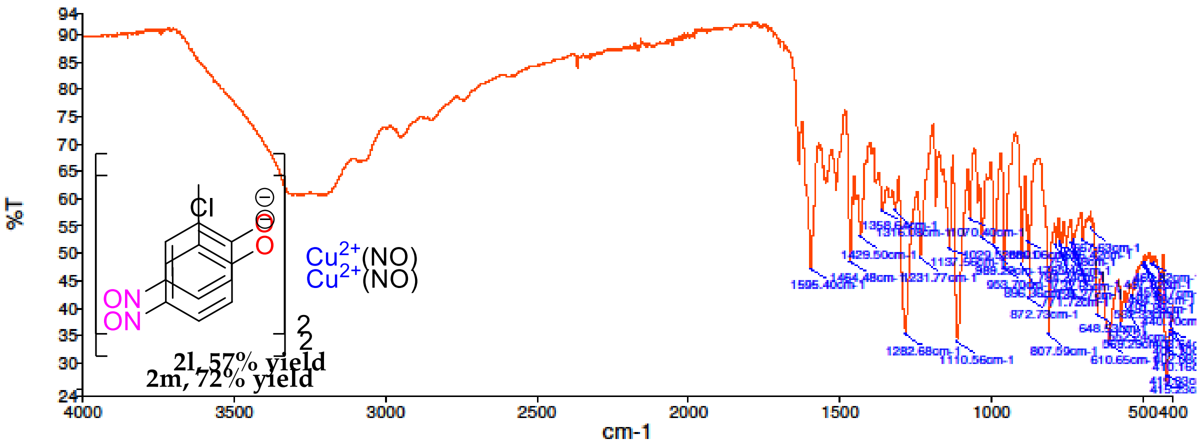

Sample Name	Description	Quality Checks
2M	Sample 904 By AnalystUG Date Wednesday, February 10 2016	The Quality Checks do not report any warnings for the sample.

PerkinElmer Spectrum Version 10.4.2
04 November 2015 14:39

Analyst	AnalystUG
Date	04 November 2015 14:39

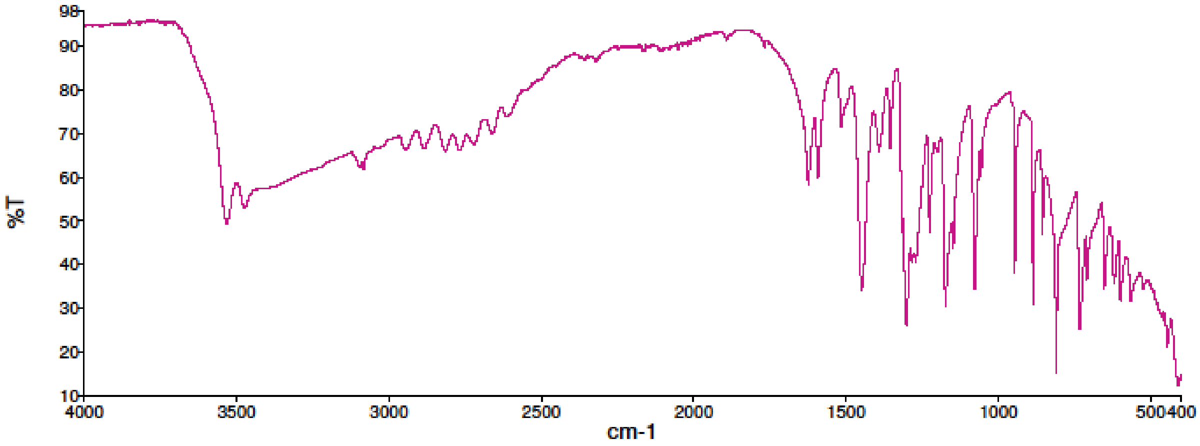

Sample Name	Description	Quality Checks
AJN_007	Sample 674 By AnalystUG Date Wednesday, November 04 2015	The Quality Checks do not report any warnings for the sample.

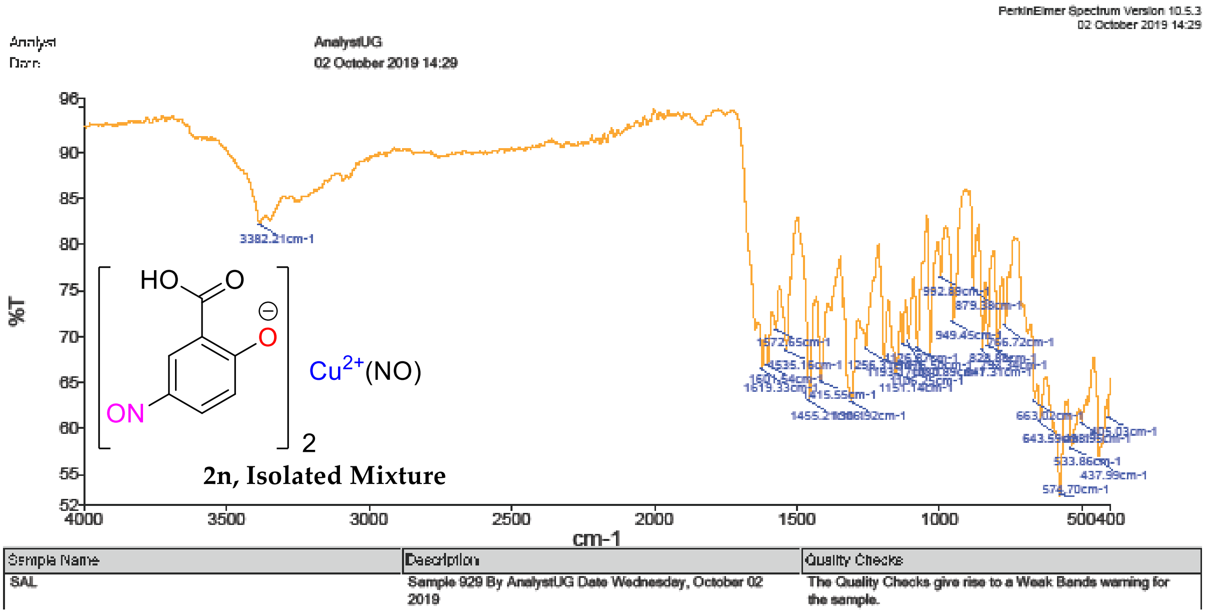
PerkinElmer Spectrum Version 10.5.3
02 October 2019 14:29
Analyst
Date
AnalystUG
02 October 2019 14:29
%T
cm-1
3382.21cm-1
HO
O
O
ON
Cu2+(NO)
2
2n, Isolated Mixture
Sample Name
SAL
Description
Sample 929 By AnalystUG Date Wednesday, October 02 2019
Quality Checks
The Quality Checks give rise to a Weak Bands warning for the sample.

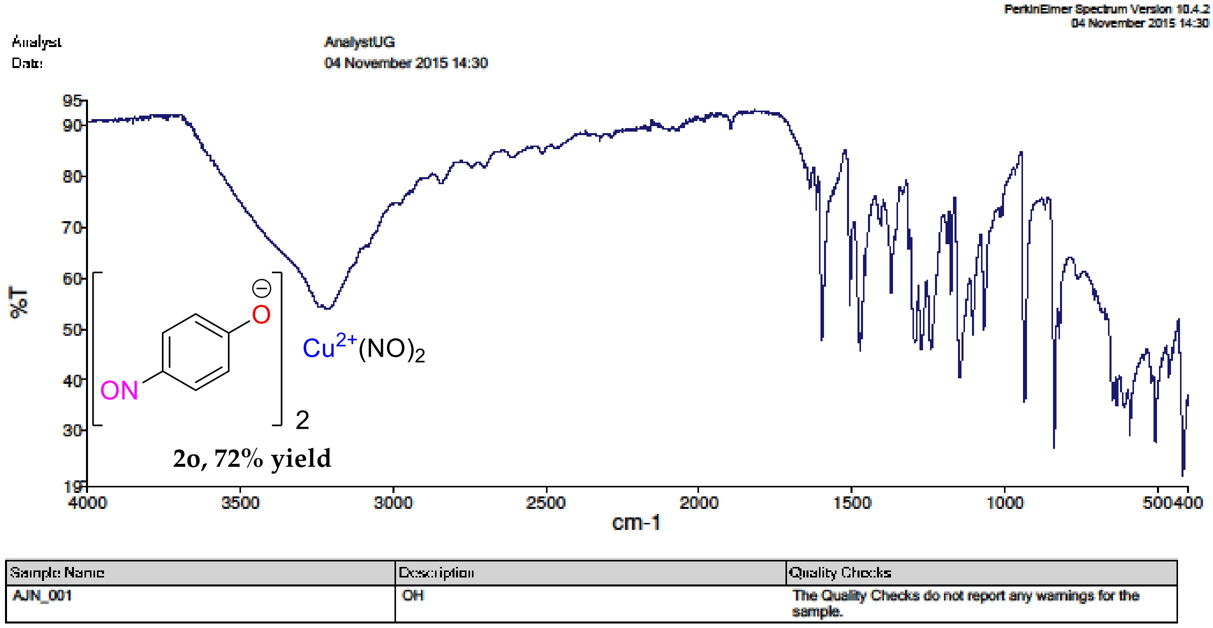
PerkinElmer Spectrum Version 10.4.2
04 November 2015 14:30
Analyst
Date
AnalystUG
04 November 2015 14:30
%T
cm-1
O
ON
Cu2+(NO)2
2
2o, 72% yield
Sample Name
AJN_001
Description
OH
Quality Checks
The Quality Checks do not report any warnings for the sample.

PerkinElmer Spectrum Version 10.4.2
03 February 2016 15:59

Analyst	AnalystUG
Date	03 February 2016 15:59

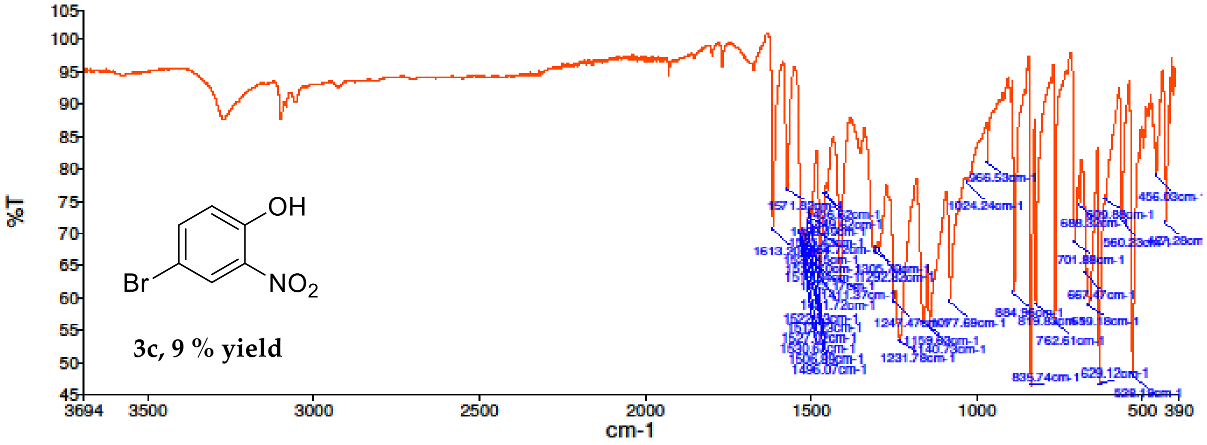

Sample Name	Description	Quality Checks
4-br-2-no2-phl		The Quality Checks do not report any warnings for the sample.

PerkinElmer Spectrum Version 10.4.2
10 February 2016 16:38

Analyst	AnalystUG
Date	10 February 2016 16:38

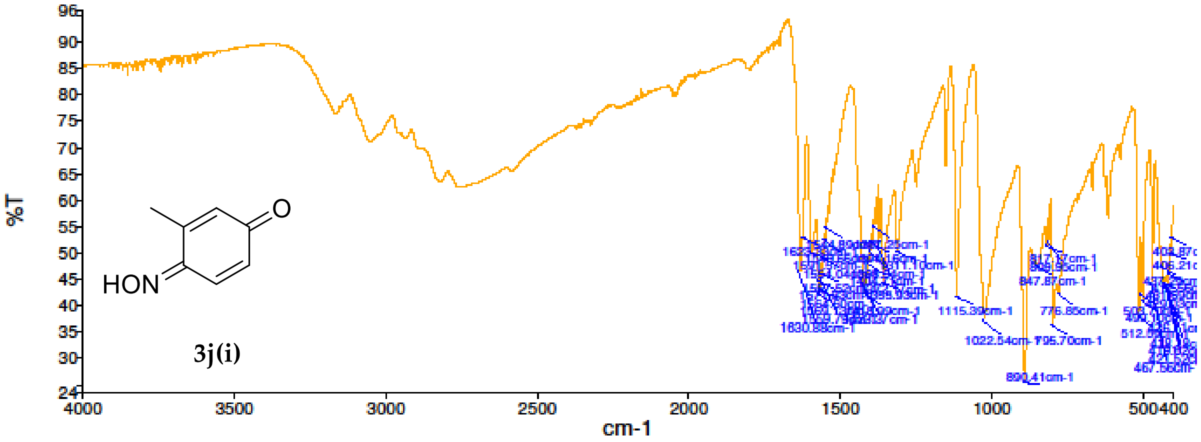

Sample Name	Description	Quality Checks
3M L	AJN	The Quality Checks do not report any warnings for the sample.

PerkinElmer Spectrum Version 10.4.2
10 February 2016 16:41

Analyst AnalystUG
Date: 10 February 2016 16:41

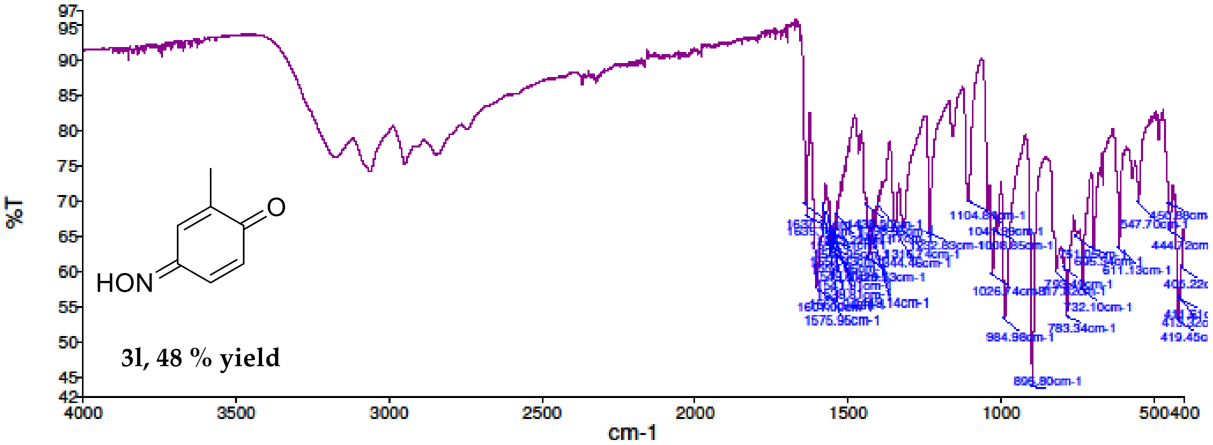

Sample Name	Description	Quality Checks
2ML	Sample 902 By AnalystUG Date Wednesday, February 10 2016	The Quality Checks do not report any warnings for the sample.

PerkinElmer Spectrum Version 10.4.2
03 February 2016 16:09

Analyst AnalystUG
Date: 03 February 2016 16:09

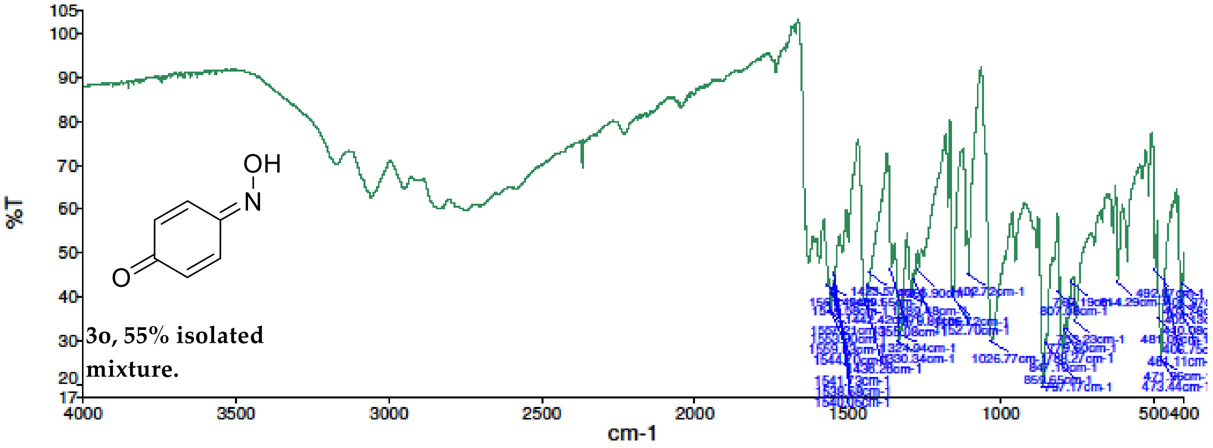

Sample Name	Description	Quality Checks
2-NITROSOPHENOL	Sample 896 By AnalystUG Date Wednesday, February 03 2016	The Quality Checks give rise to a Negative Bands warning for the sample.

PerkinElmer Spectrum Version 10.4.2
03 February 2016 16:03

Analyst	AnalystUG
Date	03 February 2016 16:03

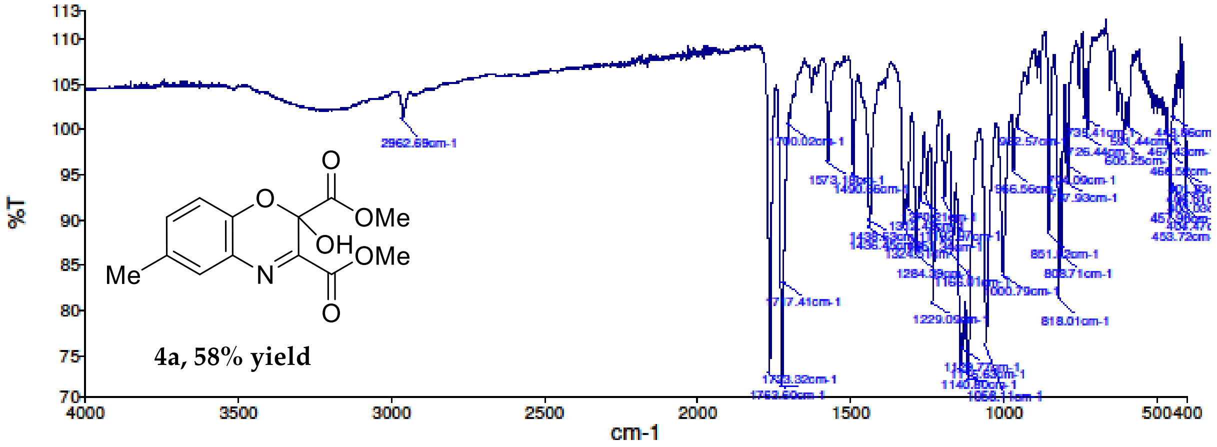

Sample Name	Description	Quality Checks
4ME Cycle	Sample 906 By AnalystUG Date Wednesday, February 03 2016	The Quality Checks give rise to a Baseline High warning for the sample.

PerkinElmer Spectrum Version 10.4.2
03 February 2016 16:06

Analyst	AnalystUG
Date	03 February 2016 16:06

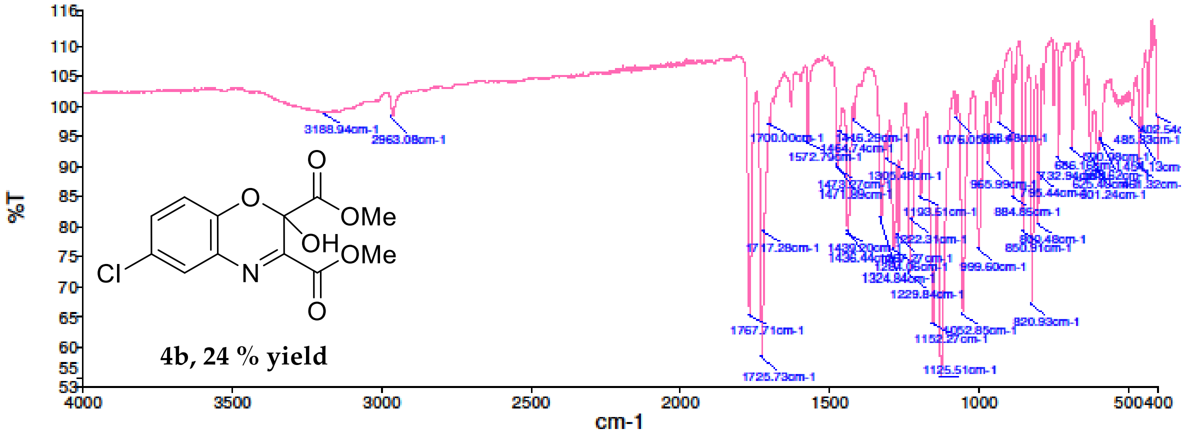

Sample Name	Description	Quality Checks
4CL CYCLE	Sample 895 By AnalystUG Date Wednesday, February 03 2016	The Quality Checks give rise to a Baseline High warning for the sample.

PerkinElmer Spectrum Version 10.5.3
02 October 2019 14:32

Analyst AnalystUG
Date 02 October 2019 14:32

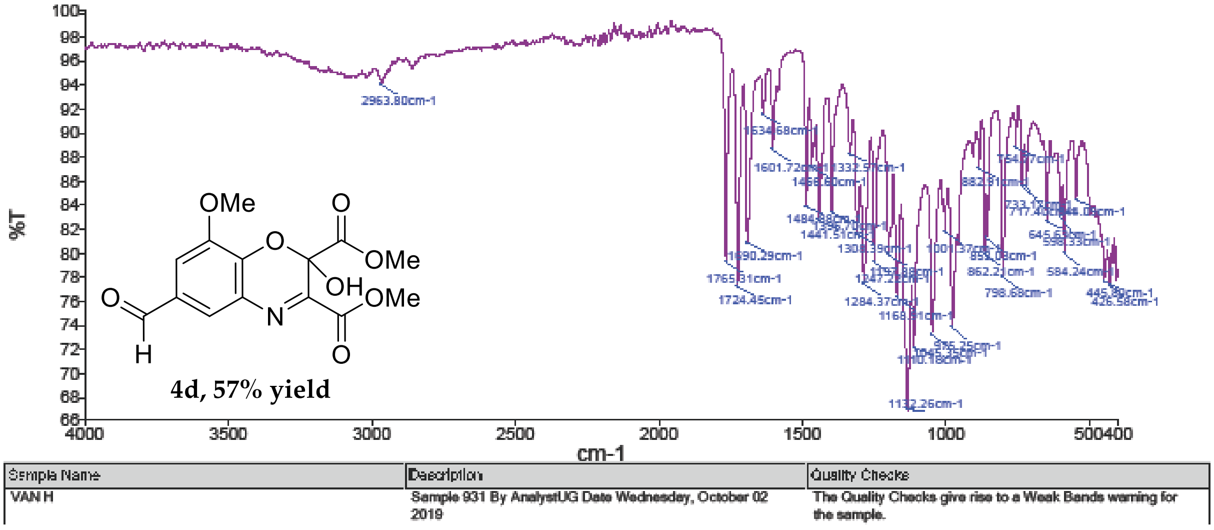

4d, 57% yield

Sample Name	Description	Quality Checks
VAN H	Sample 931 By AnalystUG Date Wednesday, October 02 2019	The Quality Checks give rise to a Weak Bands warning for the sample.

PerkinElmer Spectrum Version 10.5.3
02 October 2019 14:38

Analyst AnalystUG
Date 02 October 2019 14:38

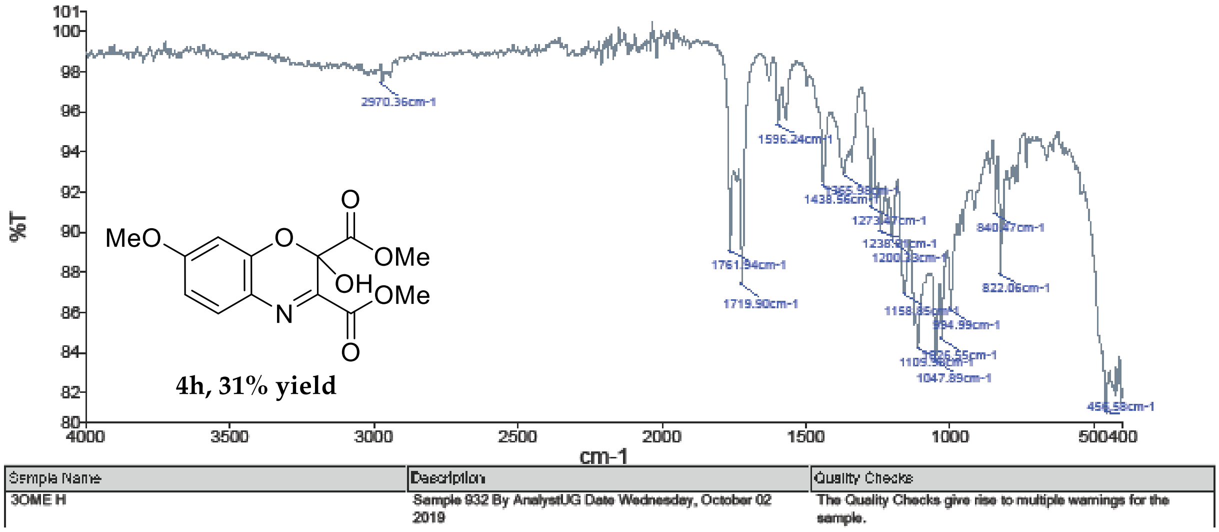

4h, 31% yield

Sample Name	Description	Quality Checks
3OME H	Sample 932 By AnalystUG Date Wednesday, October 02 2019	The Quality Checks give rise to multiple warnings for the sample.
